# Vapor Deposition Engineering for Thin-Film Microbatteries: From Nanoscale Ionics to Interface-Integrated Architectures

**DOI:** 10.1007/s40820-025-02002-w

**Published:** 2026-01-05

**Authors:** Mingming Zheng, Xinrui Xu, Xiaofei Wang, Haibin Lin, Changmin Hou, Mustafa Khan, Jinlong Zhu, Songbai Han

**Affiliations:** 1 Shenzhen Key Laboratory of Solid State Batteries and Guangdong Provincial Key Laboratory of Energy Materials for Electric Power and Guangdong-Hong Kong-Macao Joint Laboratory for Photonic-Thermal-Electrical Energy Materials and Devices and Institute of Major Scientific Facilities for New Materials and Academy for Advanced Interdisciplinary Studies, Southern University of Science and Technology, Shenzhen, 518055 People’s Republic of China; 2 State Key Laboratory of Inorganic Synthesis and Preparative Chemistry, Jilin University, Changchun, 130012 People’s Republic of China; 3 Department of Physics and State Key Laboratory of Quantum Functional Materials and Guangdong Basic Research, Center of Excellence for Quantum Science, Southern University of Science and Technology, Shenzhen, 518055 People’s Republic of China

**Keywords:** Thin-film microbatteries, Vapor-phase deposition techniques, Nanoscale ionic conductivity, Interfacial engineering, Microdevice integration

## Abstract

The rapid proliferation of microelectronics, coupled with the advent of the internet of things (IoT) era, has created an urgent demand for miniaturized, integrable, and reliable on-chip energy storage systems. All-solid-state thin-film microbatteries (TFMBs), distinguished by their intrinsic safety, compact design, and compatibility with microfabrication techniques, have emerged as promising candidates to power next-generation IoT devices. Nevertheless, in contrast to the well-established development of conventional lithium-ion batteries, the advancement of TFMBs remains at an early stage, facing persistent challenges in materials innovation, interface optimization, and scalable manufacturing. This review critically examines the pivotal role of vapor deposition technologies, including magnetron sputtering, pulsed laser deposition, thermal/electron-beam evaporation, chemical vapor deposition, and atomic layer deposition, in the fabrication and performance modulation of TFMBs. We systematically summarize recent progress in thin-film electrodes and solid-state electrolytes, with particular emphasis on how deposition parameters dictate crystallinity, lattice orientation, and ionic transport in functional layers. Furthermore, we highlight strategies for solid–solid interface engineering, three-dimensional structural design, and multifunctional integration to enhance capacity retention, cycling stability, and interfacial compatibility. Looking ahead, TFMBs are expected to evolve toward multifunctional platforms, exhibiting mechanical flexibility, optical transparency, and hybrid energy-harvesting compatibility, thereby meeting the heterogeneous energy requirements of future IoT ecosystems. Overall, this review provides a comprehensive perspective on vapor-phase-enabled TFMB technologies, delivering both theoretical insights and technological guidelines for the scalable realization of high-performance microscale power sources.

## Highlights

- Tailored crystallinity and defect engineering in ultrathin solid-state electrolytes enable enhanced nanoscale ion transport.
- Chemically stable and conformal interfaces mitigate interfacial failure and space charge effects in microbattery architectures.
- Spatial atomic layer deposition and scalable vapor-phase strategies enable 3D integration and monolithic interfacing of thin-film microbatteries with internet of things device platforms.

## Graphical abstract

**Figure:**
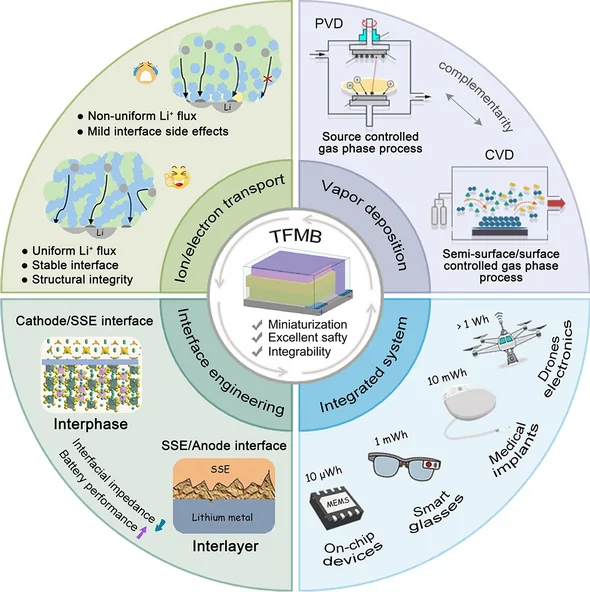

## Introduction

The rapid miniaturization of electronic systems is driving a global transition toward the internet of things (IoT), in which densely networked, autonomous microscale devices, including microelectromechanical systems (MEMS), radio-frequency identification (RFID) tags, sensors, smart cards, and implantable electronics, are reshaping paradigms of energy storage and delivery at the microscale [1–7]. Such devices impose stringent requirements on power sources, demanding high energy and power densities, long-term reliability, compact footprints, and seamless compatibility with microfabrication processes. Conventional lithium-ion batteries (LIBs) employing liquid electrolytes are intrinsically limited by leakage risks, bulky form factors, and poor compatibility with microsystem integration [8–15]. In contrast, all-solid-state thin-film microbatteries (TFMBs), fabricated through sequential vapor deposition of cathode, solid-state electrolyte (SSE), and anode layers on microdevice-compatible substrates, provide a compelling pathway toward safe, compact, and integrable energy solutions for next-generation microsystems [16–22]. With total thickness below 1 mm and volumes below 1000 mm^3^ [23], TFMBs offer superior safety, design flexibility, and mechanical robustness, rendering them highly attractive for on-chip applications ranging from IoT terminals and biomedical implants to flexible electronics and aerospace platforms [24–29].

Among the enabling technologies for TFMB fabrication, vapor-phase deposition techniques, including magnetron sputtering (MS), pulsed laser deposition (PLD), thermal and e-beam evaporation, chemical vapor deposition (CVD), and atomic layer deposition (ALD), play a pivotal role [30–40]. These methods provide atomic-level thickness control, excellent film uniformity, and compatibility with low-temperature microfabrication, thereby enabling precise engineering of electrode/electrolyte interfaces and the construction of multilayer functional architectures [8, 41–43]. Compared with solution-based methods, vapor-phase techniques offer enhanced densification, structural integrity, and interfacial ion transport, which are essential for long cycle life and high areal energy density within confined geometries [44, 45].

Despite significant progress, the practical deployment of TFMBs remains constrained by critical challenges. A major limitation lies in restricted areal capacity, as the micrometer-scale thickness of electrodes precludes the use of conductive additives and limits active material loading [46]. Recent strategies to address this issue include multilayer stacking, 3D structural design, and thick-film integration, all of which leverage the conformality and precision of vapor-phase deposition to enhance energy storage per footprint without compromising device integrity [8, 16]. In parallel, coupling TFMBs with energy-harvesting technologies such as photovoltaics, thermoelectrics, and piezoelectrics has enabled the creation of self-sustaining autonomous microsystems [47, 48]. Although the fundamental concept of TFMBs has existed for more than five decades, with the development of LiPON SSEs in the 1990s representing a milestone, their commercialization remains limited, primarily due to energy density bottlenecks, processing complexity, and integration barriers [49, 50]. These constraints underscore the urgent need for breakthroughs in material design, interfacial engineering, and scalable processing.

In this review, we present a comprehensive analysis of recent advances in vapor-deposited TFMBs, with a particular focus on structural design, interfacial modulation, and multifunctional integration of key material components. We systematically compare major vapor deposition technologies, evaluate their applicability for thin-film cathodes, SSEs, and anodes, and examine how deposition parameters influence film quality and electrochemical performance. Emerging design strategies, such as multilayer assembly, three-dimensional (3D) architectures, mechanical flexibility, and optical transparency, are also discussed, aiming to bridge the gap between laboratory-scale prototypes and practical microscale power solutions. Finally, we identify key scientific challenges and outline future research opportunities for developing TFMBs with higher areal capacity, extended cycle life, and broader microelectronic compatibility. By highlighting the central role of vapor-phase deposition in TFMB advancement, this review seeks to provide both fundamental insights and practical guidance for the scalable realization of high-performance on-chip power sources in the IoT era.

## Vapor Deposition Techniques

Vapor-phase engineering is central to the realization of high-performance all-solid-state TFMBs, where the rapid, uniform, and precisely controlled deposition of functional thin films is essential for achieving superior electrochemical properties, reliable interfacial stability, and scalable processing. Among various fabrication routes, PVD techniques, particularly MS and PLD, have been widely employed for constructing both electrode and SSE layers, owing to their capability to produce dense, conformal, and compositionally controlled films with nanoscale thickness precision. Their inherent compatibility with semiconductor microfabrication protocols further enables direct integration of TFMBs with microsystems and on-chip platforms.

**Fig. 1:**
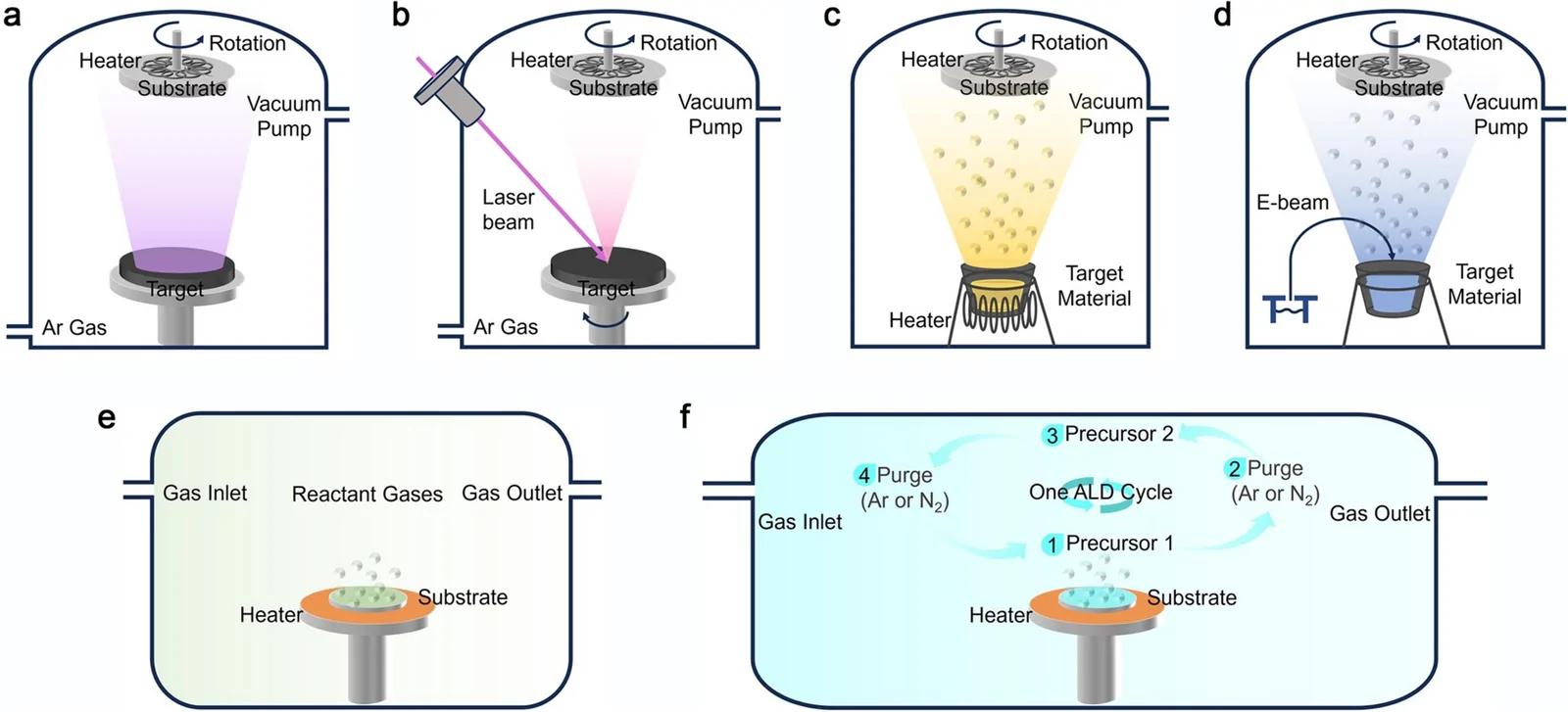
Schematic overview of representative vapor-phase thin-film deposition techniques widely applied in the fabrication of TFMBs. **a** Magnetron sputtering (MS) for depositing metals and oxides with high film uniformity and adhesion. **b** Pulsed laser deposition (PLD) enabling stoichiometric transfer and epitaxial growth of complex oxides. **c** Thermal evaporation as a cost-effective method suitable for low-melting-point materials. **d** Electron-beam evaporation for processing refractory materials with efficient energy delivery. **e** Chemical vapor deposition (CVD) for large-area deposition of dense and conformal thin films. **f** Atomic layer deposition (ALD) providing sub-nanometer thickness control and excellent step coverage in 3D architectures

Despite these advantages, PVD-based methods face limitations, including high capital cost, low material utilization efficiency, and restricted throughput in large-area manufacturing, which hinder their commercial scalability. Consequently, CVD and emerging vapor-phase strategies have attracted growing interest as complementary or alternative approaches, offering superior conformality, step coverage, and large-area uniformity. These attributes are particularly advantageous for fabricating thin-film electrodes and SSEs in intricate or 3D architectures. To provide a systematic overview, Fig. 1 schematically illustrates the working principles of representative vapor deposition techniques, while Table 1 compares their advantages, limitations, and application scopes. Together, these analyses serve as critical references for rational material selection, device design, and scalable TFMB manufacturing.

**Table 1:** Comparative overview of vapor deposition techniques for TFMB fabrication

Deposition technique	Deposition rate	Film uniformity	Typical applicable materials	Key advantages	Major limitations
MS	Slow-moderate	Moderate-High	Metals (Ag, Cu, Pt, Al, Ti, Ni), oxides (LCO, LMO, V_2_O_5_, LiPON)	Excellent film uniformity, compatible with refractory materials, broad material versatility, strong adhesion to substrates	Sophisticated instrumentation, low growth rate, risk of substrate damage, costly targets
PLD	Moderate	Moderate	Complex oxides (LLZO, LLTO, Li_3_OX)	Capability to fabricate heterostructures, stoichiometric transfer, flexible control of film thickness	Limited deposition area, volatile species loss, slow rate and particle splashing
Thermal evaporation	Fast	Low	Low-melting metals (Li), selected oxides (SiO_2_)	Simple setup, high film purity, scalable to large-area coatings, low cost	Poor thickness uniformity, unsuitable for refractory materials, high substrate temperature required, weak adhesion
E-beam evaporation	Moderate	Moderate-High	High-melting-point metals (Ti), conductive oxides (ITO, FTO)	Suitable for refractory metals, high-purity films, relatively fast rate	Expensive equipment, substrate damage by electron beam, limited step coverage
CVD	Fast	High	Silicon, carbon, oxides	High-quality density films, scalable to wafer-level deposition	Harsh conditions, toxic precursors, complex system maintenance
ALD	Very slow	Ultrahigh	Al_2_O_3_, HfO_2_, various functional coatings	Sub-Å thickness control, excellent step coverage, conformal coatings for 3D and high-aspect-ratio structures	Extremely low growth rate, limited precursors, high process cost

### Physical Vapor Deposition (PVD)

PVD encompasses a class of high-vacuum thin-film techniques in which material is vaporized from a solid source and condensed onto a substrate, enabling dense and uniform films with nanometer-scale precision. Common approaches include MS, PLD, and thermal or e-beam evaporation, each offering distinct merits in terms of film quality, composition control, and process adaptability. MS and thermal evaporation are particularly favored in industrial applications such as microelectronics and optoelectronics, given their compatibility with multilayer device architectures and roll-to-roll processing. PVD methods provide precise control over thickness, stoichiometry, and microstructure, thereby supporting the engineering of complex electrode–electrolyte interfaces and multilayer configurations. Nonetheless, throughput, cost, and scalability challenges remain critical barriers, motivating the pursuit of alternative or hybrid deposition routes.

#### Magnetron Sputtering (MS)

MS is one of the most widely utilized PVD techniques, enabling uniform and dense thin films across diverse materials. In MS, energetic ions (typically Ar^+^), generated within a plasma bombard a solid target, ejecting atoms that condense on the substrate to form a film (Fig. 1a). By tuning the discharge mode, DCMS is applied for conductive materials, while RFMS is used for insulators. Furthermore, co-sputtering allows independent power control over multiple targets, facilitating the synthesis of doped or multi-component films, such as oxides, nitrides, and alloys, an essential feature for electrode optimization. MS has been widely used for the deposition of electrodes (e.g., LMO [51], LCO [52, 53], LTO [54], TiO_2_ [55], Si [56, 57]) and SSEs (e.g., LiPON [58–61], LLZO [62], LATP [63]). It has also enabled 3D nanostructures, such as vertically aligned LMO nanowalls, which improve surface area and electrochemical kinetics [64, 65].

Films deposited by MS are generally amorphous at room or low temperatures. Although amorphous electrolytes exhibit lower ionic conductivity than crystalline analogues, they provide significant benefits, including alleviated stress accumulation, improved interfacial conformity, and enhanced cycling stability. For instance, RF-sputtered Li_3_PO_4_ films retain amorphous phases with ionic conductivities above 10^−6^ S cm^−1^, while suppressing crystallization-induced interfacial resistance [66]. Similarly, amorphous nitrogen-doped Li–Al–Ti–P–O–N films demonstrate dense microstructures and stable cycling [67, 68]. Compared with high-temperature PLD or CVD processes that often yield crystalline phases prone to cracking or grain boundary degradation, MS produces smooth and conformal amorphous films that effectively suppress side reactions and electrolyte penetration, thereby improving coulombic efficiency. Moreover, deposition parameters (e.g., power, pressure, gas composition) can be tailored to modulate film density, defects, and stress states, ultimately tuning Li^+^ transport in isotropic amorphous matrices.

Despite these advantages, MS faces inherent drawbacks: (1) moderate deposition rates limit throughput for thick films (≥ 1 μm), (2) complex vacuum systems raise cost, (3) lithium volatility may cause non-stoichiometry and interfacial degradation, and (4) poor step coverage hinders deposition on high-aspect-ratio 3D structures. Thus, while MS remains indispensable for lab-scale TFMB prototyping, further optimization is necessary for industrial scalability.

#### Pulsed Laser Deposition (PLD)

PLD employs high-energy laser pulses to ablate a solid target, generating a transient plasma plume of atoms, ions, and clusters that deposit onto a substrate (Fig. 1b). The process offers several advantages: (1) excellent stoichiometric transfer, particularly for multicomponent oxides; (2) non-equilibrium growth enabling metastable or amorphous phases with favorable ion transport; and (3) tunable processing environments that allow reactive gas incorporation. PLD has been widely applied to cathodes such as LMO [69], LCO [70], Li_2_MnO_3_ [71], LiFePO_4_ [72], MoO_3_ [73], LTO [74], and SSEs such as LiPON [75–77], LLZO [78–81], and LATP [82]. Deposition parameters (e.g., laser fluence, substrate temperature, pressure) strongly influence crystallinity, microstructure, and ionic transport.

Typically, PLD yields highly crystalline thin films, where ordered lattices lower migration barriers and improve ionic conductivity [38, 83]. However, excessive crystallinity may induce interfacial stress, crack formation, or grain boundary degradation, undermining long-term stability. Conversely, under tailored conditions, PLD can also yield amorphous or metastable phases with isotropic Li^+^ transport and enhanced interface conformity. Additionally, PLD enables heterostructures and functionally graded films that reconcile high conductivity with interfacial robustness [84]. Nevertheless, scalability challenges remain: particulate formation during ablation may compromise uniformity, deposition area is limited by laser spot size, and high capital cost restricts industrial adoption. While PLD is invaluable for materials discovery and thin-film prototyping, its translation into commercial TFMB manufacturing requires breakthroughs in uniformity, throughput, and integration strategies.

#### Thermal Evaporation and E-beam Evaporation

Evaporation-based PVD methods, among the earliest thin-film deposition techniques, remain attractive for their simplicity and high deposition rates. In thermal evaporation, source material is resistively heated within a crucible until vaporized and condensed on the substrate (Fig. 1c). This approach has been extensively used for metallic lithium films with micron-scale thickness [40, 85, 86]. However, film uniformity and step coverage are limited, especially for complex geometries. Electron-beam evaporation (EBE) improves control by using a focused electron beam to heat high-melting-point materials (Fig. 1d), enabling deposition of Si, SiC [87], SnO_2_ [88, 89], Li–Zn alloys [90], and amorphous LLTO [91]. EBE achieves deposition rates of 0.1–100 μm min^–1^, far exceeding those of MS and PLD [92]. Despite their efficiency, both methods are line-of-sight processes with limited conformality, but their scalability and simplicity make them well-suited for specific TFMB components, especially Li anodes and buffer layers.

To overcome the intrinsic limitations of conventional PVD, various hybrid and assisted vapor deposition strategies have been developed to improve film uniformity, interfacial control, and process flexibility. PVD combined with CVD integrates the high purity of physical deposition with the conformality of chemical processes, enabling uniform coatings on complex substrates [93]. ALD-assisted PVD allows atomic-level modulation of interfacial composition, such as controlled Li^+^ concentration gradients, to mitigate space-charge effects and reduce interfacial impedance [94]. Plasma-enhanced PVD facilitates surface reactions and film densification at reduced temperatures, which is advantageous for flexible or polymer-supported systems [36]. Recent advances include high power impulse magnetron sputtering (HiPIMS) for denser and more crystalline films [95], ion beam assisted deposition (IBAD) for controlling surface energy and texture [96], and hybrid sputtering combined with sol–gel or spin-coating for scalable composite interfaces [97]. Reactive e-beam evaporation also enables low-temperature synthesis of high purity oxide thin films compatible with flexible substrates [98]. Overall, these hybrid and assisted approaches integrate the merits of physical and chemical vapor deposition, providing a versatile and scalable platform for producing dense, uniform, and compositionally tailored thin films, thereby extending the applicability of PVD to multifunctional and flexible TFMB architectures.

### Chemical Vapor Deposition (CVD) and Its Derivatives

As TFMBs gain prominence, demands for high-quality films with structural tunability and nanoscale uniformity have intensified. Beyond PVD, CVD and its derivatives offer conformal deposition, 3D compatibility, and precise interface engineering. In CVD, gaseous precursors react or decompose on heated substrates to form continuous films (Fig. 1e). Variants include metal–organic CVD (MOCVD), plasma-enhanced CVD (PECVD), and laser-assisted CVD (LCVD). Notably, PECVD enables low-temperature deposition, making it suitable for flexible and polymer-based devices.

#### Conventional CVD

Conventional CVD provides films with high phase purity, uniformity, and composition control, alongside excellent step coverage on complex substrates. It has been widely adopted for TFMB cathodes and SSEs, including LCO [99], LiPON [100, 101], and LTO [102]. Beyond planar films, CVD enables structured electrodes such as LTO pillar arrays [103], which increase surface area and kinetics. Compared with PVD, CVD offers higher material utilization and better scalability [104], although precursor cost, safety, and chemical control present challenges.

CVD, as a high-temperature process, often yields crystalline films with fast Li^+^ diffusion due to reduced migration barriers [105]. While this benefits rate performance, crystallinity can also lead to stress accumulation and grain boundary degradation. In addition, porous or nanoparticulate morphologies common in CVD films enlarge active areas but may accelerate side reactions.

#### Atomic Layer Deposition (ALD)

ALD, a subclass of CVD, relies on self-limiting surface reactions to achieve angstrom-level thickness control and exceptional conformality (Fig. 1f). Its capability to coat deep trenches, nanopores, and 3D scaffolds makes it highly attractive for advanced TFMBs. ALD has been utilized for LiPON electrolytes [106, 107], LTO anodes [108], ultrathin Al_2_O_3_ coating [109], and protective interfacial layers, which enhance stability and suppress side reactions. Its deterministic growth and reproducibility enable precise interface modulation to optimize ionic conductivity and battery lifespan. However, ALD’s low throughput and high cost limit its large-scale adoption. Currently, it is primarily applied to performance-critical regions such as interfaces or ultrathin coatings. Nevertheless, its evolving role in high-fidelity nanostructuring underscores ALD as a key enabling technology for next-generation TFMBs. Table 2 summarizes correlations between deposition methods, film crystallinity, microstructure, and ionic conductivity, complementing Fig. 1 and offering a concise framework for material and process selection.

**Table 2:** Comparison of vapor deposition techniques in terms of microstructure and electrochemical properties

Technique	Crystallinity	Microstructural features	Ionic transport characteristics	Electrochemical implications
MS	Amorphous at RT, tunable by annealing	Dense, smooth, uniform	Isotropic Li^+^ diffusion, conductivity lower than crystalline films	Superior interface stability and cycle life, but limited rate performance
PLD	Highly crystalline, stoichiometry preserved	Dense films, heterostructure accessible	High conductivity, anisotropic transport	Excellent rate performance, stability depends on microstructure
CVD	High crystallinity, tunable by temperature/precursor	Porous/nanoparticle-like, large surface area	Ordered lattice enables high mobility, possible anisotropy	Enhanced rate due to abundant sites, prone to interfacial instability
ALD	Exceptional uniformity, sub-nm control	Conformal ultrathin interfacial layers	Conductivity tunable by defect/stress engineering	Effective for interface engineering, reduces impedance and enhances stability
Thermal evaporation	Poorly crystalline or amorphous	Porous, columnar, low density	Limited ionic conduction	Primarily suited for metallic current collectors
E-beam evaporation	Amorphous/low crystallinity	Moderately dense, columnar growth	Restricted ionic conduction	Mostly for metals/simple oxides, limited for complex TFMB systems

## Vapor Deposition for TFMBs Fabrication and Integration

### Architectural Framework and Design Strategies for TFMBs

TFMBs are generally assembled in a multilayer planar architecture comprising a current collector, cathode, SSE, anode, and encapsulation layer (Table 3). The total thickness is typically below 100 μm, with individual layers ranging from tens of nanometers to several micrometers. This compact configuration enables seamless integration with microelectronic systems, allowing efficient use of limited volumetric space and delivering high energy density in spatially constrained environments. Consequently, TFMBs are particularly suited for next-generation portable, wearable, and implantable electronics.

**Table 3:** Representative functional materials and their targeted roles in TFMBs

Component	Key requirements	Representative materials
Cathode	High capacity, electronic conductivity, chemical compatibility with electrolyte	Layered oxides (LiCoO_2_, LiMn_2_O_4_, LiNiO_2_), polyanionic compounds (LiFePO_4_), transition metal oxides (V_2_O_5_, MoO_3_, WO_3_)
Anode	Adequate capacity, electrical conductivity, minimal volume change, stable SEI/electrolyte interface	Li Metal, intercalation-type (Li_4_Ti_5_O_12_, TiO_2_, Nb_2_O_5_), alloy-type (Si, Sn, In, Mg), conversion-type (SnO_2_, Si_3_N_4_, SiC)
Solid electrolyte	High ionic conductivity, electronic insulation, thermal/electrochemical stability, dense microstructure	LiPON, garnet (LLZO), perovskite (LLTO), antiperovskite (Li_3_OX), NASICON (LATP)
Interface engineering	Strong adhesion, electrochemical stability, suppression of side reactions	Tailored interlayers depending on electrode/electrolyte combinations
Current collector	High electronic conductivity, chemical/mechanical stability, lightweight	Pt, Au, Ag, Cu, Al, Ti, Ni

Each layer fulfills distinct electrochemical and structural roles. Current collectors require high conductivity, electrochemical stability, and mechanical robustness. Noble metals (Au, Pt) provide excellent properties but are cost-prohibitive. Thus, bilayer metallization stacks (e.g., Ti/Pt, Cr/Pt) are typically used on the cathode side to compensate for the limited conductivity of oxide cathodes. By contrast, the anode current collector can adopt less stringent compositions due to the intrinsically high conductivity of Li and its alloys. The cathode layer must balance capacity and transport kinetics, with thickness usually below 50 μm to mitigate Li^+^ diffusion constraints. LCO and LMO remain the most widely adopted cathodes, offering high capacity, voltage stability, and cycling durability. The SSE layer, typically 2–3 μm thick, must ensure high ionic conductivity (10^−7^–10^−4^ S cm^−1^), negligible electronic transport, and robust chemical/mechanical compatibility with both electrodes. Among candidate electrolytes, LiPON and its doped derivatives are the most mature, combining stable interfaces with facile thin-film processability. The anode, often Li metal, Li alloys, or LTO, must enable efficient (de)lithiation while maintaining chemical and interfacial stability with the SSE. Its thickness is generally tailored to match the cathode, preventing imbalance and lithium plating.

Beyond intrinsic material properties, interfacial engineering critically governs ionic mobility, mechanical integrity, and cycling stability. Interfaces should facilitate fast Li^+^ transport, suppress interdiffusion and parasitic reactions, and ensure strong adhesion. These objectives are often achieved via passivation layers, interfacial interlayers, or compositional gradients. Importantly, the entire device stack is commonly fabricated by vacuum-based vapor deposition techniques, especially RF/DC magnetron sputtering, enabling precise control of morphology, stoichiometry, and nanoscale uniformity. Nevertheless, deposition parameters must be carefully optimized for each material. For example, LCO requires oxygen-rich conditions and post-annealing to stabilize its layered phase, whereas LiPON properties are highly sensitive to nitrogen flow and RF power. Furthermore, thermal and mechanical mismatches across adjacent layers can lead to stress accumulation, cracking, or delamination, compromising device reliability. Therefore, co-optimization of materials selection, deposition protocols, and interfacial design is indispensable for high-performance TFMBs.

**Fig. 2:**
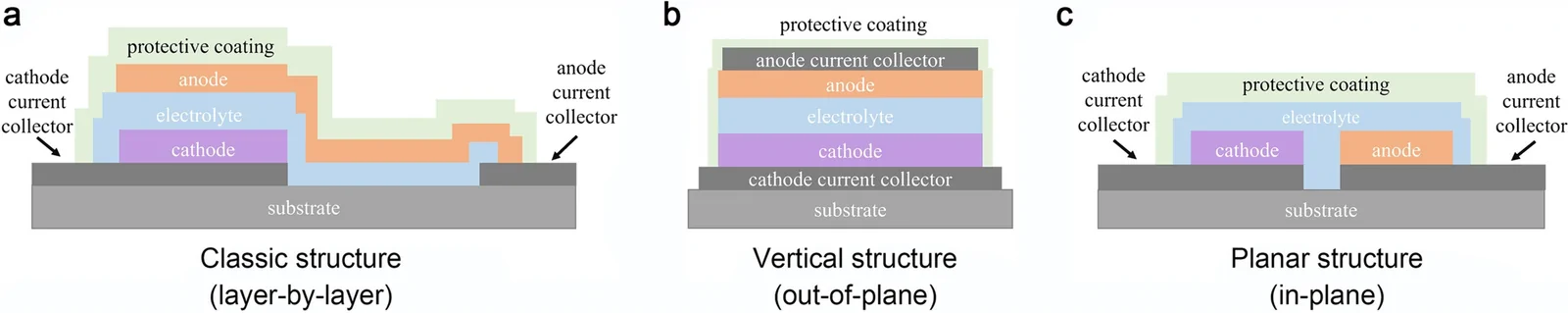
Schematic illustration of typical structural configurations of TFMB. **a** Classic stacked architecture with sequentially deposited multilayers to maximize volumetric energy density. **b** Vertically integrated structure that enlarges interfacial area, thereby enhancing areal capacity and rate performance. **c** Planar configuration with laterally arranged electrodes, offering simplified fabrication and straightforward on-chip integration

From a structural viewpoint, all-solid-state TFMBs can be categorized into three representative architectures: classic layer-by-layer, vertical (out-of-plane), and planar (in-plane) configurations (Fig. 2). The classic layer-by-layer design, pioneered by Bates et al. [110], relies on the sequential deposition of cathode, electrolyte, and anode layers on a flat substrate, followed by encapsulation. This architecture demonstrates outstanding compatibility with standard microfabrication processes, high device yield, and excellent scalability, making it the most widely adopted configuration in early TFMB demonstrations. Nevertheless, its reliance on high-resolution lithographic patterning and stringent control over layer thickness and alignment imposes complexity, while the limited electrode–electrolyte interfacial area constrains both power density and rate capability. In contrast, vertical architectures, as demonstrated by Nakazawa et al. [111], leverage columnar stacking of functional layers with uniform thickness, thereby increasing electrode–electrolyte interfacial contact and improving volumetric energy density. Such designs are particularly promising for applications requiring high areal energy within compact footprints. However, these benefits come at the expense of fabrication complexity: stringent requirements for film conformity, step coverage, and multilayer alignment increase the risk of short circuits and impose substantial challenges for scalable packaging and integration. To mitigate these limitations, planar architectures have emerged as an attractive alternative. In these systems, cathode, electrolyte, and anode are laterally patterned on the substrate surface, which simplifies device encapsulation and enhances mechanical robustness. The reduced stacking complexity also enables higher design flexibility, allowing tailored geometries for unconventional substrates, flexible electronics, and even curvilinear systems. Such architectures are particularly suited for MEMS, biomedical implants, and wearable platforms where device form factor, mechanical adaptability, and reliability are prioritized over maximum volumetric density. In addition, advances in lithographic techniques, inkjet patterning, and spatially selective deposition have significantly expanded the manufacturability of in-plane designs, suggesting strong potential for large-area integration and hybrid multifunctionality.

Collectively, the evolution from layer-by-layer to vertical and planar configurations highlights the critical role of structural engineering in balancing scalability, energy density, mechanical reliability, and integration capability, thereby laying the foundation for next-generation TFMBs tailored to diverse microelectronic ecosystems.

### Cathode Materials and Structural Optimization for TFMBs

**Fig. 3:**
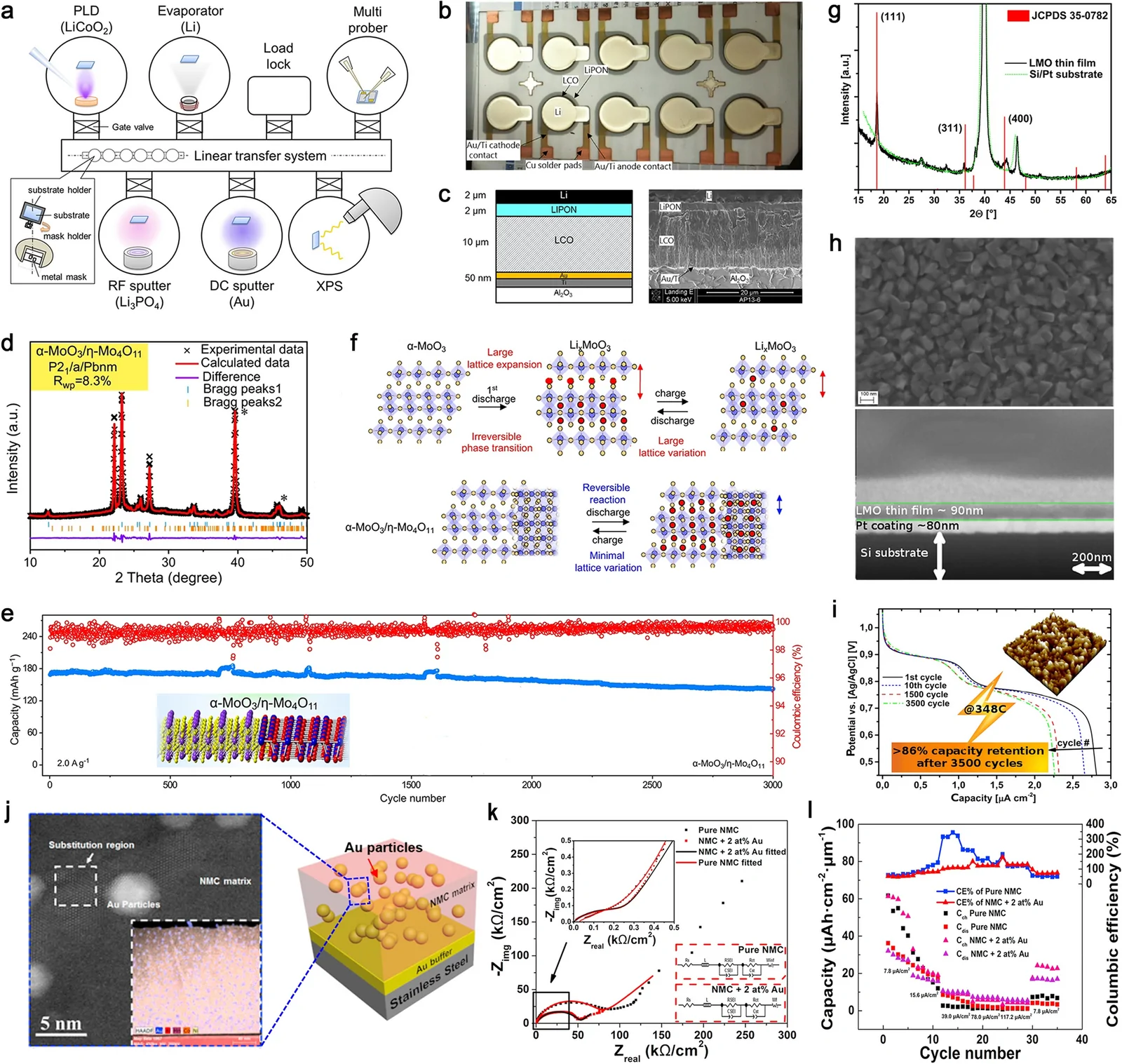
Comprehensive characterization of TFMB fabrication platforms, structural configurations, and electrochemical performance. **a** Schematic of an all-in-vacuum fabrication and in situ evaluation system for TFMBs. Reproduced with permission from Ref. [112] Copyright 2016 Elsevier Publishing. **b** Top-view optical image of a single substrate containing ten integrated TFMB units. **c** Schematic and cross-sectional SEM image of a stacked TFMB. Reproduced with permission from Ref. [113] Copyright 2017 Elsevier Publishing. **d** Rietveld-refined XRD patterns of α-MoO_3_/η-Mo_4_O_11_ films. **e** Long-term cycling performance of α-MoO_3_/η-Mo_4_O_11_ electrode.** f** Mechanism of structural stabilization via lattice pinning in α-MoO_3_ heterostructures. Reproduced with permission from Ref. [115] Copyright 2023 Springer Nature Publishing. **g** XRD pattern of LMO thin film deposited via PLD using a multilayer target on a Pt-coated Si substrate. **h** Top-view SEM and cross-sectional FIB-SEM images of the LMO thin film. **i** Discharge profiles of LMO-based TFMBs during galvanostatic cycling at high current density. Reproduced with permission from Ref. [116] Copyright 2017 ACS Publishing. **j** HAADF image of Au-doped NMC thin film with elemental mapping. **k** Rate performance of Au-modified NMC films. **l** Cycling performance comparison of pristine and Au-modified NMC films. Reproduced with permission from Ref. [117] Copyright 2018 Elsevier Publishing

Cathode materials constitute a decisive component of TFMBs, directly governing energy density, rate capability, and long-term cycling performance. Various chemistries with layered, spinel, and olivine-type crystal structures have been extensively explored, each exhibiting distinct Li^+^ transport kinetics, redox mechanisms, and structural evolution during cycling. To enable systematic investigation under well-controlled environments, Hitosugi et al. [112] developed a fully vacuum-integrated platform that encompasses thin-film deposition, device assembly, and electrochemical testing without ambient exposure (Fig. 3a). This approach effectively suppresses contamination and parasitic reactions, thereby preserving the integrity of electrolyte–electrode interfaces. Using this system, LCO films deposited by PLD at 400 °C on annealed Au layers exhibited dominant (0001) reflections, indicating strong c-axis orientation and high crystallinity. Electrochemical measurements revealed outstanding reversibility and minimal hysteresis, with excellent rate performance (74% capacity retention at 20 C), underscoring the critical importance of clean and well-defined interfaces.

For energy density optimization, Anapolsky et al. [113] systematically investigated morphology and orientation control in LCO films with thicknesses up to 10 μm (Fig. 3c). They demonstrated that thick-film cathodes require well-preserved crystallographic orientation to maintain > 80% charge utilization and long-term cycling stability. Specifically, a reduced oxygen partial pressure during sputtering (e.g., 4% O_2_ in Ar) suppressed the (003) peak even after annealing, indicating the loss of c-axis orientation. By precisely controlling the texture, a 10 μm-thick LCO film delivered 600 μAh cm^−2^ at 0.1 C and > 95% retention after 100 cycles at 0.2 C (Fig. 3b). In contrast, orientation-deficient films exhibited similar initial capacities but severe rate degradation and cycling failure, highlighting the necessity of texture engineering for thick-film cathodes. Kim et al. [114] further advanced this field by depositing LCO films via MS directly onto NASICON-type solid electrolytes. Post-annealing at 500 °C induced interfacial crystallization and bonding, substantially reducing interfacial resistance and promoting Li^+^ transport, thereby offering a viable strategy to integrate oxide cathodes with solid electrolytes in TFMBs. Interfacial degradation caused by structural instability remains a key bottleneck in cathode cycling. To address this, Xia et al. [115] proposed an epitaxial interfacial pinning strategy for layered oxides. By tuning O_2_/Ar flow ratios during MS, they fabricated a coherent heterostructure comprising α-MoO_3_ and η-Mo_4_O_11_ (Fig. 3d). The η-Mo_4_O_11_ phase acted as a structural anchor, reducing interlayer expansion from 16 to 2% (Fig. 3f). The resulting TFMB exhibited 67 mAh g^−1^ after 3000 cycles at 2 A g^−1^, retaining ~ 74% capacity (Fig. 3e). This illustrates the efficacy of epitaxial interface design in suppressing lattice strain and capacity fade. Spinel-structured LMO has also shown excellent applicability in aqueous-compatible TFMBs. Fehse et al. [116] employed a multilayer PLD strategy to deposit LMO films on Pt-coated Si substrates (Fig. 3g–h). These cathodes exhibited pseudocapacitive response and superior rate capability, delivering 2.6 μAh cm^−2^ at 348 C and demonstrating remarkable durability over 3500 cycles with a per-cycle retention of 99.996% (Fig. 3i). These results highlight the kinetic advantages of spinel cathodes for high-power microdevices.

More recently, ternary layered oxides (e.g., Ni–Co–Mn series, NMC) have attracted increasing attention. Wang et al. [117] fabricated highly oriented Li(Ni_0.5_Mn_0.3_Co_0.2_)O_2_ (NMC532) thin films by PLD, where Au nanoparticle incorporation reduced interfacial charge-transfer resistance and enhanced capacity, coulombic efficiency, cycling stability, and rate performance (Fig. 3j–l). Deng et al. [118] further revealed that annealing temperature, composition, and thickness critically influence Li^+^ diffusion kinetics in LiNi_1/3_Co_1/3_Mn_1/3_O_2_ films, with optimal samples annealed at 450 °C showing high capacity at low rates and stable cycling at higher rates. Similarly, Jacob et al. [119] reported highly oriented NMC532 films deposited on stainless steel substrates, achieving excellent densification and electrochemical performance. Collectively, these studies demonstrate that deposition parameters, annealing protocols, and microstructural features are decisive for unlocking the potential of NMC thin-film cathodes in TFMBs.

Olivine-type LiFePO_4_ (LFP), characterized by a stable ~ 3.4 V discharge plateau and a theoretical capacity of 170 mAh g^−1^, is another promising candidate owing to its abundance and safety [120–122]. However, its poor electronic conductivity (~ 10^−8^–10^−10^ S cm^−1^), low Li^+^ diffusion coefficient (~ 2.7 × 10^−9^ cm^2^ s^−1^), and relatively high activation energy (~ 0.73 eV) restrict its rate performance [123]. To overcome these challenges, film thinning and nanostructuring have been pursued. Hirayama et al. [124] prepared vertically aligned LFP nanopillar films on structured Pt–Ti–Si substrates by thermally inducing nanoscale surface protrusions during photoresist decomposition (> 400 °C). A 60 nm-thick nanopillar film delivered 360 mAh g^−1^ at 1 C, showcasing the advantages of directed nanoarchitectures in facilitating Li^+^ transport. Composition tuning has also been explored. Choi et al. [125] fabricated highly transparent LiFe_1–x_Mn_x_PO_4_ films via co-sputtering on ITO-coated glass. By adjusting the target–substrate distance, they modulated Mn content, with LiFe_0.77_Mn_0.23_PO_4_ delivering the best performance—45.7 μAh cm^−2^ μm^−1^ due to dual Fe^3+^/Fe^2+^ (3.4 V) and Mn^3+^/Mn^2+^ (4.1 V) redox activity. Mn doping enhanced capacity without requiring conductive additives, though excess Mn impaired ionic transport. Notably, these films maintained > 80% transmittance, suggesting potential in transparent and optoelectronic microsystems. Thus, strategic control of crystallographic orientation, interface coherence, nanostructuring, and compositional modulation has enabled cathodes in TFMBs to evolve into multifunctional platforms with enhanced energy density, cycling stability, and even optical transparency.

The electrochemical behavior of LCO thin films is strongly governed by crystallinity and orientation, both sensitive to deposition temperature [126]. At submicron thickness (< 0.5 µm), surface energy promotes preferential (003) orientation at low temperatures, whereas increased thickness (> 1 µm) and high deposition temperatures (~ 400 °C) favor orientations such as (101), (104), or (110) due to strain effects [127]. Moderate heating improves crystallinity and grain size, enabling LCO thin films to achieve 64 µAh cm^−2^ µm^−1^, comparable to bulk LIBs [128]. However, deposition or annealing above 500 °C, while beneficial for crystallinity, is incompatible with microfabrication. Low-temperature RF sputtering (≤ 300 °C) yields oriented LCO films but often results in poor crystallinity and rapid fading [129]. For LMO thin films, deposition temperature dictates growth mode and morphology. At 300 °C, limited adatom mobility induces Volmer–Weber growth, forming nanosheet-based island structures [130]. Raising deposition temperature to 600 °C followed by annealing at 700 °C produces highly crystalline 3D nanowall arrays, which enlarge electrolyte contact area, shorten Li^+^ diffusion pathways, and mitigate interfacial disorder. Compared to planar films, these 3D nanostructures markedly reduce interfacial resistance and enhance rate capability in LMO/LiPON/Li cells.

In brief, LCO benefits from high-temperature processing that enhances crystallinity and capacity but faces integration challenges due to thermal incompatibility, highlighting the need for optimized low-temperature strategies. By contrast, LMO enables high-performance 3D nanostructures at moderate processing conditions (~ 300 °C), offering cost-effectiveness and environmental advantages. Remaining challenges lie in tailoring deposition protocols for thick-film integration. A systematic comparison of LCO and LMO thin films under different processing conditions is summarized in Table 4.

**Table 4:** Comparative of deposition conditions and electrochemical behavior of LCO and LMO thin-films

Material	Deposition conditions	Atmosphere	Preferred Orientation/Morphology	Crystallinity & electrochemical behavior	Key limitations
LCO [53, 129, 183]	10^−2^–10^−3^ Torr (MS/PLD), RT–500 °C	O_2_ or O_2_/Ar	RT–300 °C: (003) orientation, ≥ 400 °C: (101)/(104)/(110), > 500 °C: best crystallinity	High crystallinity above 500 °C, capacity ≈ 64 μAh‧cm^−2^‧μm^−1^ (~ 140 mAh‧g^−1^), stable cycling	> 500 °C incompatible with CMOS, low-temperature films show poor crystallinity and rapid fading
LMO [130]	10^−2^–10^−1^ Torr (MS), 300–500 °C deposition, annealing up to 700 °C	O_2_ atmosphere	3D nanosheets or nanowall arrays (Volmer–Weber growth)	Enlarged interface area, shortened diffusion paths, improved rate performance	Thick-film fabrication difficult, precise temperature control needed to avoid phase instability and Mn valence shifts

### Anode Materials and Interfacial Engineering for TFMBs

The choice of anode material critically influences both the energy density and cycling durability of TFMBs. Among the available candidates, Li metal remains the most attractive due to its ultrahigh theoretical specific capacity (3861 mAh g^−1^) and lowest redox potential (− 3.04 V vs. H^+^), which enable maximization of cell voltage and gravimetric energy density. Owing to its relatively low melting point (180.5 °C), thermal evaporation has been widely adopted for depositing Li thin films in TFMB architectures. Benefiting from the lithium reservoir function of the cathode, the Li layer is typically fabricated with submicron thickness, thereby reducing material consumption. However, poor intrinsic wettability between Li and most ceramic SSEs severely hinders interfacial contact and ion transport. To overcome this issue, Luo et al. [131] proposed a surface energy modulation approach by incorporating carbon nanoparticles into molten Li. This modification reduced the interfacial energy with SSEs or Cu substrates, leading to improved wettability and uniform Li deposition. The versatility of this strategy was demonstrated across both garnet-type SSEs and metallic current collectors, highlighting its potential for broader solid-state integration. Beyond Li metal, alternative anodes have been explored to address interfacial instability and dendrite growth. Alloy-type (e.g., Si–Ge [132], Si–Cu [133]) and intercalation-type (e.g., TiO_2_ [134], LTO [135–137]) systems represent the most investigated options. Among alloy-based anodes, silicon exhibits the highest theoretical capacity (~ 4200 mAh g^−1^, Li_4.4_Si). Yet, its enormous volume change (~ 420%) during lithiation leads to pulverization, poor mechanical integrity, and rapid capacity fading [138]. Nanostructuring has emerged as a promising mitigation strategy. For instance, thin Si films of 250 nm and 1000 nm were compared [139], where the thinner film exhibited improved initial performance but suffered severe cracking after 30 cycles due to stress-induced failure. The jagged morphology of as-deposited Si layers further exacerbated localized stress concentration and delamination, underscoring the importance of stress management in high-capacity thin-film anodes.

**Fig. 4:**
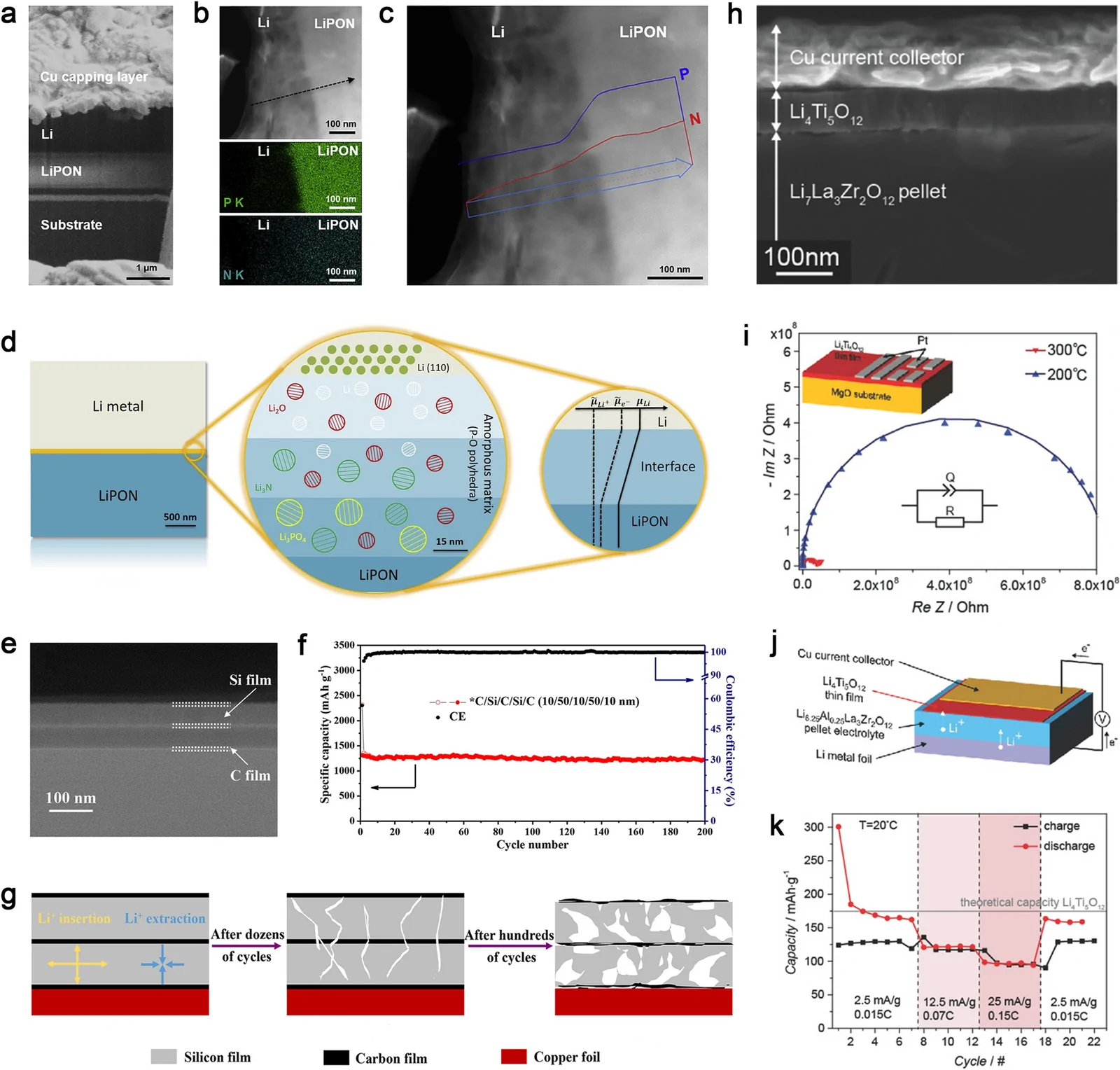
Characterization of interfacial structures and failure mechanisms in TFMB electrodes and electrolytes. **a** Cryo-FIB-SEM cross-sectional image of the Li/LiPON interface. **b** Cryo-STEM dark-field image of the Li/LiPON interface with corresponding elemental distribution. **c** EDS line scan of P and N signals along the dashed line in b, showing interfacial composition. **d** Schematic illustration of the multilayered interphase formed at the Li/LiPON interface. Reproduced with permission from Ref. [140] Copyright 2020 Elsevier Publishing. **e** SEM image of a multilayered C/Si/C/Si/C thin-film electrode with alternating layers. **f** Cycling performance of the multilayered Si/C thin-film electrode, indicating improved reversibility. **g** Schematic of failure mechanisms in multilayered Si/C thin-film anodes. Reproduced with permission from Ref. [143] Copyright 2019 Elsevier Publishing. **h** Cross-sectional SEM image of an LTO thin film deposited on a solid electrolyte substrate via PLD. **i** Nyquist plots of in-plane impedance measured with Pt contacts at 200 °C and 300 °C. **j** Schematic configuration of a half-cell with Li foil/Li_6.25_Al_0.25_La_3_Zr_2_O_12_ pellet/LTO thin film/Cu current collector. **k** Rate capability of the half-cell under varying current densities, showing discharge capacity exceeding charge capacity. Reproduced with permission from Ref. [144] Copyright 2018 Wiley Publishing

The high sensitivity of Li/LiPON interfaces to ambient atmosphere and electron beam exposure complicates structural characterization. Meng et al. [140] addressed this challenge using cryogenic TEM (cryo-TEM) combined with cryo-FIB preparation. Li was thermally evaporated onto a LiPON SSE, and analysis revealed an 80 nm-wide interfacial zone containing elemental gradients of N and P, phase-segregated products, and amorphous-layered domains (Fig. 4a–d). These findings provide mechanistic insights into interphase formation and highlight the importance of interface engineering in TFMBs. In addition to elemental and alloy systems, semiconducting anodes have also been investigated. Guo et al. [141] introduced Cu_2_ZnSnS_4_ (CZTS), a well-known photovoltaic material, into microfabricated TFMBs. The LCO/CZTS full cell with an active area of 0.52 mm^2^ delivered superior electrochemical performance compared with LCO/SnO_2_ counterparts, retaining 4.2 nAh after 20 cycles and exhibiting higher coulombic efficiency. The improved reversibility and compatibility highlight CZTS as a promising multifunctional anode for miniaturized storage systems. The compositional tuning of anodes also plays a decisive role. Stewart et al. [142] investigated nitrogen-doped tin oxynitride (SnO_x_N_y_) films via ALD. Nitrogen-rich SnO_x_N_y_ displayed higher capacities, lower discharge voltages, and enhanced cycling stability due to facilitated Li^+^ transport and suppressed resistive phase formation, while trace oxygen showed negligible adverse effects. In order to alleviate stress-induced degradation, multilayer configurations have been employed. Yang et al. [143] fabricated binder-free multilayer Si/C thin films by MS, resulting in a C/Si/C/Si/C architecture (Fig. 4e). The electrode delivered an initial discharge capacity of 2316 mAh g^−1^ and retained 1220.9 mAh g^−1^ after 200 cycles at 0.2 C, corresponding to 90.1% capacity retention (Fig. 4f). Carbon layers acted as elastic buffers to accommodate Si expansion and facilitated stable SEI formation (Fig. 4g). Moreover, moderate deposition temperatures (100–300 °C) further improved film densification and electrochemical stability.

Low-voltage intercalation anodes like LTO also show promise due to their structural stability and negligible volume change. Pfenninger et al. [144] deposited LTO thin films onto Al-doped LLZO SSEs using PLD (Fig. 4h–j). The resulting interface demonstrated excellent mechanical and electrochemical compatibility, delivering capacities close to the theoretical value of 175 mAh g^−1^ (Fig. 4k). Stable cycling (90% retention over 22 cycles at 2.5 mA g^−1^) and robust rate capability further highlight the potential of oxide-based intercalation anodes in suppressing dendrites and enhancing safety in microbattery system. In a word, rational anode design, ranging from elemental and alloy to conversion and intercalation types, combined with advanced interface characterization and multilayer engineering, is essential for achieving stable and high-performance TFMBs. Future advances will rely on strategies that ensure interfacial compatibility, stress mitigation, and conformal film growth to enable scalable integration into solid-state microbattery.

### Solid-State Electrolytes: Design Criteria, Functionalization, and Thin-Film Engineering

Solid-state electrolytes (SSEs) serve as the ionic conductor and electronic insulator in TFMBs, determining electrochemical performance, interfacial stability, and cycling longevity. An ideal SSE must satisfy three stringent requirements: (1) high Li^+^ conductivity (> 10^−5^ S cm^−1^) for efficient ion transport; (2) ultralow electronic conductivity (< 10^−9^ S cm^−1^) to suppress leakage and self-discharge; and (3) high mechanical modulus and hardness to resist dendritic penetration and maintain film integrity. In addition, SSEs for TFMBs must be compatible with thin-film fabrication, requiring low-temperature processability to avoid electrode degradation and the ability to form dense, pinhole-free, smooth films with minimal grain boundaries. Structurally, SSEs can be classified into amorphous and crystalline phases. Amorphous SSEs, though showing lower conductivity, provide isotropic transport, absence of grain boundaries, and excellent film formability, while crystalline SSEs generally possess higher conductivities but suffer from grain-boundary impedance and interfacial instabilities.

#### LiPON and Derivatives

**Fig. 5:**
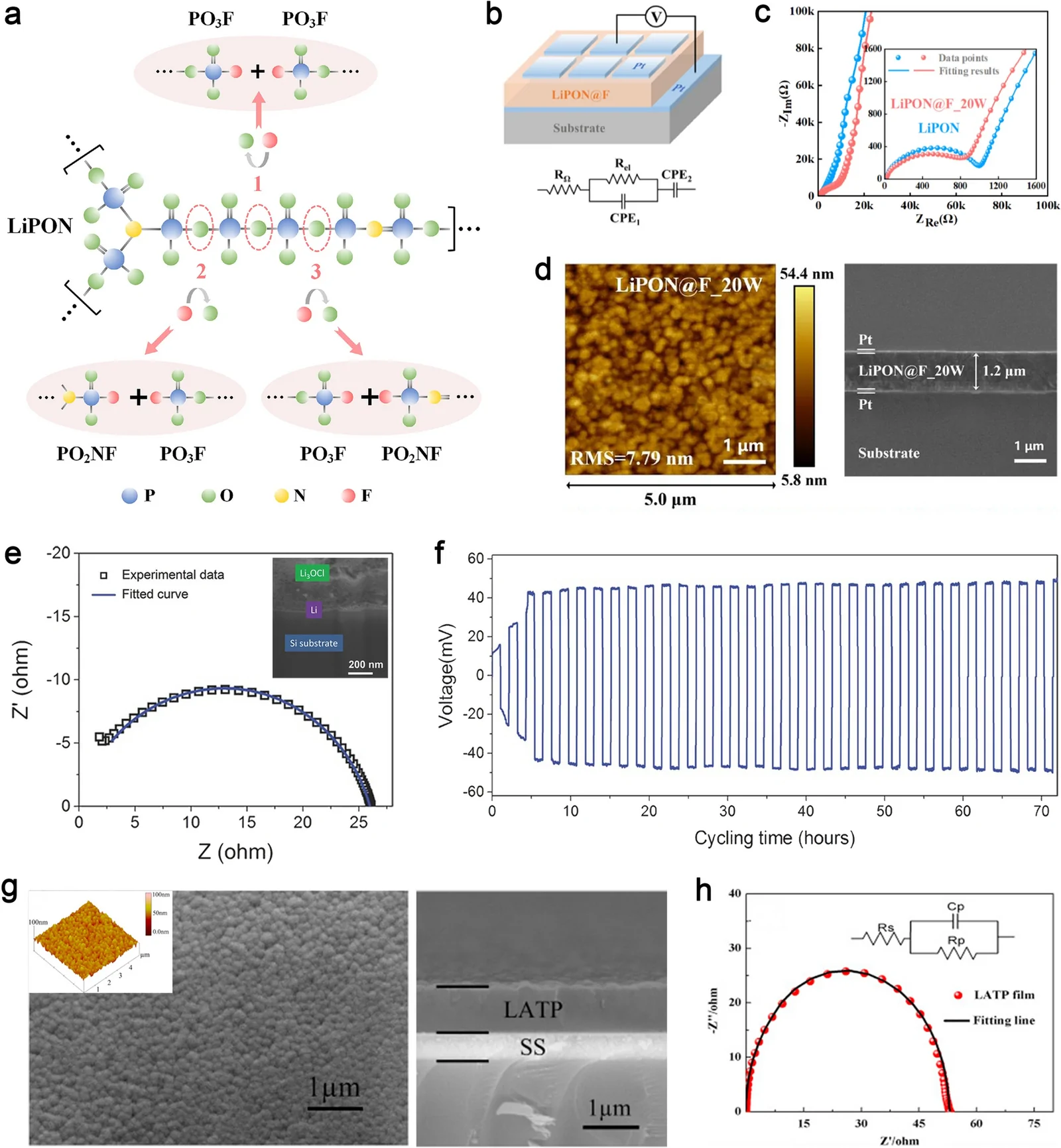
Structural design, morphology, and electrochemical properties of advanced thin-film solid electrolytes for TFMBs. **a** Schematic illustration of the fluorine-doped LiPON structure. **b** Experimental setup for electrochemical testing of LiPON@F films. **c** Room-temperature Nyquist plots of LiPON@F samples with inset showing the magnified high-frequency region. **d** AFM image and cross-sectional SEM morphology of the as-deposited LiPON@F thin films. Reproduced with permission from Ref. [146] Copyright 2023 Elsevier Publishing. **e** Nyquist plot and fitted impedance curves of a Li_3_OCl-based solid electrolyte at room temperature, with inset cross-sectional SEM image of the interfacial structure. **f** Cycling performance of a Li/Li_3_OCl/Li symmetric cell. Reproduced with permission from Ref. [147] Copyright 2016 Wiley Publishing. **g** SEM images of the LATP thin-film surface with inset AFM image, alongside cross-sectional morphology. **h** Complex impedance spectra of the LATP thin-film electrolyte. Reproduced with permission from Ref. [148] Copyright 2016 Elsevier Publishing

LiPON remains the most widely employed SSE owing to its exceptional chemical stability, wide electrochemical window (5.5 V vs. Li^+^/Li), and ultralow electronic conductivity (10^−14^ S cm^−1^). However, its moderate ionic conductivity (10^−6^ S cm^−1^) limits rate capability. To address this, doping strategies have been pursued. Huang et al. [145] incorporated Mn into LiPON via MS, forming Mn–O–P linkages that enhanced Li^+^ mobility and reduced surface roughness, thereby doubling the ionic conductivity to 5.0 × 10^−6^ S cm^−1^. Subsequently, fluorine plasma modification yielded LiPON@F films with enhanced conductivity (1.0 × 10^−6^ S cm^−1^), lower activation energy (0.39 eV), and wider stability window (4.2 vs. 3.9 V for pristine LiPON) (Fig. 5a–d) [146]. LiPON@F also exhibited superior humidity resistance, retaining 85.5% conductivity after 120 h at 50% RH, compared to 64.6% for pristine LiPON. These improvements were attributed to defect passivation and phosphate network modulation.

#### Emerging Crystalline SSEs

**Fig. 6:**
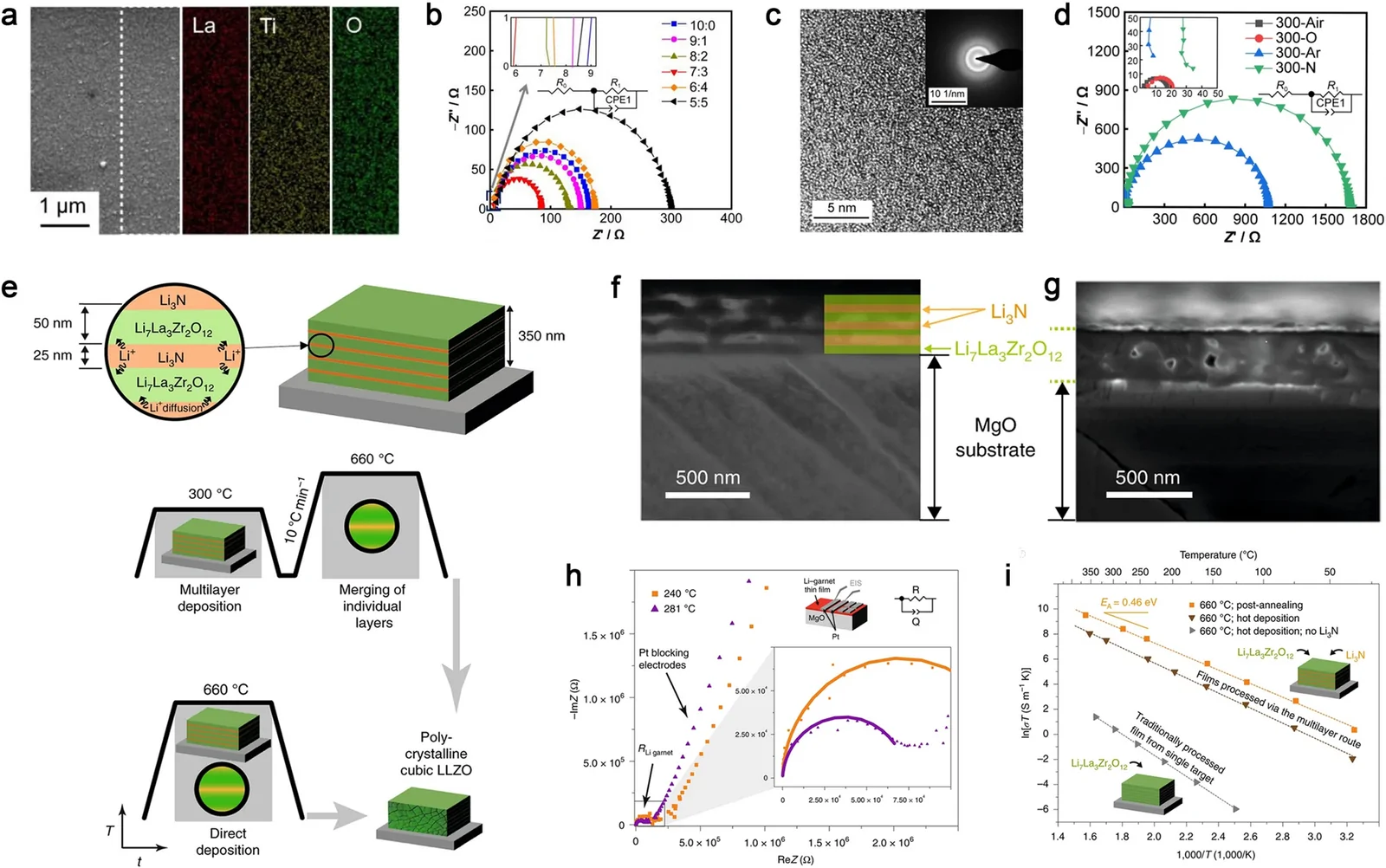
Morphology, structural features, and ionic conductivity of perovskite- and garnet-type thin-film solid electrolytes for TFMBs. **a** Surface SEM image and elemental mapping of amorphous LLTO thin films deposited under an O_2_:Ar ratio of 3:7. **b** Electrochemical impedance spectra of LLTO thin films prepared under different O_2_ partial pressures. **c** Selected-area electron diffraction (SAED) patterns of LLTO thin films after annealing at 300 °C. **d** Electrochemical impedance spectra of LLTO films annealed in different gas atmospheres. Reproduced with permission from Ref. [149] Copyright 2022 Science Press and Springer Publishing. **e** Schematic of a multilayer deposition strategy for Al-doped LLZO thin films fabricated via PLD. **f** Cross-sectional SEM image of a multilayer stack (delithiated Al-LLZO/Li_3_N) deposited at 300 °C on a MgO substrate. **g** SEM image of the post-annealed thin-film structure at 660 °C, showing a consolidated ~ 330 nm film. **h** Nyquist plots of the multilayered Al-LLZO thin film after annealing at 660 °C with solid lines representing fitted curves. **i** Arrhenius plot of in-plane total ionic conductivity for selected thin-film samples. Reproduced with permission from Ref. [150] Copyright 2019 Springer Nature Publishing

Zhao et al. [147] synthesized anti-perovskite Li_3_OCl thin films via composite-target PLD, achieving room-temperature ionic conductivity of 2.0 × 10^−4^ S cm^−1^—two orders of magnitude higher than bulk analogues, and demonstrated the first TFMB with Li_3_OCl as the electrolyte (Fig. 5e, f). Xu et al. [148] fabricated amorphous Li_1.3_Al_0.3_Ti_1.7_(PO_4_)_3_ films via MS, achieving 6.47 × 10^−6^ S cm^−1^ conductivity and ultralow electronic conduction (2.34 × 10^−14^ S cm^−1^) (Fig. 5g, h). Zhang et al. [149] optimized amorphous Li_0.33_La_0.56_TiO_3_ thin films by tuning oxygen partial pressure and post-annealing, reaching conductivity of 5.32 × 10^−5^ S cm^−1^ and activation energy of 0.26 eV (Fig. 6a–d). These studies highlight the need to balance oxygen defect suppression and Li preservation for high-performance oxides.

#### Low-Temperature Processing and Interface Engineering

To mitigate the high processing temperatures of ceramic SSEs, Rupp et al. [150] developed a multilayer fabrication approach for lithiated Al-doped Li_7_La_3_Zr_2_O_12_ (Al-LLZO) thin films. Sequential Li_3_N/Al-LLZO bilayers deposited by PLD and mild annealing stabilized the cubic phase at temperatures 400 °C lower than conventional sintering, yielding conductivity of (2.9 ± 0.05) × 10^−5^ S cm^−1^ (Fig. 6e–i). This strategy reduces thermal budgets and enhances scalability for TFMB fabrication.

In summary, LiPON remains the benchmark SSE, offering unmatched electrochemical stability, interfacial reliability, and process versatility across deposition platforms (RFMS, PLD, e-beam, ALD). Its amorphous nature enables facile thin-film fabrication and excellent compatibility with LCO cathodes and Li anodes, ensuring dendrite-free cycling. Nevertheless, moderate ionic conductivity (10^−6^–10^−5^ S cm^−1^), deposition-rate limitations, and moisture sensitivity remain challenges. Emerging crystalline SSEs and engineered oxides offer promising alternatives with higher conductivities, but often demand high-temperature processing and face interfacial issues (Table 5). Moving forward, the balance between conductivity, interfacial stability, and scalable low-temperature processing will dictate the next-generation of SSEs for TFMB applications.

**Table 5:** Representative thin-film solid electrolytes for TFMBs

Material	Ionic conductivity (S·cm^−1^)	Electrochemical window (V)	Deposition Method	References
LiPON	6.4 × 10^−6^	5.5	MS	[140]
LLZO	2 × 10^−4^	6	MS, PLD	[150]
LLTO	8.2 × 10^−4^	2.8	MS, PLD	[149]
Li_3_OCl	2 × 10^−4^	6	PLD	[147]
LATP	2.46 × 10^−5^	2.4	MS, PLD	[148]

### Fabrication and Integration of TFMBs

The rapid evolution of modern microelectronics has triggered increasing demand for power sources that are not only miniaturized, lightweight, and ultra-thin but also mechanically flexible, low-power, and seamlessly integrable into highly compact systems. In this regard, TFMBs, as a crucial subclass of all-solid-state lithium batteries (ASSLBs), have garnered extensive attention owing to their intrinsic safety, exceptional volumetric energy density, and facile compatibility with on-chip integration. Nevertheless, despite substantial progress in individual material components, the realization of fully integrated TFMBs remains nascent. Key challenges persist, particularly those associated with interfacial stability, scalable thin-film processing, and microstructural control. Successful construction of high-performance TFMBs necessitates not only a profound understanding of the intricate physicochemical interactions among electrodes, SSEs, and their interfaces, but also the development of advanced fabrication techniques capable of delivering high-quality films with precise composition control and interfacial conformity. Within this context, PVD techniques, especially MS and PLD, have emerged as promising methods due to their industrial maturity, high deposition rates, excellent uniformity, and scalability, rendering them highly compatible with wafer-scale microfabrication processes.

**Fig. 7:**
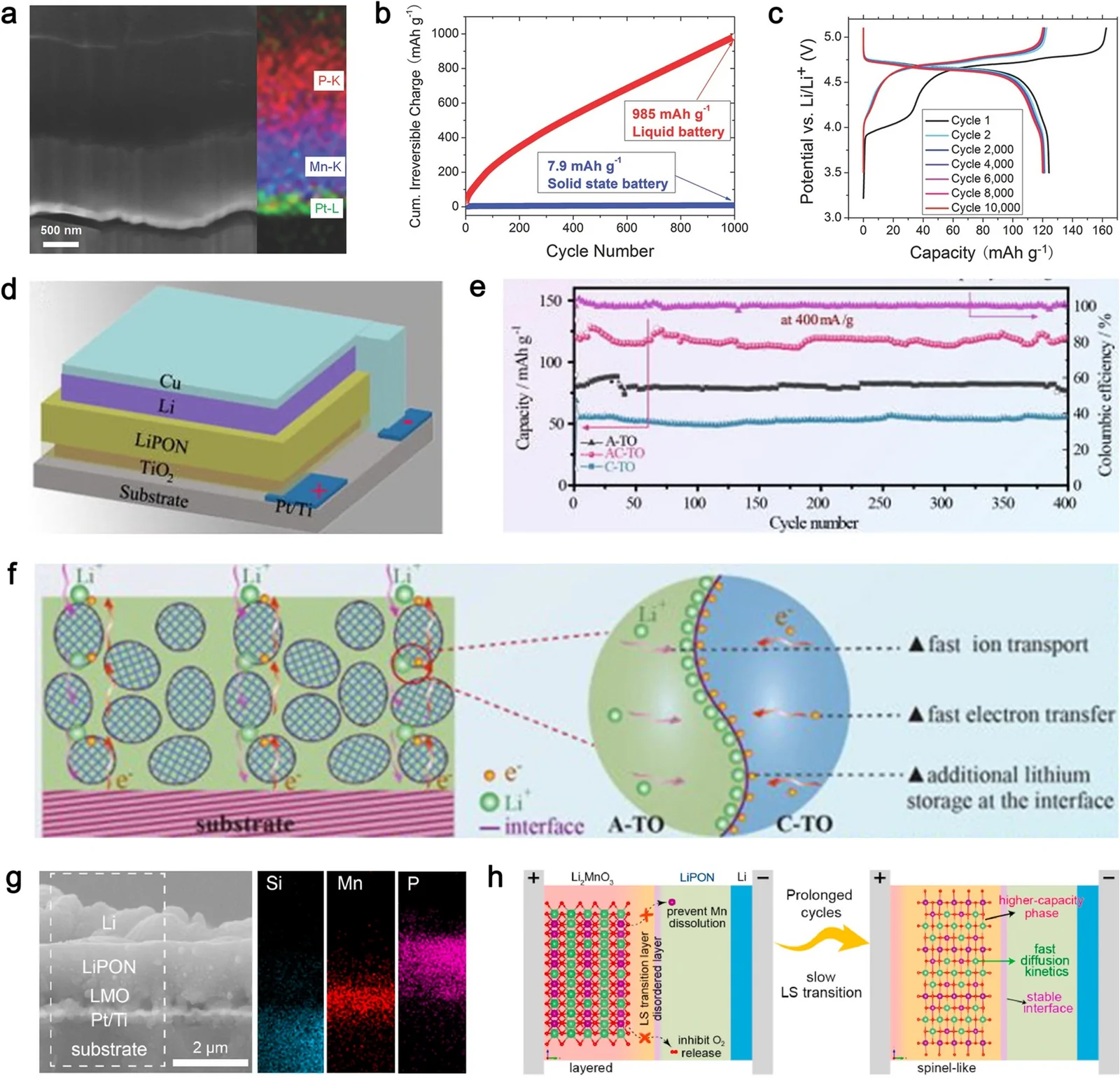
Electrochemical properties, degradation pathways, and structural mechanisms of cathode-based TFMBs. **a** Cross-sectional SEM image and corresponding EDS elemental mapping of the LNMO-based TFMB after 1000 cycles, showing structural integrity and elemental distribution. **b** Comparison of cumulative irreversible charges for LNMO electrodes in TFMBs versus conventional LIBs. **c** Voltage profiles of LNMO-based TFMBs at different cycle numbers under 0.1 C. Reproduced with permission from Ref. [151] Copyright 2015 Wiley Publishing. **d** Schematic illustration of the device architecture of a TiO_2_-based TFMB. **e** Cycling performance comparison of TFMBs with A-TO, AC-TO, and C-TO thin-film cathodes. **f** Schematic mechanism of performance enhancement in AC-TO thin-film electrode. Reproduced with permission from Ref. [152] Copyright 2022 AIP Publishing. **g** Cross-sectional FESEM image and corresponding EDS mapping of an LMRO-based TFMB. **h** Schematic illustration of the mechanism contributing to extended cycling life in LMRO-based TFMBs. Reproduced with permission from Ref. [153] Copyright 2024 Elsevier Publishing

In an early seminal study, Dudney et al. [151] fabricated a high-voltage TFMB using a spinel LiNi_0.5_Mn_1.5_O_4_ (LNMO) cathode, LiPON electrolyte, and Li metal anode. Cross-sectional SEM/EDS analyses revealed elemental migration and interfacial heterogeneity. The device operated stably at 4.7 V, retaining 90% of its initial capacity after 10,000 cycles (< 0.001% fading per cycle), with slight capacity loss at higher currents attributed to gradual resistance buildup. The first-cycle irreversible capacity (~ 40 mAh g^−1^ at the 4 V plateau) originated from the Mn^3+^/Mn^4+^ redox process rather than electrolyte decomposition, while subsequent voltage–capacity profiles remained unchanged over extended cycling. The wide electrochemical window of LiPON effectively suppressed parasitic reactions, enabling near-ideal coulombic efficiency (Fig. 7a–c). This work validated spinel-type cathodes in TFMBs and underscored the necessity of optimizing phase purity and minimizing compositional heterogeneity. To address the low capacity and rate limitations of TiO_2_ anodes, Xia et al. [152] designed an amorphous-crystalline heterostructure TiO_2_ via room-temperature MS. While amorphous TiO_2_ offers rapid Li^+^ transport but poor electronic conductivity, crystalline TiO_2_ ensures superior electronic transport yet limited Li^+^ mobility. The heterostructure integrates both advantages: amorphous regions provide rapid diffusion channels, rutile crystalline phases enhance electronic conductivity, and heterointerfaces offer additional Li^+^ storage and internal electric fields, facilitating coupled ion–electron transport and stabilizing interfaces. This synergistic design achieved a reversible capacity of 204 mAh g^−1^ at 50 mA g^−1^ and 73 mAh g^−1^ at 1600 mA g^−1^ respectively, with nearly 100% retention over 400 cycles and stable impedance (Fig. 7d–f), demonstrating an effective strategy for anode engineering in TFMBs.

**Fig. 8:**
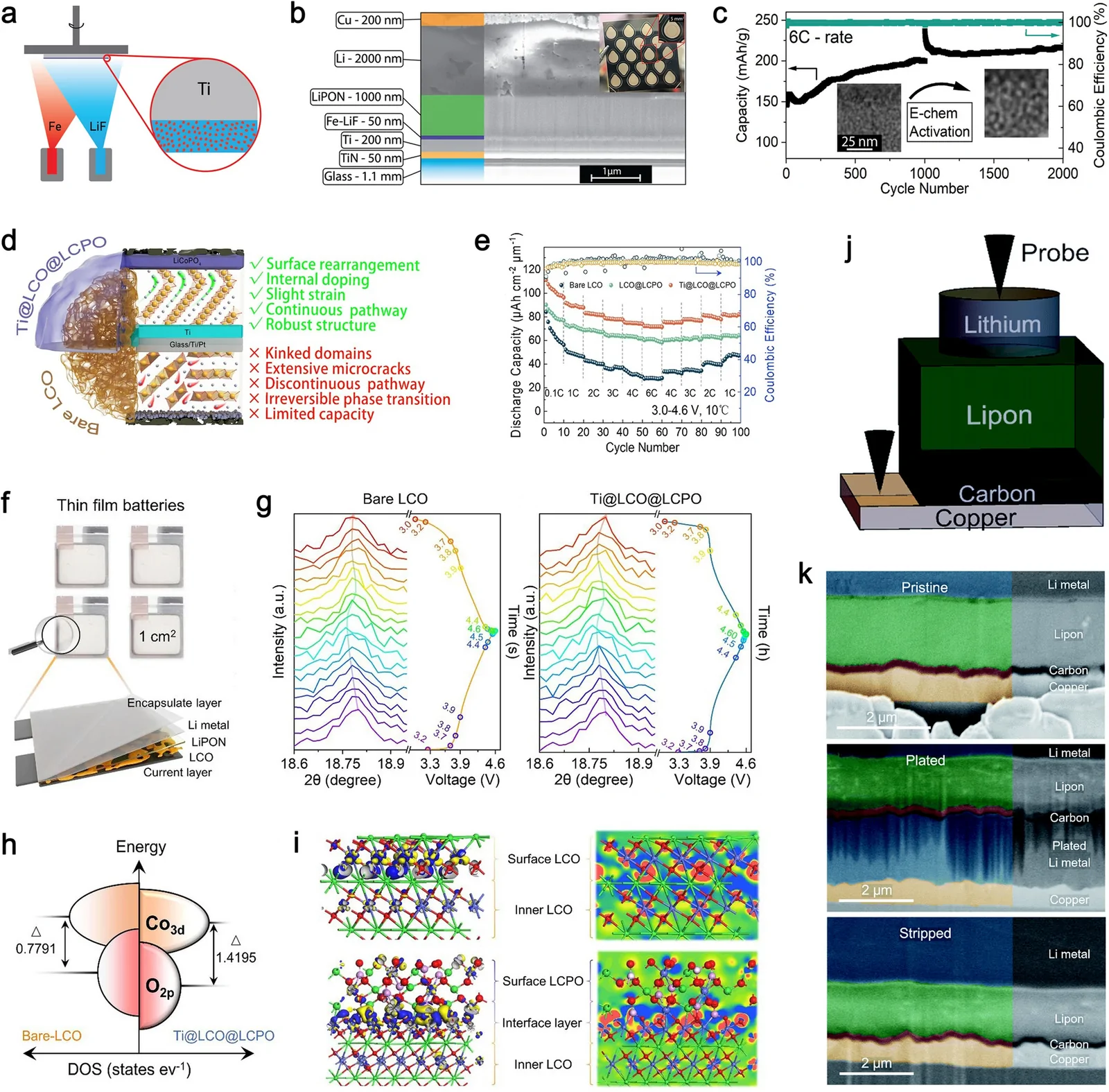
Structural design, interfacial engineering, and electrochemical performance of advanced cathodes and interlayers in TFMBs. **a** Schematic of the co-evaporation process for fabricating Fe–LiF composite cathodes. **b** Cross-sectional FIB-SEM image of a TFMB; inset shows the optical image of an as-deposited cell array and an enlarged view of a single microbattery. **c** Cycling performance of Fe–LiF cathodes at high current density. Reproduced with permission from Ref. [154] Copyright 2024 ACS Publishing. **d** Schematic of the dual-modification strategy for LCO thin-film cathodes, showing structural stabilization and ion diffusion pathways at the interface. **e** Rate performance comparison of modified LCO-based TFMBs tested at 10 °C. **f** Photograph and structural schematic of a representative TFMB device. **g** In situ XRD patterns of bare LCO and Ti@LCO@LCPO electrodes. **h** Energy-level diagram depicting the band gap between Co_3d_ and O_2p_ orbitals. **i** 2D charge density distribution slices illustrating the electronic structure evolution at the cathode interface. Reproduced with permission from Ref. [155] Copyright 2024 Elsevier Publishing. **j** Schematic of a full-cell configuration with Cu/C/LiPON/Li architecture. **k** Cross-sectional SEM images showing the evolution of the carbon interlayer at the current collector/solid electrolyte interface, including the as-prepared state, after Li plating, and after stripping at 0.2 mA cm^−2^. Reproduced with permission from Ref. [158] Copyright 2022 RSC Publishing

Further advancing cathode design, Xia et al. [153] integrated Li-rich Mn-based layered oxides (LMROs, e.g., Li_2_MnO_3_), into TFMBs. Compared with LMRO-based LIBs, the LMRO/LiPON/Li configuration exhibited enhanced stability by suppressing electrode degradation (Fig. 7g). LiPON stabilized interfaces, reduced impedance, and facilitated smooth Li^+^ transport while preventing Mn dissolution and O_2_ release. Extended cycling induced a controlled layered-to-spinel transition, improving crystallinity, conductivity, and Li^+^ diffusion, thereby accelerating ion–electron kinetics (Fig. 7h). The LMRO-TFMB maintained capacity over 1000 cycles, in stark contrast to conventional LMRO-LIBs, which suffered severe degradation. Transition-metal fluorides (TMFs) have also been studied as high-capacity cathodes. Casella et al. [154] fabricated Fe–LiF thin-film cathodes by co-evaporation and integrated them into LiPON-based TFMB (Fig. 8a, b). Electrochemical activation induced nanoscale reorganization, progressively increasing voltaic efficiency and reversible capacity up to 480 mAh‧g^−1^ at room temperature (Fig. 8c). These findings confirm the feasibility of conversion-type reactions in thin-film configurations.

For high-voltage LCO cathodes (> 4.55 V), Cui et al. [155] implemented dual-surface/interior modification by constructing a Ti@LCO@LCPO trilayer (Fig. 8d). This design suppressed irreversible phase transitions and cobalt dissolution, retaining 75% of capacity after 500 cycles (Fig. 8e). In situ XRD and DFT analyses confirmed reversible lattice changes and suppressed oxygen redox, attributed to reinforced interfacial bonding and reduced vacancy formation (Fig. 8f–i) [156]. In parallel, facet engineering was demonstrated by Nb_2_O_5_@LCO nanosheets on rotated (003) substrates, reducing Li^+^ diffusion barriers and internal stress. The optimized TFMB displayed 72.5% retention after 500 cycles at 1.4 C and an energy density of 1.148 mWh cm^−2^ [157]. Efforts to optimize anode-free TFMBs have focused on stabilizing Li nucleation. Futscher et al. [158] introduced an amorphous carbon (a-C) interfacial layer deposited by DCMS between current collectors and SSEs (Fig. 8j). Acting as a Li nucleation template, the a-C layer minimized overpotential, enhanced plating uniformity, and increased critical current density fourfold while reducing lithium loss (Fig. 8k), thereby advancing anode-free TFMB development.

**Fig. 9:**
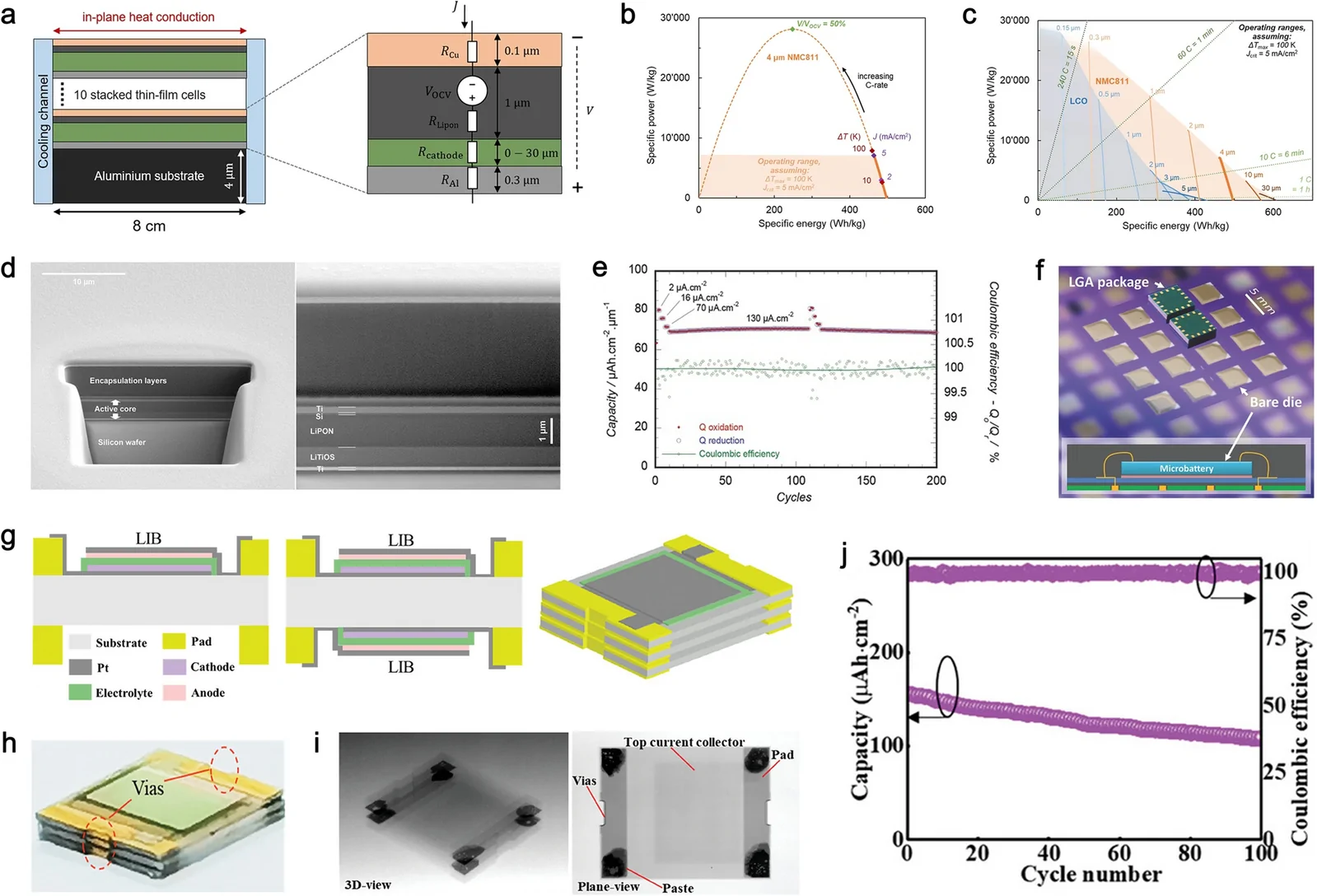
Modeling, architecture, and integration of stacked TFMBs for improved energy and thermal management. **a** Lumped thermo-electric model illustrating in-plane heat dissipation of series-stacked TFMBs through lateral cooling channels. **b** Ragone plot of a simulated stacked TFMB with a 4 μm-thick NMC811 cathode per cell, showing limitations from voltage efficiency, critical current density, and thermal gradients. **c** Energy-power characteristics of stacked TFMBs using LCO and NMC811 cathodes of varying thicknesses, with dashed lines indicating charge/discharge rates. Reproduced with permission from Ref. [164] Copyright 2023 Springer Nature Publishing. **d** FIB-SEM micrograph of an encapsulated TFMB consisting of Li_1.2_TiO_0.5_S_2.1_/LiPON/Si layers and Ti current collectors. **e** Electrochemical performance of the Li_1.2_TiO_0.5_S_2.1_-based TFMB, including capacity versus current density and cycling stability. **f** Photograph of encapsulated TFMB dies fabricated on an 8-inch silicon wafer, demonstrating wafer-scale integration. Reproduced with permission from Ref. [165] Copyright 2015 Wiley Publishing. **g** Schematic of a three-cell 3D-stacked LIB fabrication process. **h** Optical image of the assembled 3D-stacked LIB. **i** X-ray image confirming the stacked cell configuration. **j** Cycling performance of the 3D-stacked LIB at 125 °C, highlighting stability under elevated temperature conditions. Reproduced with permission from Ref. [166] Copyright 2024 Wiley Publishing

Beyond material innovations, novel device architectures are emerging [159–163]. Futscher et al. [164] proposed a monolithically stacked TFMB configuration, wherein multiple units are vertically integrated on a common substrate with precise lithographic patterning and nanoscale interfacial control. Modeling predicted enhanced energy–power characteristics under practical limits such as CCD and thermal gradients (Fig. 9a). With NMC811 cathodes (4 μm), Ragone plot indicated energy densities > 500 Wh kg^−1^ and power > 10 kW kg^−1^, depending on cathode thickness (Fig. 9b, c). Similarly, Pecquenard et al. [165] demonstrated an LCO/LiPON/Si TFMB fabricated directly on silicon wafer, achieving 1700 μWh cm^−2^‧μm^−1^volumetric energy density and exhibiting a reversible “memory effect” with potential for adaptive microsystems (Fig. 9d–f). Huang et al. [166] proposed a double-sided TFMB, fabricating symmetric battery units on both sides of a substrate and electrically paralleling them via conductive vias (Fig. 9g, h). This architecture doubled areal capacity without increasing footprint and maintained robust electrochemical performance at elevated temperatures (Fig. 9j). When extended to multilayered 3D stacks, enhanced stress distribution and adhesion mitigated delamination, enabling long-term stability.

**Fig. 10:**
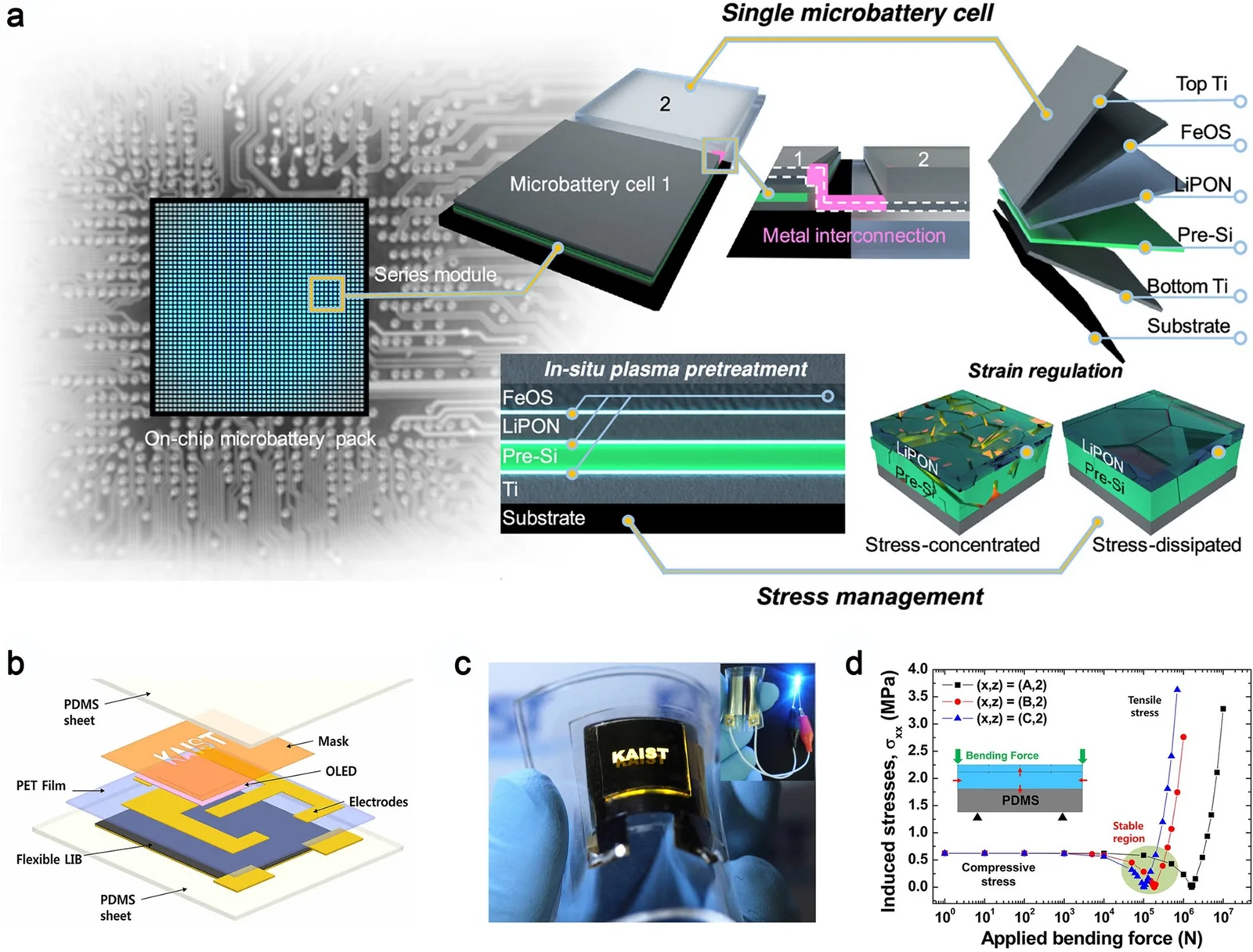
Design concepts and integration strategies of on-chip and flexible TFMBs tailored for advanced microelectronic systems. **a** Schematic illustration of on-chip TFMB configurations, including integrated packs, series-connected cells, exploded view of a single TFMB unit, and stress-relief strategies for reliability. Reproduced with permission from Ref. [167] Copyright 2025 PNAS Publishing. **b** Diagram of a fully flexible LED system powered directly by an integrated TFMB. **c** Photograph of an all-in-one flexible LED device incorporating a bendable thin-film battery, with inset showing stable LED illumination under bending. **d** Quantitative relationship between induced mechanical stress and applied bending force in the flexible TFMB system. Reproduced with permission from Ref. [168] Copyright 2012 ACS Publishing

Scalable, Si-compatible TFMB architectures have also been reported. Wang et al. [167] employed MS to fabricate iron oxysulfide cathodes and Si anodes, adopting an in situ prelithiation strategy to balance lithium distribution and improve mechanical durability. This system achieved high-rate operation (34.4 mA cm^−2^), ultralong cycle life (> 1,000,000 cycles), and integration into microfabricated arrays (Fig. 10a), highlighting manufacturability.

Flexibility represents another frontier. Lee et al. [168] demonstrated a TFMB-LED system on a mica substrate laminated onto PDMS, delivering 106 μAh cm^−2^ and > 94.5% retention after 100 cycles under repeated bending (Fig. 10b–d). Inverted flexible TFMBs fabricated by Hussain et al. [169] via substrate removal achieved 146 μAh cm^−2^ at 130 μA cm^−2^ but required precise processing, raising complexity. Together, these studies highlight that rational design, interfacial engineering, and microfabrication-compatible strategies can enable TFMBs with high capacity, thermal stability, and robust integration, paving the way for next-generation on-chip and wearable energy platforms.

### Interface and Interphase Evolution

The interfacial structure and its dynamic evolution at the solid–solid electrode/electrolyte junctions play a decisive role in determining the electrochemical performance, cycling stability, and rate capability of TFMBs [170–173]. Unlike conventional liquid-electrolyte LIBs, TFMBs adopt a planar, layer-by-layer solid-state configuration in which electrodes and electrolytes are coupled via defined two-dimensional contact interfaces. Due to the limited interfacial area and inherent lattice/chemical mismatches, such interfaces are highly susceptible to degradation during repeated lithiation/delithiation, leading to delamination, stress accumulation, and increased interfacial resistance. These challenges underscore the necessity of elucidating interfacial evolution mechanisms and developing effective stabilization strategies. However, direct probing of buried solid–solid interface remains inherently difficult, necessitating advanced, non-destructive techniques capable of resolving structural and chemical transformations with high spatial resolution.

**Fig. 11:**
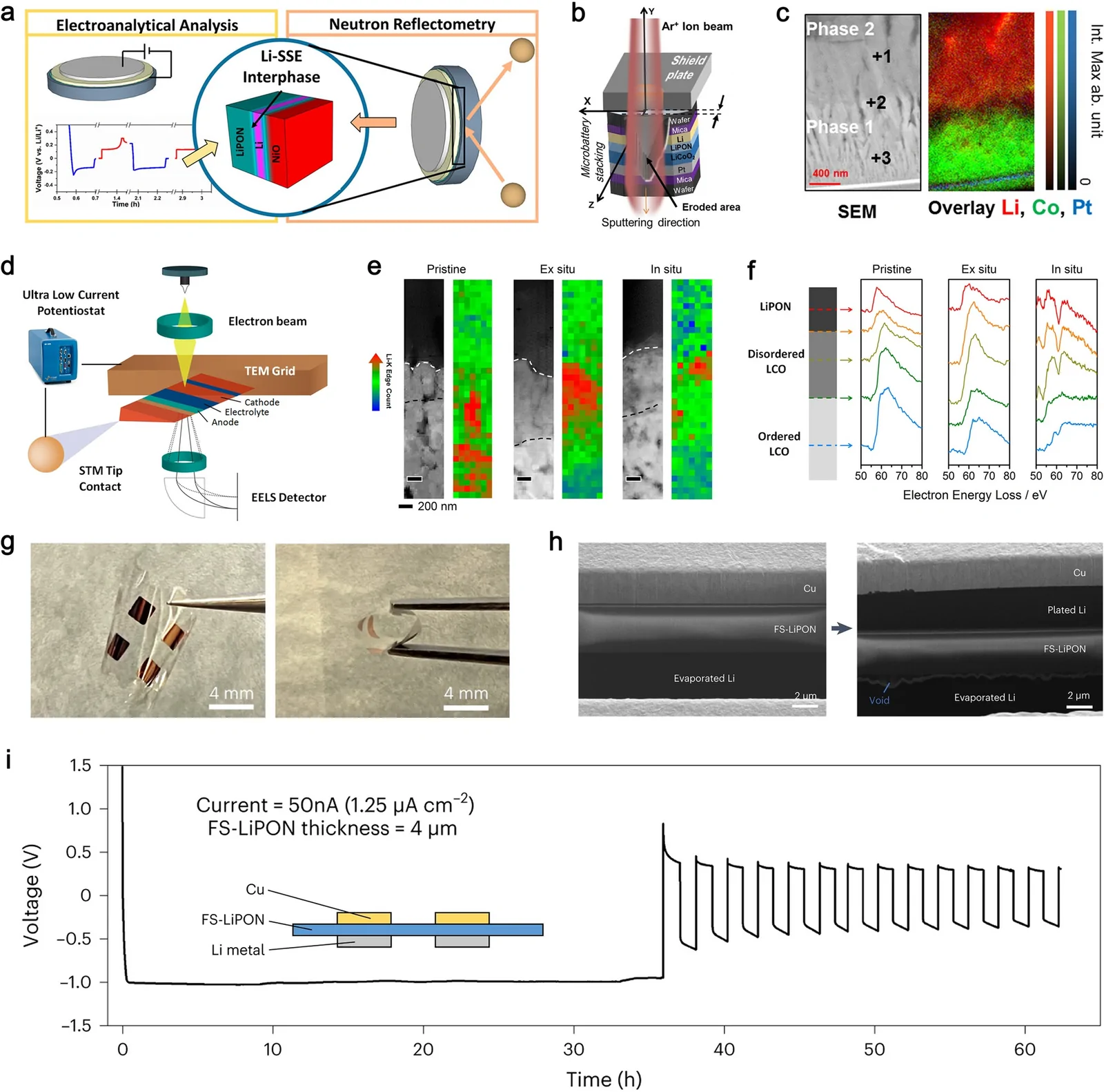
Advanced characterization and in situ analysis approaches for probing interfacial structure and electrochemical behavior in TFMBs. **a** Schematic illustration of the combined experimental methodology designed for multi-scale interfacial investigation. Reproduced with permission from Ref. [174] Copyright 2023 ACS Publishing. **b** Diagram of the cross-sectional polishing process employed for TFMB sample preparation. **c** Cross-sectional SEM and Auger electron microscopy images of an LCO/LiPON/Li TFMB, revealing chemical distribution at the interface. Reproduced with permission from Ref. [173] Copyright 2017 ACS Publishing. **d** Schematic of an in situ TEM nanobattery setup with triangular configuration, where the cathode is fixed to the grid and the anode is contacted by a piezo-controlled STM tip. **e** High-angle annular dark-field STEM images showing structural details of the nanobattery stack. **f** Li K-edge elemental mapping across different samples, revealing Li distribution under electrochemical operation. Reproduced with permission from Ref. [171] Copyright 2016 ACS Publishing. **g** Photographs of a freestanding LiPON-based Li–Cu cell in both flat and bent states. **h** Cryo-FIB/SEM cross-sectional images of the Li–Cu cell before and after Li plating, demonstrating morphological changes. **i** Voltage profile of Li plating/stripping in the freestanding LiPON Li–Cu cell. Reproduced with permission from Ref. [180] Copyright 2023 Springer Nature Publishing

Katie et al. [174] constructed a model Li/LiPON/NiO TFMB (Fig. 11a), employing neutron reflectometry (NR) to investigate the buried LiPON/NiO interface. Fitting experimental data with a five-layer structural model (treating LiPON as semi-infinite) enabled extraction of scattering length density (SLD) profiles. Upon Li deposition, the SLD of the NiO layer decreased to ~ 5.2 × 10^−6^ Å^−2^, indicating lithiation and formation of Li_x_NiO_y_. Concurrently, a thin Li layer (~ 2.4 nm) with SLD close to metallic Li (–0.88 × 10^−6^ Å^−2^) emerged at the LiPON/NiO interface. Even after Li stripping, a residual Li-rich interphase (SLD ≈ –0.1 × 10^−6^ Å^−2^) persisted, confirming the dynamic restructuring of the solid–solid boundary.

To spatially resolve interfacial chemistry, Uhart et al. [173] combined ion milling with auger electron spectroscopy (AES) to construct high-resolution elemental depth profiles across TFMB cross-sections (Fig. 11b). At the LCO/LiPON interface, a bilayer interfacial structure was identified (Fig. 11c): an upper Li-rich, Co-deficient region and a lower nearly delithiated Co_2_O_3_-like layer. While effective, ion-beam and TEM-based routes risk beam-induced damage, motivating the pursuit of non-destructive alternatives. Using operando neutron depth profiling (NDP), Notten et al. [175] monitored extended cycling of LCO/Li_3_PO_4_/Si TFMBs, revealing progressive formation of a Si-containing interlayer at the Si/SSE boundary due to Si diffusion, which impeded Li^+^ transport and induced continuous capacity fading over 250 cycles. Complementarily, Thompson et al. [176] employed potentiostatic analysis to show that in amorphous Si, Li^+^ diffusion within the Li_x_Si phase—not interfacial charge transfer—constitutes the rate-limiting step, providing critical mechanistic insight.

LiPON, as the most widely developed amorphous SSE in TFMBs, is particularly vulnerable to environmental degradation, especially under air exposure [177]. Meng et al. [171] utilized in situ scanning transmission electron microscopy (STEM) combined with electron energy loss spectroscopy (EELS) to probe the LCO/LiPON interface (Fig. 11d). Even without cycling, a disordered LCO-derived layer formed spontaneously. Under in situ charging, high-valence Co species and insulating Li_2_O/Li_2_O_2_ compounds emerged (Fig. 11e, f), impeding interfacial Li^+^ transport. Subsequent studies under elevated temperatures (80 °C) further confirmed accelerated interphase growth, sharply increasing interfacial resistance and capacity fading [178]. Wang et al. [179] using in situ TEM directly observed the formation of electrochemically unstable nanocrystalline interphases that developed voids upon cycling, creating bottlenecks for ion conduction.

To enable external manipulation of interfaces, Meng et al. [180] developed freestanding LiPON (FS-LiPON) membranes via a combination of spin coating, RFMS, and solvent-based film transfer (Fig. 11g–i). Comprehensive characterization (SSNMR, DSC, nanoindentation) revealed that uniform and dense Li deposition could be achieved at Cu/FS-LiPON interfaces even without applied pressure, particularly when a gold seed layer was introduced to mitigate interfacial stress. This strategy offers a perspective avenue for pressure-free solid–solid contact engineering in TFMBs.

In short, interfacial and interphase phenomena in TFMBs are highly dynamic, governed by material selection, electrochemical cycling, and environmental conditions. Emerging in situ/operando characterization techniques, including NR, NDP, AES, STEM-EELS, and TEM, are providing unprecedented insights into interfacial evolution. These findings establish a mechanistic foundation for rational interface design and highlight pathways toward overcoming interfacial bottlenecks that currently limit the long-term reliability and scalability of TFMBs.

### Interfacial Modification Strategies for TFMBs

**Fig. 12:**
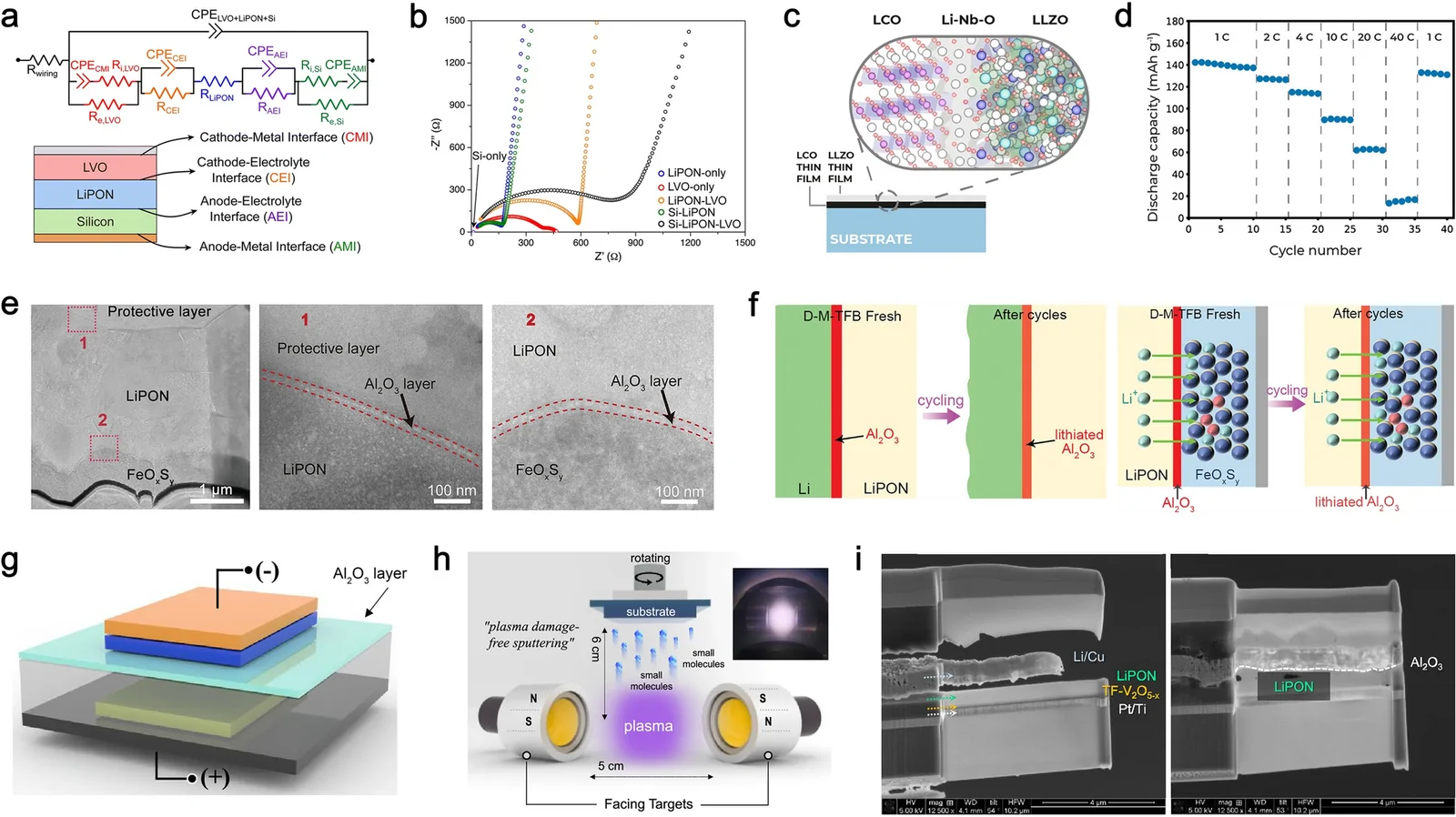
Electrochemical modeling, interfacial optimization, and structural protection strategies for enhancing the performance and stability of TFMBs. **a** Equivalent circuit model applied to interpret the impedance behavior of TFMBs. **b** Impedance spectra of individual auxiliary components and the full as-deposited solid-state stack measured at OCV. Reproduced with permission from Ref. [181] Copyright 2025 RSC Publishing. **c** Schematic illustration of the solid–solid interface between LLZO and LCO thin films. **d** Discharge capacities of LCO/Li–Nb–O/LLZO half-cells tested under different C-rates. Reproduced with permission from Ref. [182] Copyright 2020 ACS Publishing. **e** TEM image of a dual-modified TFMB with enlarged views of interfacial regions at LiPON/Al_2_O_3_ and FeO_x_S_y_/Al_2_O_3_/LiPON. **f** Schematic representation of stabilized LiPON/Al_2_O_3_/Li and FeO_x_S_y_/Al_2_O_3_/LiPON interfaces during repeated cycling. Reproduced with permission from Ref. [184] Copyright 2024 Wiley Publishing. **g** Schematic of a TFMB design incorporating a protective ultrathin Al_2_O_3_-coated LiPON layer. **h** Diagram of the FTS technique used for thin-film deposition. **i** FIB-SEM images comparing pristine LiPON electrolytes with those protected by ultrathin Al_2_O_3_ coatings, showing suppressed interfacial degradation. Reproduced with permission from Ref. [185] Copyright 2021 ACS Publishing

Interfacial instability, manifested in both chemical and structural forms, remains a major limitation to achieving long-term stability and high performance in TFMBs. Owing to the intrinsic solid–solid contact between electrode and electrolyte layers, interfacial regions frequently suffer from mechanical delamination, chemical interdiffusion, and sluggish Li^+^ transport kinetics. Consequently, targeted interfacial engineering is essential to minimize resistance, suppress parasitic reactions, and enable robust electrochemical cycling. To enable precise diagnosis of interfacial behavior in TFMBs, Stewart et al. [181] proposed an integrated diagnostic methodology based on microfabricated test structures. By simultaneously fabricating TFMB stacks and equivalent diagnostic components, electrochemical impedance spectroscopy (EIS) was performed to construct equivalent circuit models of individual layers and interfaces (Fig. 12a, b). The analysis revealed that the anode/SSE interface, particularly under dynamic cycling, exhibits pronounced kinetic instability, which was identified as the dominant degradation pathway. This diagnostic framework gives valuable insight for interface-specific optimization in advanced TFMBs. Sastre et al. [182] constructed a multilayer TFMB architecture (Si/MgO/Ti/Pt/LCO/Li–Nb–O/LLZO) to research cathode/SSE interfacial behavior. A lithiated Nb_2_O_5_ diffusion layer was introduced in situ at the LLZO/LCO interface, which significantly reduced interfacial impedance and facilitated fast charge transport (Fig. 12c, d). The Nb-rich interphase acted as a Li^+^-conductive buffer, mitigating lattice mismatch and electronic insulation, thereby enhancing long-term cycling stability. To further evaluate cathode/SSE compatibility, Kim et al. [114] investigated the interface between sputtered LCO and a NASICON-type SSE (Li_1.3_Al_0.3_Ti_1.7_(PO_4_)_3_, LATP). A 500 nm LCO film was deposited via RF sputtering and post-annealed at 500 °C to induce crystallization. The assembled TFMB (Pt/LCO/LICGC/LiPON/Li) was tested at 0.01 C and 30 °C within 3.3–4.2 V. Although elemental interdiffusion was negligible over 10 cycles, the initial coulombic efficiency was only 68%, primarily due to structural defects within the LCO film [183]. Nonetheless, this study demonstrates the feasibility of creating chemically stable cathode/SSE interfaces via thermal activation. Xia et al. [184] examined interfacial degradation in an anode-free TFMB composed of an amorphous FeO_x_S_y_ cathode, LiPON SSE, and Li metal anode. Severe structural disruption occurred at both FeO_x_S_y_/LiPON and LiPON/Li interfaces, leading to rapid capacity fading. To address this, a dual-interface engineering strategy was proposed by introducing amorphous Al_2_O_3_ interlayers at both cathode/SSE and SSE/anode interfaces (Fig. 12e, f). The Al_2_O_3_ buffer effectively suppressed Fe diffusion, mitigated Li-vacancy formation, and stabilized impedance evolution during cycling, thereby enhancing cycling stability and rate capability. This dual-interface route presents a generalized strategy for stabilizing both electrochemical and mechanical aspects of solid–solid interfaces.

The formation of unstable nanocrystalline interfacial layers during cycling has been widely recognized as a key origin of high resistance and Li^+^ transport bottlenecks. Wang et al. [179] employed in situ TEM to capture real-time Li⁺ transport dynamics at all-solid-state interfaces. Their observations revealed void formation and interfacial reconstruction driven by electrochemical/mechanical instabilities, verifying the critical role of interfacial microstructure in governing Li^+^ migration. To address the poor wettability and limited adhesion between metallic Li and LiPON, Kim et al. [185] introduced an ultrathin Al_2_O_3_ surface-modification layer via face-to-face target sputtering (FTS) (Fig. 12g, h). Time-of-flight secondary ion mass spectrometry (TOF–SIMS) characterization revealed that without modification, Li exhibited poor adhesion and tended to delaminate from the current collector. With the Al_2_O_3_ interlayer, interfacial contact was significantly improved, delamination was suppressed, and Li nucleation became more uniform. Post-cycling analysis confirmed a substantial reduction in interfacial resistance, validating the efficacy of this engineering strategy (Fig. 12i).

Overall, interfacial resistance in TFMB arises mainly from chemical/electrochemical incompatibility, poor contact, and strain induced by lithiation/delithiation. Interfacial engineering, through dielectric nanoparticle incorporation, ion-conductive interlayers, and thermal treatment, has proven effective in mitigating these issues. For instance, BaTiO_3_ nanoparticles at the LiCr_0.05_Ni_0.45_Mn_1.5_O_4–δ_/LiPON interface reduce local potential differences, thereby promoting Li^+^ migration and improving both capacity and rate performance [186]. Similarly, LiNbO_3_ interlayers at the LCO/LiPON interface regulate Li^+^ distribution: low-temperature deposition extracts surface Li^+^ to boost low-rate capacity, while high-temperature deposition forms a Li^+^-rich interphase that lowers resistance [187]. Thermal treatment has also been shown to restore crystallinity and optimize Li^+^ pathways, as demonstrated in LMO and LCO interfaces [188, 189]. Collectively, these strategies, encompassing nanoparticle modification, ion-conductive buffers, thermal annealing, and structural reconstruction, substantially enhance interfacial compatibility, lower resistance, and improve electrochemical performance. Continued progress in interfacial material design and high-resolution characterization will be critical for enabling stable, integrable, and high-performance TFMBs.

## Emerging Strategies and Novel Approaches

### 3D TFMBs and Spatial ALD

Conventional 2D TFMBs inherently suffer from limited areal energy and power densities due to constrained electrode–electrolyte interfacial areas and degradation of Li^+^ transport kinetics with increasing film thickness [190, 191]. To overcome these bottlenecks, 3D TFMBs have emerged as promising solution. By exploiting high-aspect-ratio microstructures, 3D TFMBs significantly expand the effective electrochemical surface area without enlarging the device footprint, thereby enhancing both energy and power densities [191, 192]. Depending on the fabrication route, 3D TFMBs can be broadly classified into two categories: (1) those constructed on pre-patterned 3D substrates and (2) self-standing electrode-based configurations.

**Fig. 13:**
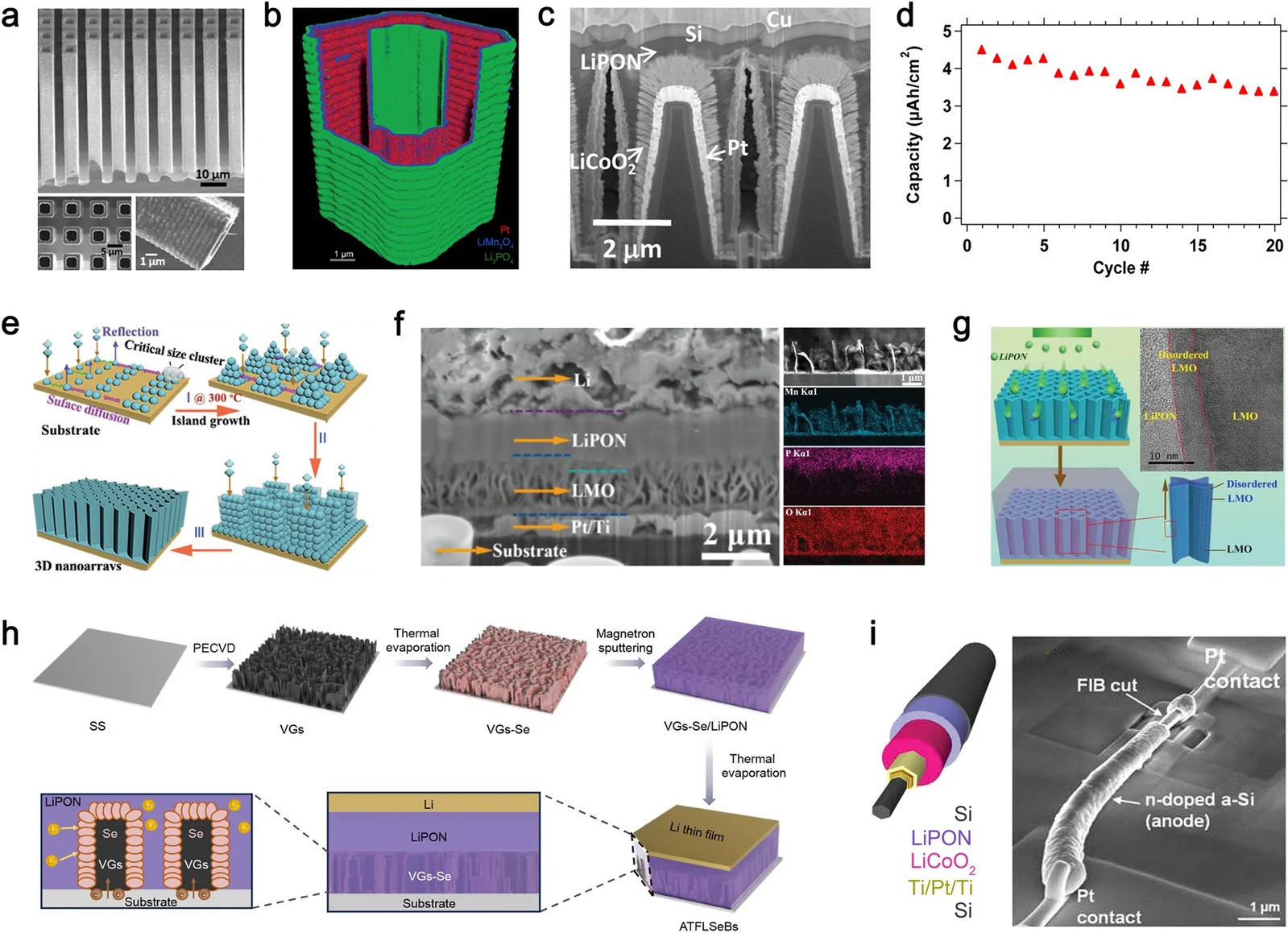
3D architectures and nanoscale integration strategies for improving the electrochemical performance of thin-film and nanowire-based microbatteries. **a** SEM images of 3D structured electrodes designed to increase effective surface area and electrochemically active interfaces. **b** X-ray transmission microscopy images visualizing 3D electrode frameworks deposited via ALD. Reproduced with permission from Ref. [195] Copyright 2022 Wiley Publishing. **c** FIB cross-sectional images of a 3D solid-state LIB (SSLIB), revealing the layered arrangement. **d** Cycling performance of the 3D SSLIB at 1.2 C. Reproduced with permission from Ref. [196] Copyright 2016 ACS Publishing. **e** Schematic illustration of 3D LMO thin-film deposition using controlled growth modes. **f** Cross-sectional FESEM and STEM images of a 3D TFMB, accompanied by corresponding EDS elemental mapping. **g** Schematic representation of disordered LMO formation at the LMO/LiPON interface in 3D TFMBs, with inset HRTEM image of the interfacial region. Reproduced with permission from Ref. [130] Copyright 2018 Wiley Publishing. **h** Schematic of the fabrication process for an all-solid-state TFMB based on a Li–Se electrochemical system. Reproduced with permission from Ref. [198] Copyright 2025 Wiley Publishing. **i** Schematic and FESEM images of a nanowire LIB integrated with Pt electrodes on a Si/SiO_2_ substrate. Reproduced with permission from Ref [197]. Copyright 2012 ACS Publishing

The advent of conformal thin-film deposition techniques, particularly ALD, has enabled the precise layer-by-layer construction of TFMBs on substrates with complex geometries. Gregorczyk et al. [193] pioneered a fully conformal TFMB comprising of Ru/LiV_2_O_5_/Li_2_PO_2_N/SnN_x_/TiN/Cu multilayers grown by ALD on 3D silicon scaffolds, demonstrating electrochemical performance consistent with finite-element simulations. Similarly, Lethien et al. [194] deposited Al_2_O_3_/Pt/TiO_2_/Li_3_PO_4_ multilayers on silicon microtube arrays, achieving areal capacities nearly two orders of magnitude higher than planar analogs despite ultrathin active layers (< 100 nm). Building upon this concept, Lethien’s group [195] realized a high-performance 3D LMO electrode via ALD on functionalized current collectors (Fig. 13a, b), achieving stable operation at 4.1 V vs. Li/Li^+^ and delivering capacities of 180 μAh cm^−2^ for a 100 nm film at 0.05 C, without requiring prelithiation. TEM and X-ray transmission microscopy confirmed excellent conformality and interfacial integrity, highlighting ALD’s potential for CMOS-compatible TFMB fabrication. Despite its precision, ALD is intrinsically limited by its ultralow growth rate (~ 1.2 Å/cycle) and high cost, constraining scalability for practical 3D TFMBs. Thus, alternative conformal deposition methods are being actively explored. Talin et al. [196] compared 2D and 3D TFMBs fabricated via PVD on conical and cylindrical micropillar arrays (Fig. 13c, d). The inferior performance of 3D devices was attributed to non-uniform film coverage arising from the line-of-sight nature of PVD, implying the importance of morphological uniformity. Extending this concept, Ruzmetov et al. [197] fabricated nanowire-based core–shell TFMBs using PVD (Fig. 13i), achieving nanoscale operability but with limited power density because of structural discontinuities. These findings emphasize that conformal deposition is a pivotal factor in enabling high-performance 3D TFMBs.

In another approach, Wang et al. [198] combined PECVD, thermal evaporation, and MS to construct an all-solid-state Li–Se TFMB (Fig. 13h). A vertical graphene nanosheet (VGs) host (~ 600 nm) was first deposited onto stainless steel via PECVD, followed by conformal Se cathode coating, LiPON SSE deposition by MS, and Li metal anode deposition. The interconnected VGs framework provided high conductivity and mechanical resilience, suppressing polyselenide shuttling and ensuring stable operation over 5000 cycles at 25 μA cm^−2^ with a capacity of 5.1 μAh cm^−2^. This highlights the promise of 3D carbon frameworks for stabilizing interfaces and mitigating volume fluctuations in selenium-based cathodes.

Beyond templated-assisted deposition, self-standing electrodes based on vertically aligned nanostructures (e.g., nanorods, nanowalls, nanotubes) have also been pursued due to their high surface area and facile ion/electron transport [43]. Research efforts have primarily focused on self-standing anodes such as LTO, Si, and TiO_2_ [199–201], while robust 3D cathode development remains challenging due to the high-temperature synthesis required. Xia et al. [130] addressed this limitation by using a controlled PVD approach to construct 3D LMO cathodes via Volmer–Weber island growth (Fig. 13e) during DCMS at 300 °C, followed by post-annealing at 600–700 °C to yield crystalline nanosheet arrays. The resulting semi-3D TFMB exhibited reduced interfacial resistance and enhanced capacity relative to planar analogs (Fig. 13f, g). Expanding this strategy, α-MoO_3–x_ and MnO_2–x_ nanostructures were fabricated directly via reactive DCMS as cathodes for 3D TFMBs with amorphous LiPON and Li anodes [202], achieving specific capacities of 266 mAh g^−1^ at 50 mA g^−1^ and 92.7% capacity retention over 1000 cycles. More recently, a low-temperature (180 °C) reactive sputtering protocol enabled the fabrication of 3D Li_x_MnO_2_ nanosheet arrays, followed by Li^+^ injection through LiPON and mild annealing [64]. The resulting tunnel-type intergrowth cathode delivered 185 mAh g^−1^ at 50 mA g^−1^ and retained 81.3% capacity after 1000 cycles, demonstrating compatibility with both rigid and flexible substrates.

In summary, PVD-based strategies for 3D cathode design offer notable advantages in process simplicity, microfabrication compatibility, and potential wafer-level integration. However, limitations in active material loading and film thickness continue to restrict areal capacity. Future research should focus on hierarchical 3D structures with optimized porosity, thickness gradients, and layer conformality to balance performance with scalability. While PVD remains attractive for integration, the practical implementation of 3D TFMBs will likely require hybrid approaches that leverage the precision of ALD with the throughput of alternative scalable methods.

#### ALD on Pre-patterned Substrates

The intrinsically slow growth rate necessitates prolonged deposition to achieve sufficient film thickness, severely limiting throughput. ALD also requires stringent reaction control, complex precursor management, and high technical expertise, all of which increase equipment and operational costs. Furthermore, costly and sometimes toxic precursors, coupled with high energy consumption, further undermine economic feasibility for large-scale production.

#### PVD for Self-supported Nanostructures

PVD provides versatile material deposition but is limited in processing certain high-performance cathodes, restricting material diversity. Its typical growth mode constrains 3D film thickness, thereby limiting areal capacity and energy density. From an economic perspective, PVD involves higher fabrication costs and lower throughput compared with conventional LIB manufacturing. Additionally, surface roughness in PVD-fabricated 3D structures complicates downstream assembly and may require post-treatment, further increasing process complexity and cost.

Overall, both ALD and PVD exhibit clear potential for 3D TFMB fabrication, yet face intrinsic challenges in scalability, cost, and structural control. Addressing these issues will require innovative process design and materials strategies, ultimately enabling the translation of 3D TFMBs into practical microelectronic and IoT applications.

### Co-Deposition and Synergistic Processes

**Fig. 14:**
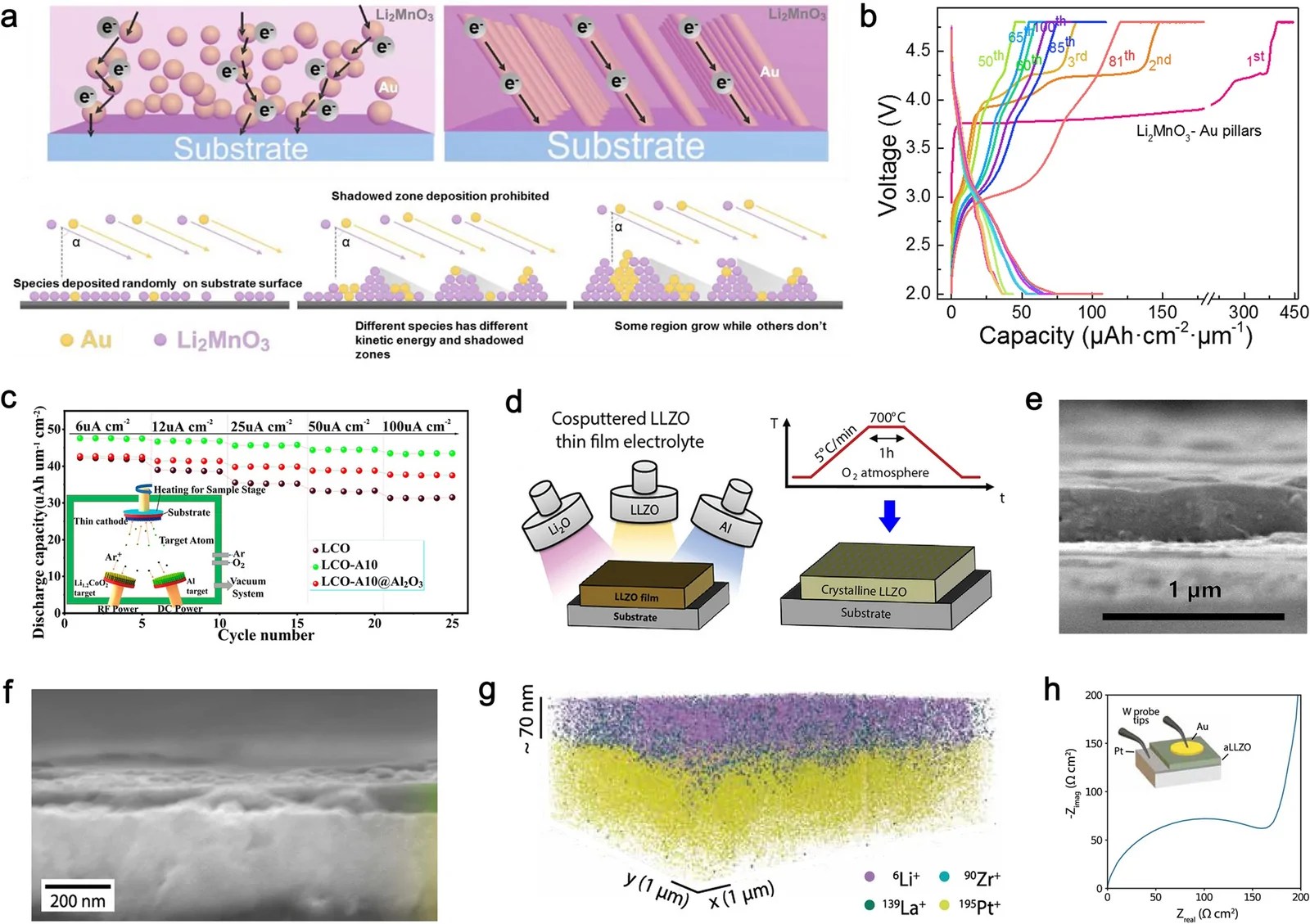
Compositional engineering and co-sputtering strategies for optimizing thin-film electrode and electrolyte performance in TFMBs. **a** Schematic comparison between Li_2_MnO_3_–Au particle and pillar composite structures, together with the proposed growth mechanism. **b** Cycling performance of Li_2_MnO_3_–Au composites over 100 cycles. Reproduced with permission from Ref. [204] Copyright 2020 Elsevier Publishing. **c** Rate performance of pristine LCO, LCO-A10, and LCO-A10@Al_2_O_3_ electrodes; inset shows schematic of the co-sputtering process used for composite film formation. Reproduced with permission from Ref. [205] Copyright 2022 ACS Publishing. **d** Schematic fabrication route of Al-doped LLZO thin films via co-sputtering followed by thermal annealing. **e** Cross-sectional SEM image of the Al:LLZO thin film, confirming controlled morphology. Reproduced with permission from Ref. [207] Copyright 2019 ACS Publishing. **f** Cross-sectional SEM image of amorphous LLZO (aLLZO) film deposited on a Pt-coated Si substrate. **g** Elemental tomographic mapping of aLLZO film acquired using GIS-assisted FIB–ToF–SIMS, highlighting spatial elemental distribution. **h** Nyquist plot of aLLZO films at room-temperature measured with blocking electrodes; inset shows schematic of the corresponding device configuration. Reproduced with permission from Ref. [209] Copyright 2021 Springer Nature Publishing

The rational design of electrode microstructures at the nanoscale has emerged as an effective strategy to regulate ion/electron transport kinetics and thereby enhance the overall electrochemical performance of TFMBs. In particular, co-deposition of functional components and the construction of synergistically integrated architectures offer new possibilities to overcome intrinsic limitations of conventional thin-film electrodes. Wang et al. [203] employed oblique angle pulsed laser deposition to fabricate titled columnar Li_2_MnO_3_ thin film, where the anisotropic morphology provided accelerated Li^+^ diffusion channels and enhanced redox activity. The resulting films exhibited significantly improved capacity and rate performance compared with planar counterparts. Building on this, the same group [204] developed Li_2_MnO_3_–Au hybrid columnar composite electrodes by simultaneous oblique deposition, embedding Au nanocolumns into the Li_2_MnO_3_ framework. Due to geometric shadowing, an angular deviation of ~ 19° between the Au nanostructures and the incident flux was observed, enabling high-quality epitaxial growth (Fig. 14a). This composite design created dual conduction pathways—continuous electronic transport through Au columns and ionic transport through the Li_2_MnO_3_ matrix—yielding markedly improved capacity retention and rate capability (Fig. 14b). These findings underscore the potential of co-deposition-induced heterostructures in achieving synergistic electrochemical enhancements.

Despite its high operating voltage and compatibility with thin-film platforms, LCO suffers from severe structural degradation during prolonged cycling. To address this, Dai et al. [205] developed an in situ Al-doped and Al_2_O_3_-coated LCO thin-film cathode via MS (Fig. 14c). Al incorporation simultaneously formed a LiAl_y_Co_1–y_O_2_ solid solution that enhanced Li^+^ diffusivity and structural integrity, while generating metallic Al-based conductive pathways. Meanwhile, the conformal Al_2_O_3_ coating effectively suppressed parasitic interfacial reactions and stabilized the cathode–electrolyte interface. As a result, the modified LCO electrode delivered a high areal capacity of 40.2 μAh cm^−2^ μm^−1^ over 240 cycles at 2.5 μA cm^−2^ with 94.14% retention, whereas the pristine electrode failed after 110 cycles. Furthermore, at a high current density of 100 μA cm^−2^, the modified film delivered 43.5 μAh cm^−2^ μm^−1^, representing a 38.97% improvement over the unmodified sample (31.3 μAh cm^−2^ μm^−1^). These results highlight the effectiveness of synergistic doping–coating strategies in simultaneously optimizing bulk and interfacial properties for durable TFMBs.

Garnet-type LLZO have attracted considerable attention due to its excellent thermal stability, broad electrochemical window, and high Li^+^ conductivity. However, achieving dense and stable LLZO thin films with high ionic conductivity remains challenging. Buecheler et al. [206] reported a co-sputtering approach using Li_2_O, LLZO, and Ga_2_O_3_ targets to synthesize Ga-doped LLZO (Ga-LLZO) thin films, followed by post-deposition annealing. Upon annealing at 700 °C, a tetragonal-to-cubic phase transformation yielded polycrystalline cubic Ga-LLZO films with in-plane ionic conductivity up to 1.6 × 10^−5^ S cm^−1^ at 30 °C. Grazing-incidence XRD (GIXRD) confirmed the temperature-dependent phase evolution. Follow-up work [207] demonstrated that excess Li and Al doping suppressed porosity, promoted densification, and mitigated proton-exchange degradation. Optimized Al-doped LLZO film (~ 400 nm thick) achieved 1.9 × 10^−5^ S cm^−1^ ionic conductivity with excellent air stability and compatibility for TFMB integration (Fig. 14d, e). Nevertheless, limited film density and Li volatility during high-temperature processing remained key issues. To solve this, Sastre et al. [208] synthesized submicron Ga- or Al-doped LLZO films via co-sputtering and annealing at 700 °C, achieving ionic conductivity as high as 1.9 × 10^−4^ S cm^−1^—one order of magnitude higher than conventional LiPON and comparable to bulk sintered LLZO. This breakthrough establishes a new performance benchmark for garnet-type thin-film SSEs, offering a scalable pathway for high-power, vacuum-compatible TFMBs.

The application of Li metal anodes in TFMBs is often hampered by dendritic growth, particularly in polycrystalline LLZO, where grain boundaries and defects act as preferential channels for dendrite initiation. Amorphous LLZO (aLLZO), with its dense, grain-boundary-free microstructure and negligible electronic conductivity, provides a viable solution. Romanyuk et al. [209] fabricated aLLZO films by MS and tuned Li stoichiometry to enhance ionic conductivity by up to four orders of magnitude while maintaining electronic insulation (Fig. 14f, g). Symmetric Li/aLLZO/Li cells demonstrated dendrite suppression under current densities up to 3.2 mA cm^−2^. Moreover, TFMBs with 70 nm-thick aLLZO electrolytes operated stably over 500 cycles at 10 C. When employed as an interfacial buffer atop crystalline LLZO, aLLZO significantly reduced interfacial impedance and increased critical current density (Fig. 14h). These results affirm the pivotal role of amorphous garnet-type films in enabling dendrite-free Li metal anodes, thereby advancing the safety and lifespan of TFMBs.

### Roll-to-Roll and Wearable Systems of Flexible TFMBs

With the accelerating advancement of portable and wearable electronics, the development of flexible and scalable energy storage systems has become a key enabler for next-generation device integration. Owing to their ultrathin and planar architectures, TFMBs are ideally suited for such applications. Recent progress has therefore focused on roll-to-roll fabrication strategies and mechanically adaptive designs to meet the requirements of high energy density, structural versatility, and large-area manufacturability.

#### Foldable Architectures and Micro-origami Strategies

**Fig. 15:**
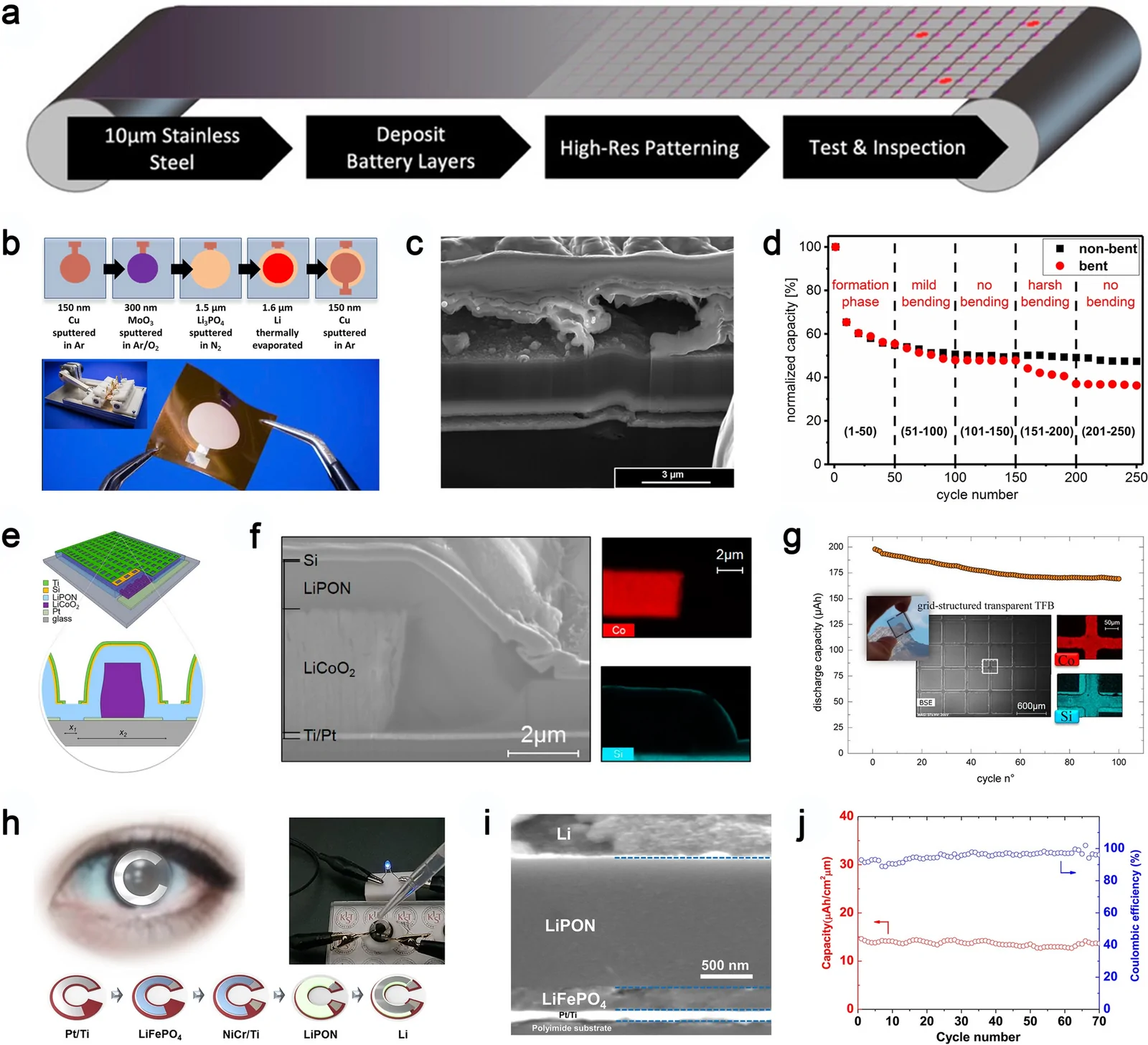
Scalable fabrication and multifunctional integration strategies of flexible, transparent, and wearable TFMBs. **a** Schematic of a high-throughput roll-to-roll deposition process enabling large-area TFMB fabrication. Reproduced with permission from Ref. [212] Copyright 2023 ACS Publishing. **b** Process scheme of a flexible MoO_3_-based TFMB constructed on a multilayer polyimide substrate; inset shows the custom-built bending apparatus. **c** Cross-sectional morphology of the flexible TFMB under severe bending conditions. **d** Comparison of discharge capacities between bent and unbent TFMBs, demonstrating mechanical reliability. Reproduced with permission from Ref. [215] Copyright 2019 Elsevier Publishing. **e** Schematic illustration of transparent TFMB architecture. **f** Cross-sectional SEM image and corresponding EDS elemental mapping of a transparent TFMB. **g** Cycling performance of the transparent TFMB; inset includes SEM and EDS mapping of the top grid together with a device photograph. Reproduced with permission from Ref. [228] Copyright 2019 ACS Publishing. **h** Illustration of TFMB integration within a smart contact lens platform. **i** Cross-sectional SEM image of the TFMB embedded in a smart lens. **j** Cycling performance of the lens-integrated TFMB over 70 cycles, confirming stable electrochemical output. Reproduced with permission from Ref. [229] Copyright 2018 Elsevier Publishing

To adapt TFMBs to foldable and curvilinear systems, several design prerequisites must be met: (1) electrodes and electrolytes must exhibit sufficient mechanical elasticity or be integrated with compliant substrates; (2) interfacial stress must be effectively mitigated to avoid delamination and microcracking; and (3) precise alignment during multilayer integration may be facilitated by external fields, such as magnetics [210]. In this context, Schmidt et al. [211] introduced a “micro-origami” strategy that employed mechanical fold lines to realize spatially compact TFMBs with minimal performance loss in confined geometries. This concept substantially enhanced energy and integration density in space-limited platforms. Extending this approach, Meng et al. [212] demonstrated an anode-free TFMB constructed on ultrathin stainless steel (10–75 μm) substrates using a dry patterning process compatible with roll-to-roll manufacturing (Fig. 15a). The fabricated devices offered customizable geometries, high volumetric energy density, and excellent electrochemical reversibility, while maintaining mechanical robustness. Importantly, this process enabled scalable integration via high-throughput patterning, rapid electrochemical screening, and multilayer encapsulation, representing a significant step toward the commercial implementation of flexible TFMBs.

#### Flexible Substrates and Low-Temperature Fabrication

The integration of TFMBs onto flexible substrates, such as polyimide (PI) [213–215], stainless steel foils [148, 216–219], yttria-stabilized zirconia (YSZ) [220], and mica [221, 222], has shown great promise for wearable systems. However, conventional deposition of lithium-containing cathodes (e.g., LCO) requires annealing above 600 °C, which is incompatible with polymer substrates. To overcome this limitation, low-temperature fabrication strategies have been explored to directly deposit amorphous or low-crystalline cathodes onto polymer supports. For instance, Kun et al. [215] reported a MoO_3_/LiPON/Li TFMB fabricated entirely on PI at room temperature via sputtering (Fig. 15b). Despite moderate crystallinity, the device displayed stable capacity retention (125 μAh cm^−2^ μm^−1^ after several hundred cycles) and robust high-rate cycling (62.0 μAh cm^−2^ μm^−1^ at 10 C for 550 cycles). Mechanical stress analyses further revealed that cathode bulk resistance strongly depended on both depth-of-discharge and bending strain (Fig. 15c, d), emphasizing the coupling between mechanical deformation and electrochemical performance in flexible systems. Yet, trade-offs between low-temperature processability and electrochemical quality remain a key bottleneck, requiring further optimization of both materials and fabrication protocols.

#### Transparent TFMBs for Next-Generation Displays

The exponential growth of transparent electronics has stimulated the demand for optically transparent energy storage devices [190]. Benefiting from their submicron-scale architecture, TFMBs can be readily integrated into optoelectronic systems. Two main strategies have emerged: (1) the development of wide-bandgap, optically transparent electrodes; and (2) patterned or semi-transparent thin-film configurations [125, 223]. Choi et al. [224] fabricated sub-stoichiometric SiN_0.85_ thin-film anodes via MS with tunable N_2_ flux, achieving optical transmittance of 55%–78% and reversible capacities above 1000 mAh g^−1^ for 100 cycles. Likewise, transparent LTO and LMO films deposited on transparent conductive oxides (TCOs) exhibited reversible electrochromic behavior during lithiation/delithiation [225]. Pat et al. [226, 227] further realized fully transparent LFP/Li_3_PO_4_/LTO TFMBs, retaining 80% optical transmittance after 100 cycles, thereby validating their practical potential. Alternatively, Oukassi et al. [228] employed photolithography and etching to construct grid-patterned LCO/LiPON/Si TFMBs on glass (Fig. 15e–g), which displayed ~ 60% UV–visible transmittance and stable capacity output. While grid configurations enhance transparency, they inevitably reduce the electrochemically active area. Future strategies should therefore focus on intrinsically transparent electrode/electrolyte systems to simultaneously balance optical and electrochemical performance.

#### TFMBs for Smart Contact Lenses and Biomedical Applications

Smart contact lenses represent a frontier in wearable bioelectronics, requiring miniaturized, conformal, and biocompatible energy storage solutions. Choi et al. [229] obtained a flexible TFMB fabricated on PI using off-axis sputtered LFP films, annealed at a reduced temperature of 400 °C (Fig. 15h–j). The resulting devices achieved an energy density of 70 μWh cm^−2^ and successfully powered an embedded glucose sensor for over 11.7 h under humid conditions. The process demonstrated compatible with CMOS fabrication and scalability to large-area production, facilitating integration with biosensors, microdisplays, and AI-assisted modules for real-time health monitoring and vision enhancement. This work represents a significant milestone toward the practical development of wearable biomedical platforms powered by TFMBs.

## Multifunctional Integrated Energy Harvesting and Storage Systems

To achieve autonomous operation in emerging microsystems, researchers have pursued multifunctional architectures that integrate TFMBs with solar cells, piezoelectric and thermoelectric devices, energy harvesters, and sensors, thereby coupling energy harvesting with on-chip storage. Early demonstrations date back to 2002, when TFMBs were proposed as bypass components in solar panels, replacing conventional diodes to mitigate partial shading losses [230]. Subsequently, millimeter-scale self-powered sensors were developed using two series-connected micro-solar cells and a Cymbet TFMB, regulated by an integrated power management unit [231, 232]. In this system, the TFMB discharged in active mode and recharged under ambient illumination during sleep mode. Furthermore, integration of 16 microscale TFMBs (500 µm × 500 µm) provided 51.3 V output for MEMS devices, serving as an emergency energy buffer during transient shading [233]. These results highlight the potential of direct photovoltaic–TFMB integration for stable, autonomous microsystem power under fluctuating illumination. Beyond photovoltaics, multifunctional TFMB integration has been extended to mechanical and thermal energy harvesting. For instance, piezoelectric vibration harvesters embedded in unmanned aerial vehicle (UAV) wing beams generated ~ 0.0781 mAh at 4.0 V, with the harvested energy stored in TFMBs for localized power delivery [234]. Similarly, RF and thermoelectric energy have been co-harvested and regulated through a DC/DC converter, with the collected energy stored in a 30 mm^2^ on-chip TFMB fabricated via sputtering and thermal evaporation. This device employed a TiOS cathode, LiPON electrolyte, and Li anode, underscoring the versatility of vapor deposition methods in realizing multifunctional integration [235].

Vapor-phase deposition techniques play a central role in enabling such architectures. MS provides dense films with tunable crystallinity, composition, and thickness, supporting multilayer configurations such as LCO/LMO cathodes, LiPON electrolytes, and porous 3D architectures. PLD offers precise stoichiometric transfer, particularly advantageous for complex oxides in piezoelectric and thermoelectric modules. Thermal and e-beam evaporation remain indispensable for Li deposition and high-melting-point materials, respectively, with reactive e-beam evaporation demonstrated for LiPON synthesis [236]. Meanwhile, ALD enables sub-nanometer control and conformal coating, ideal for interface engineering and multifunctional composite construction. In a word, heterogeneous integration of TFMBs with energy harvesting modules through vapor deposition techniques provides a robust pathway to couple energy generation with storage, advancing autonomous, multifunctional microsystems.

## Conclusion and Outlook

This review underscores the central role of vapor deposition techniques in advancing TFMBs, highlighting recent progress in material deposition, interfacial engineering, and structural innovation. Since the first demonstration of an LCO/LiPON/Li prototype in 1996 [237], TFMBs have made steady progress. Yet, compared with the rapid commercialization of LIBs, their industrial translation remains limited by complex fabrication, high costs, and insufficient investment. The accelerating demand for autonomous microsystems in IoT and wearable electronics has reignited interest, but critical bottlenecks in large-scale manufacturability and long-term reliability must still be resolved.

From a materials perspective, cathode development remains less mature than in bulk LIBs. LCO remains dominant due to its stable cycling, but higher-voltage, higher-capacity cathodes are required for next-generation TFMBs, necessitating low-temperature deposition compatible with semiconductor processes. Achieving crystallinity under constrained thermal budgets and developing cost-effective, thermally stable current collectors represent ongoing challenges. On the electrolyte side, amorphous LiPON remains irreplaceable owing to its electrochemical stability and compatibility with Li metal, while amorphous garnet-type thin films (e.g., aLLZO) offer enhanced conductivity but face challenges in film uniformity and interface stability. For anodes, Li metal remains widely adopted, yet concerns regarding thermal instability and integration drive exploration of alternatives such as LTO, which combines structural reversibility with negligible volume change. A quantitative comparison of the state-of-the-art performance metrics across the principal material systems is systematically summarized in Table 6.

**Table 6:** Electrochemical properties of representative thin-film electrode materials

Material	Capacity	Cycle life	Deposition method	References
LCO	58 µAh cm^−2^ µm^−1^	> 75% retention after 1,000 cycles	MS, PLD	[86]
LMO	37 µAh cm^−2^	~ 75% retention after 1,000 cycles	MS, PLD	[69]
LMRO	33.7 µAh cm^−2^ µm^−1^	~ 75% retention after 1,000 cycles	MS	[153]
LNMO	122 mAh g^−1^	> 75% retention after 10,000 cycles	PLD	[151]
LNCM	24.6 µAh cm^−2^ µm^−1^	~ 75% retention after 500 cycles	PLD	[118]
LFP	14.6 µAh cm^−2^ µm^−1^	~ 96% retention after 70 cycles	MS, PLD	[229]
Li_1.2_TiO_0.5_S_2.1_	64 µAh cm^−2^ µm^−1^	> 75% retention after 1,200 cycles	MS	[165]
MoO_3−x_	125 µAh cm^−2^ µm^−1^	~ 85% retention after 550 cycles	MS	[214]
LTO	175 mAh g^−1^	~ 90% retention after 22 cycles	MS, PLD	[144]

From a processing perspective, PVD techniques such as MS, PLD, and e-beam evaporation remain central for producing conformal, compositionally precise films but are constrained by low deposition throughput, inefficient material utilization, and high equipment costs. While reactive e-beam evaporation and parameter optimization can enhance deposition efficiency, hybrid strategies that integrate PVD with CVD or additive manufacturing hold promise for scalable fabrication of complex electrode/electrolyte architectures. The development of roll-to-roll vapor deposition platforms with high throughput and large-area uniformity will be critical to advancing TFMB industrialization.

Interfacial stability remains a key barrier. Side reactions at Li/LiPON and cathode/electrolyte interfaces generate resistive interphases, increasing impedance and polarization. Artificial buffer layers (e.g., Al_2_O_3_ via ALD), structural reconstruction, and thermal treatments have demonstrated effectiveness in suppressing degradation, yet further innovations in nanoscale interfacial engineering are essential. The transition from planar to 3D architectures provides a pathway to enhance areal energy density beyond the typical < 1 mWh cm^−2^ of planar TFMBs, but scalable fabrication of high-aspect-ratio microstructures is still constrained by conformality, cost, and process complexity. Composite designs integrating interconnected electronic/ionic networks may provide a practical route toward scalable high-performance 3D TFMBs.

Future microsystems demand not only high energy and power densities but also multifunctionality, including flexibility, transparency, and integration with energy harvesting units (e.g., photovoltaic, piezoelectric, thermoelectric). Vapor-phase deposition methods are indispensable in constructing these multifunctional systems by enabling conformal buffer layers, sequential stacking of functional films, and thermal management coatings. The synergistic integration of TFMBs with energy harvesters will be pivotal for achieving energy-autonomous microsystems.

Looking forward, convergence with artificial intelligence, including machine learning-driven materials discovery, high-throughput screening, and digital twin modeling, offers transformative opportunities for accelerating TFMB development. These approaches will facilitate predictive design, process optimization, and automated synthesis of advanced architectures, bridging fundamental research with industrial-scale manufacturing. In conclusion, while TFMBs are still at an early stage of technological maturity, continued innovations in material design, interface engineering, and scalable vapor deposition platforms, coupled with emerging AI-driven methodologies, are expected to unlock their potential as indispensable power sources for next-generation microelectronics.

## References

[1] J. Fan, C. Liu, N. Li, L. Yang, X.-G. Yang et al., Wireless transmission of internal hazard signals in Li-ion batteries. Nature **641**(8063), 639–645 (2025). 10.1038/s41586-025-08785-7 40369137 10.1038/s41586-025-08785-7

[2] H. Tang, Y. Yin, Y. Huang, J. Wang, L. Liu et al., Battery-everywhere design based on a cathodeless configuration with high sustainability and energy density. ACS Energy Lett. **6**(5), 1859–1868 (2021). 10.1021/acsenergylett.1c00555

[3] J.H. Pikul, H. Ning, Powering the internet of things. Joule **2**(6), 1036–1038 (2018). 10.1016/j.joule.2018.06.005

[4] H. Hafezi, T.L. Robertson, G.D. Moon, K.-Y. Au-Yeung, M.J. Zdeblick et al., An ingestible sensor for measuring medication adherence. IEEE Trans. Biomed. Eng. **62**(1), 99–109 (2015). 10.1109/TBME.2014.2341272 25069107 10.1109/TBME.2014.2341272

[5] S. Gong, W. Cheng, Toward soft skin-like wearable and implantable energy devices. Adv. Energy Mater. **7**(23), 1700648 (2017). 10.1002/aenm.201700648

[6] P. Birke, W.F. Chu, W. Weppner, Materials for lithium thin-film batteries for application in silicon technology. Solid State Ion. **93**(1–2), 1–15 (1996). 10.1016/S0167-2738(96)00489-4

[7] W. Liu, M.-S. Song, B. Kong, Y. Cui, Flexible and stretchable energy storage: recent advances and future perspectives. Adv. Mater. **29**(1), 1603436 (2017). 10.1002/adma.201603436 10.1002/adma.201603436 28042889

[8] L. Liu, Q. Weng, X. Lu, X. Sun, L. Zhang et al., Advances on microsized on-chip lithium-ion batteries. Small **13**(45), 1701847 (2017). 10.1002/smll.201701847 10.1002/smll.201701847 28960908

[9] C. Cao, Y. Zhong, Z. Shao, Electrolyte engineering for safer lithium-ion batteries: a review. Chin. J. Chem. **41**(9), 1119–1141 (2023). 10.1002/cjoc.202200588

[10] Q. Xia, F. Zan, Q. Zhang, W. Liu, Q. Li, Y. He, J. Hua, J. Liu, J. Xu, J. Wang, C. Wu, H. Xia, All‐solid‐state thin film lithium/lithium‐ion microbatteries for powering the internet of things. Adv. Mater. **35**(2), 2200538 (2023). 10.1002/adma.202200538 10.1002/adma.202200538 35962983

[11] Q. Shi, B. Dong, T. He, Z. Sun, J. Zhu, Z. Zhang, C. Lee, Progress in wearable electronics/photonics—moving toward the era of artificial intelligence and internet of things. InfoMat **2**(6), 1131–1162 (2020). 10.1002/inf2.12122

[12] X. Fan, B. Liu, J. Ding, Y. Deng, X. Han et al., Flexible and wearable power sources for next-generation wearable electronics. Batteries Supercaps **3**(12), 1262–1274 (2020). 10.1002/batt.202000115

[13] Y. Ozawa, Y. Nishitai, N. Ochirkhuyag, N. Usami, M. Kanto, K. Ueno, H. Ota, Liquid metal electrode ink for printable lithium‐ion batteries. Adv. Eng. Mater. **26**(11), 2400529 (2024). 10.1002/adem.202400529

[14] T. Zhang, L. Fu, Controllable chemical vapor deposition growth of two-dimensional heterostructures. Chem. **4**(4), 671–689 (2018). 10.1016/j.chempr.2017.12.006

[15] J. Cai, X. Han, X. Wang, X. Meng, Atomic layer deposition of two-dimensional layered materials: processes, growth mechanisms, and characteristics. Matter **2**(3), 587–630 (2020). 10.1016/j.matt.2019.12.026

[16] S. Moitzheim, B. Put, P.M. Vereecken, Advances in 3D thin-film Li-ion batteries. Adv. Mater. Interfaces **6**(15), 1900805 (2019). 10.1002/admi.201900805

[17] A. Rakita, N. Nikolić, M. Mildner, J. Matiasek, A. Elbe-Bürger, Re-epithelialization and immune cell behaviour in an ex vivo human skin model. Sci. Rep. **10**(1), 1 (2020). 10.1038/s41598-019-56847-4 31913322 10.1038/s41598-019-56847-4 PMC6959339

[18] S. Zheng, X. Shi, P. Das, Z.-S. Wu, X. Bao, The road towards planar microbatteries and micro-supercapacitors: from 2D to 3D device geometries. Adv. Mater. **31**(50), 1900583 (2019). 10.1002/adma.201900583 10.1002/adma.201900583 31222810

[19] X. Pan, X. Hong, L. Xu, Y. Li, M. Yan et al., On-chip micro/nano devices for energy conversion and storage. Nano Today **28**, 100764 (2019). 10.1016/j.nantod.2019.100764

[20] T. Wu, W. Dai, M. Ke, Q. Huang, L. Lu, All-solid-state thin film μ-batteries for microelectronics. Adv. Sci. **8**(19), 2100774 (2021). 10.1002/advs.202100774 10.1002/advs.202100774 PMC8498886 34351691

[21] N.A. Kyeremateng, T. Brousse, D. Pech, Microsupercapacitors as miniaturized energy-storage components for on-chip electronics. Nat. Nanotechnol. **12**(1), 7–15 (2017). 10.1038/nnano.2016.196 27819693 10.1038/nnano.2016.196

[22] J.G. Koomey, H. Scott Matthews, E. Williams, Smart everything: will intelligent systems reduce resource use? Annu. Rev. Environ. Resour. **38**(1), 311–343 (2013). 10.1146/annurev-environ-021512-110549

[23] Z. Wang, Y. Chen, Y. Zhou, J. Ouyang, S. Xu et al., Miniaturized lithium-ion batteries for on-chip energy storage. Nanoscale Adv. **4**(20), 4237–4257 (2022). 10.1039/D2NA00566B 36321148 10.1039/d2na00566b PMC9552904

[24] A. Sumboja, J. Liu, W.G. Zheng, Y. Zong, H. Zhang et al., Electrochemical energy storage devices for wearable technology: a rationale for materials selection and cell design. Chem. Soc. Rev. **47**(15), 5919–5945 (2018). 10.1039/C8CS00237A 29947399 10.1039/c8cs00237a

[25] Y. Lu, K. Jiang, D. Chen, G. Shen, Wearable sweat monitoring system with integrated micro-supercapacitors. Nano Energy **58**, 624–632 (2019). 10.1016/j.nanoen.2019.01.084

[26] G. Sun, X. Jin, H. Yang, J. Gao, L. Qu, An aqueous Zn–MnO_2_ rechargeable microbattery. J. Mater. Chem. A **6**(23), 10926–10931 (2018). 10.1039/C8TA02747A

[27] K. Chen, Y. Sun, X. Zhang, J. Liu, H. Xie, A self-healing and nonflammable cross-linked network polymer electrolyte with the combination of hydrogen bonds and dynamic disulfide bonds for lithium metal batteries. Energy Environ. Mater. **6**, e12568 (2023). 10.1002/eem2.12568

[28] J. Gao, Y. Tian, L. Ni, B. Wang, K. Zou et al., Robust cross-linked Na_3_V_2_(PO_4_)_2_F_3_ full sodium-ion batteries. Energy Environ. Mater. **7**, e12485 (2024). 10.1002/eem2.12485

[29] B. Wang, C. Guan, Q. Zhou, Y. Wang, Y. Zhu et al., Screening anionic groups within zwitterionic additives for eliminating hydrogen evolution and dendrites in aqueous Zinc ion batteries. Nano-Micro Lett. **17**(1), 314 (2015). 10.1007/s40820-025-01826-w 10.1007/s40820-025-01826-w PMC12202267 40569324

[30] C.M. Julien, A. Mauger, Pulsed laser deposited films for microbatteries. Coatings **9**(6), 386 (2019). 10.3390/coatings9060386

[31] L. Indrizzi, N. Ohannessian, D. Pergolesi, T. Lippert, E. Gilardi, Pulsed laser deposition as a tool for the development of all solid‐state microbatteries. Helv. Chim. Acta **104**(2), e2000203 (2021). 10.1002/hlca.202000203

[32] M. Fenech, N. Sharma, Pulsed laser deposition-based thin film microbatteries. Chem. Asian J. **15**(12), 1829–1847 (2020). 10.1002/asia.202000384 32338830 10.1002/asia.202000384

[33] X. Liang, F. Tan, F. Wei, J. Du, Research progress of all solid-state thin film lithium battery. IOP Conf. Ser. Earth Environ. Sci. **218**, 012138 (2019). 10.1088/1755-1315/218/1/012138

[34] J. Lin, L. Lin, S. Qu, D. Deng, Y. Wu, X. Yan, Q. Xie, L. Wang, D. Peng, Promising electrode and electrolyte materials for high‐energy‐density thin‐film lithium batteries. Energy Environ. Mater. **5**(1), 133–156 (2022). 10.1002/eem2.12202

[35] S.A. Mahyoub, A. Farid, M.Z. Azeem, D.A.A. Fadhil, F.A. Qaraah, Q.A. Drmosh, Physical vapor deposition techniques for CO_2_ electroreduction: a review. Small Struct. **6**(5), 2400501 (2025). 10.1002/sstr.202400501

[36] S. Lobe, A. Bauer, S. Uhlenbruck, D. Fattakhova‐Rohlfing, Physical vapor deposition in solid‐state battery development: from materials to devices. Adv. Sci. **8**(11), 2002044 (2021). 10.1002/advs.202002044 10.1002/advs.202002044 PMC8188201 34105301

[37] E.C. Nwanna, S. Bitire, P.E. Imoisili, T. Jen, An overview of the application of atomic layer deposition process for lithium-ion based batteries. Int. J. Energy Res. **46**(8), 10499–10521 (2022). 10.1002/er.7941

[38] M. Curcio, R. Teghil, A. De Bonis, Thin-film microbattery fabrication by PLD: a comprehensive mini-review. Front. Coat. Dyes Interface Eng. **2**, 1401391 (2024). 10.3389/frcdi.2024.1401391

[39] Y. Yao, X. Jiao, X. Xu, S. Xiong, Z. Song, Y. Liu, Prospective of magnetron sputtering for interface design in rechargeable lithium batteries. Adv. Energy Mater. **14**(47), 2403117 (2024). 10.1002/aenm.202403117

[40] A.S. Westover, R. Sahore, K.L. Browning, S. Kalnaus, Thermal evaporation of thin Li films. J. Vac. Sci. Technol. B **43**(2), 022401 (2025). 10.1116/6.0004174

[41] D. Sun, S. Tian, C. Yin, F. Chen, J. Xie, C. Huang, C. Li, Thin film deposition techniques in surface engineering strategies for advanced lithium-ion batteries. Coatings **13**(3), 505 (2023). 10.3390/coatings13030505

[42] M. Beidaghi, Y. Gogotsi, Capacitive energy storage in micro-scale devices: recent advances in design and fabrication of micro-supercapacitors. Energy Environ. Sci. **7**(3), 867–884 (2014). 10.1039/c3ee43526a

[43] R. Karthikeyan, C.T. Cherian, R.P. Fernandes, Review: rf magnetron sputtering, a promising synthesis route for scalable production of thin-film batteries. J. Mater. Sci. **60**(21), 8569–8601 (2025). 10.1007/s10853-025-10929-z

[44] W. Xiong, Q. Xia, H. Xia, Three-dimensional self-supported metal oxides as cathodes for microbatteries. Funct. Mater. Lett. **07**(05), 1430003 (2014). 10.1142/s1793604714300035

[45] A. Mauger, M. Armand, C.M. Julien, K. Zaghib, Challenges and issues facing lithium metal for solid-state rechargeable batteries. J. Power. Sources **353**, 333–342 (2017). 10.1016/j.jpowsour.2017.04.018

[46] V. Patil, V. Patil, D. Wook Shin, J.-W. Choi, D.-S. Paik et al., Issue and challenges facing rechargeable thin film lithium batteries. Mater. Res. Bull. **43**(8−9), 1913–1942 (2008). 10.1016/j.materresbull.2007.08.031

[47] E. Pomerantseva, F. Bonaccorso, X. Feng, Y. Cui, Y. Gogotsi, Energy storage: the future enabled by nanomaterials. Science **366**(6468), eaan8285 (2019). 10.1126/science.aan8285 31753970 10.1126/science.aan8285

[48] X. Zhang, M. Lok, T. Tong, S.K. Lee, B. Reagen et al., A fully integrated battery-powered system-on-chip in 40-nm CMOS for closed-loop control of insect-scale pico-aerial vehicle. IEEE J. Solid-State Circuits **52**(9), 2374–2387 (2017). 10.1109/jssc.2017.2705170

[49] W.C. West, J.F. Whitacre, V. White, B.V. Ratnakumar, Fabrication and testing of all solid-state microscale lithium batteries for microspacecraft applications. J. Micromech. Microeng. **12**(1), 58–62 (2001). 10.1088/0960-1317/12/1/309

[50] C.C. Liang, P. Bro, A high‐voltage, solid‐state battery system: i. design considerations. J. Electrochem. Soc. **116**(9), 1322 (1969). 10.1149/1.2412312

[51] C.-L. Li, Z.-W. Fu, All-solid-state rechargeable thin film lithium batteries with Li_x_Mn_2_O_4_ and Li_x_Mn_2_O_4_–0.5ZrO_2_ cathodes. Electrochim. Acta **52**(20), 6155–6164 (2007). 10.1016/j.electacta.2007.04.012

[52] G. Vardar, W.J. Bowman, Q. Lu, J. Wang, R.J. Chater et al., Structure, chemistry, and charge transfer resistance of the interface between Li_7_La_3_Zr_2_O_12_ electrolyte and LiCoO_2_ cathode. Chem. Mater. **30**(18), 6259–6276 (2018). 10.1021/acs.chemmater.8b01713

[53] S. Tintignac, R. Baddour-Hadjean, J.P. Pereira-Ramos, R. Salot, High rate bias sputtered LiCoO_2_ thinfilms as positive electrode for all-solid-state lithium microbatteries. Electrochim. Acta **146**, 472–476 (2014). 10.1016/j.electacta.2014.09.084

[54] A. Kumatani, S. Shiraki, Y. Takagi, T. Suzuki, T. Ohsawa, X. Gao, Y. Ikuhara, T. Hitosugi, Epitaxial growth of Li_4_Ti_5_O_12_ thin films using rf magnetron sputtering. Jpn. J. Appl. Phys. **53**(5), 058001 (2014). 10.7567/jjap.53.058001

[55] J. Feng, B. Yan, M.O. Lai, L. Li, Design and fabrication of an all-solid-state thin-film Li-ion microbattery with amorphous TiO_2_ as the anode. Energy Technol. **2**(4), 397–400 (2014). 10.1002/ente.201300173

[56] G. El Omari, K. El Kindoussy, M. Aqil, M. Dahbi, J. Alami, M. Makha, Advances in physical vapor deposited silicon/carbon based anode materials for Li-ion batteries. Heliyon **10**(9), e30431 (2024). 10.1016/j.heliyon.2024.e30431 38726107 10.1016/j.heliyon.2024.e30431 PMC11079090

[57] L. Chai, X. Wang, C. Bi, B. Su, C. Zhang, X. Li, W. Xue, Lifetime optimization of amorphous silicon thin-film anodes for lithium-ion batteries. ACS Appl. Energy Mater. **6**(16), 8388–8396 (2023). 10.1021/acsaem.3c01127

[58] B. Put, P.M. Vereecken, A. Stesmans, On the chemistry and electrochemistry of LiPON breakdown. J. Mater. Chem. A **6**(11), 4848–4859 (2018). 10.1039/c7ta07928A

[59] Z. Warecki, V.C. Ferrari, D.A. Robinson, J.D. Sugar, J. Lee, A.V. Ievlev, N.S. Kim, D.M. Stewart, S.B. Lee, P. Albertus, G. Rubloff, A.A. Talin, Simultaneous solid electrolyte deposition and cathode lithiation for thin film batteries and lithium iontronic devices. ACS Energy Lett. **9**(5), 2065–2074 (2024). 10.1021/acsenergylett.4c00674

[60] B. Ke, S. Cheng, C. Zhang, W. Li, J. Zhang, R. Deng, J. Lin, Q. Xie, B. Qu, Li. Qiao, D.-L. Peng, X. Wang, Low-temperature flexible integration of all-solid-state thin-film lithium batteries enabled by spin-coating electrode architecture. Adv. Energy Mater. **14**(12), 2303757 (2024). 10.1002/aenm.202303757

[61] B. Ke, C. Zhang, S. Cheng, W. Li, R. Deng, H. Zhang, J. Lin, Q. Xie, B. Qu, D.-L. Peng, X. Wang, Tape-casting electrode architecture permits low-temperature manufacturing of all-solid-state thin-film microbatteries. Interdiscip. Mater. **3**(4), 621–631 (2024). 10.1002/idm2.12174

[62] S. Lobe, C. Dellen, M. Finsterbusch, H.-G. Gehrke, D. Sebold et al., Radio frequency magnetron sputtering of Li_7_La_3_Zr_2_O_12_ thin films for solid-state batteries. J. Power. Sources **307**, 684–689 (2016). 10.1016/j.jpowsour.2015.12.054

[63] J. Feng, B. Yan, J. Liu, M. Lai, L. Li, All solid state lithium ion rechargeable batteries using NASICON structured electrolyte. Mater. Technol. **28**(5), 276–279 (2013). 10.1179/1753555713Y.0000000085

[64] J.B. Bates, N.J. Dudney, G.R. Gruzalski, R.A. Zuhr, A. Choudhury, C.F. Luck, J.D. Robertson, Fabrication and characterization of amorphous lithium electrolyte thin films and rechargeable thin-film batteries. J. Power. Sources **43**(1−3), 103–110 (1993). 10.1016/0378-7753(93)80106-Y

[65] C. Erinmwingbovo, V. Siller, M. Nuñez, R. Trócoli, D. Brogioli, A. Morata, F. La Mantia, Dynamic impedance spectroscopy of LiMn_2_O_4_ thin films made by multi-layer pulsed laser deposition. Electrochim. Acta **331**, 135385 (2020). 10.1016/j.electacta.2019.135385

[66] T. Ohnishi, K. Takada, Sputter-deposited amorphous Li_3_PO_4_ solid electrolyte films. ACS Omega **7**(24), 21199–21206 (2022). 10.1021/acsomega.2c02104 35755344 10.1021/acsomega.2c02104 PMC9219063

[67] G. Tan, F. Wu, L. Li, Y. Liu, R. Chen, Magnetron sputtering preparation of nitrogen-incorporated lithium-aluminum-titanium phosphate based thin film electrolytes for all-solid-state lithium ion batteries. J. Phys. Chem. C **116**(5), 3817–3826 (2012). 10.1021/jp207120s

[68] X. Yang, Y. Shen, L. Zhang, Y. Li, Recent advances in the application of magnetron sputtering for lithium metal batteries. J. Mater. Chem. A **13**(39), 33104–33135 (2025). 10.1039/d5ta03766b

[69] Q. Xia, Q. Zhang, S. Sun, F. Hussain, C. Zhang, X. Zhu, F. Meng, K. Liu, H. Geng, J. Xu, F. Zan, P. Wang, L. Gu, H. Xia, Tunnel intergrowth Li_x_MnO_2_ nanosheet arrays as 3D cathode for high‐performance all‐solid‐state thin film lithium microbatteries. Adv. Mater. **33**(5), 2003524 (2021). 10.1002/adma.202003524 10.1002/adma.202003524 33336535

[70] K. Yuan, M. Xie, W. Dong, P. Zhao, J. Pan et al., High-speed and one-step deposition of a LiCoO_2_ thin-film electrode by a high-repetition 1064 nm ND:YAG fiber laser. ACS Appl. Energy Mater. **5**(12), 15483–15490 (2022). 10.1021/acsaem.2c02826

[71] K. Hikima, K. Shimizu, H. Kiuchi, Y. Hinuma, K. Suzuki et al., Reaction mechanism of Li_2_MnO_3_ electrodes in an all-solid-state thin-film battery analyzed by operando hard X-ray photoelectron spectroscopy. J. Am. Chem. Soc. **144**(1), 236–247 (2022). 10.1021/jacs.1c09087 34957828 10.1021/jacs.1c09087

[72] E. Moazzen, J. Mujtaba, B. Buchholz, D. Isheim, N.S. Luu, D. Rowell, X. Hu, T. Ha, M.C. Hersam, S.A. Barnett, Atom probe tomography of the LiFePO_4_-electrolyte interface enabled by thin film electrodes. J. Electrochem. Soc. **171**(7), 070527 (2024). 10.1149/1945-7111/ad5f21

[73] K. Wei, J. Qiu, Y. Zhao, S. Ma, Y. Wei, H. Li, C. Zeng, Y. Cui, Tunable oxygen vacancies in MoO_3_ lattice with improved electrochemical performance for Li-ion battery thin film cathode. Ceram. Int. **49**(13), 21729–21736 (2023). 10.1016/j.ceramint.2023.03.313

[74] P. Schichtel, M. Geiß, T. Leichtweiß, J. Sann, D.A. Weber, J. Janek, On the impedance and phase transition of thin film all-solid-state batteries based on the Li_4_Ti_5_O_12_ system. J. Power. Sources **360**, 593–604 (2017). 10.1016/j.jpowsour.2017.06.044

[75] K. Senevirathne, C.S. Day, M.D. Gross, A. Lachgar, N.A.W. Holzwarth, A new crystalline LiPON electrolyte: synthesis, properties, and electronic structure. Solid State Ion. **233**, 95–101 (2013). 10.1016/j.ssi.2012.12.013

[76] W.C. West, Z.D. Hood, S.P. Adhikari, C. Liang, A. Lachgar et al., Reduction of charge-transfer resistance at the solid electrolyte-electrode interface by pulsed laser deposition of films from a crystalline Li_2_PO_2_N source. J. Power. Sources **312**, 116–122 (2016). 10.1016/j.jpowsour.2016.02.034

[77] N. Kuwata, N. Iwagami, Y. Matsuda, Y. Tanji, J. Kawamura, Thin film batteries with Li_3_PO_4_ solid electrolyte fabricated by pulsed laser deposition. ECS Trans. **16**(26), 53–60 (2009). 10.1149/1.3111821

[78] A.V. Morozov, H. Paik, A.O. Boev, D.A. Aksyonov, S.A. Lipovskikh et al., Thermodynamics as a driving factor of LiCoO_2_ grain growth on nanocrystalline Ta-LLZO thin films for all-solid-state batteries. ACS Appl. Mater. Interfaces **14**(35), 39907–39916 (2022). 10.1021/acsami.2c07176 36007961 10.1021/acsami.2c07176

[79] P.M. Gonzalez Puente, S. Song, S. Cao, L.Z. Rannalter, Z. Pan et al., Garnet-type solid electrolyte: advances of ionic transport performance and its application in all-solid-state batteries. J. Adv. Ceram. **10**(5), 933–972 (2021). 10.1007/s40145-021-0489-7

[80] I. Garbayo, M. Struzik, W.J. Bowman, R. Pfenninger, E. Stilp, J.L.M. Rupp, Glass‐type polyamorphism in Li‐garnet thin film solid state battery conductors. Adv. Energy Mater. **8**(12), 1702265 (2018). 10.1002/aenm.201702265

[81] M. Saccoccio, J. Yu, Z. Lu, S.C.T. Kwok, J. Wang et al., Low temperature pulsed laser deposition of garnet Li_6.4_La_3_Zr_1.4_Ta_0.6_O_12_ films as all solid-state lithium battery electrolytes. J. Power. Sources **365**, 43–52 (2017). 10.1016/j.jpowsour.2017.08.020

[82] V. Siller, A. Morata, M.N. Eroles, R. Arenal, J.C. Gonzalez-Rosillo et al., High performance LATP thin film electrolytes for all-solid-state microbattery applications. J. Mater. Chem. A **9**(33), 17760–17769 (2021). 10.1039/D1TA02991F

[83] J. Wei, D. Ogawa, T. Fukumura, Y. Hirose, T. Hasegawa, Epitaxial strain-controlled ionic conductivity in Li-ion solid electrolyte Li_0.33_La_0.56_TiO_3_ thin films. Cryst. Growth Des. **15**(5), 2187–2191 (2015). 10.1021/cg501834s

[84] M. Curcio, A.D. Bonis, S. Brutti, A. Santagata, R. Teghil, Pulsed laser deposition of thin films of TiO_2_ for Li-ion batteries. Appl. Surf. Sci. Adv. **4**, 100090 (2021). 10.1016/j.apsadv.2021.100090

[85] P. Albertus, S. Babinec, S. Litzelman, A. Newman, Status and challenges in enabling the lithium metal electrode for high-energy and low-cost rechargeable batteries. Nat. Energy **3**(1), 16–21 (2018). 10.1038/s41560-017-0047-2

[86] B.J. Neudecker, N.J. Dudney, J.B. Bates, “Lithium-Free” thin-film battery with in situ plated Li anode. J. Electrochem. Soc. **147**(2), 517 (2000). 10.1149/1.1393226

[87] J. Zheng, L. Ye, M. He, D. He, Y. Huang et al., Electrical and optical properties of amorphous silicon carbide thin films prepared by e-beam evaporation at room temperature. J. Non-Cryst. Solids **576**, 121233 (2022). 10.1016/j.jnoncrysol.2021.121233

[88] P. Jayaraman, H. Annal Therese, Flexible interdigitated symmetric solid-state micro-supercapacitors with higher energy density for wearable electronics. J. Power. Sources **581**, 233489 (2023). 10.1016/j.jpowsour.2023.233489

[89] J. Li, L. Li, W. Chen, Q. Yi, G. Zou, Room-temperature processed high-quality SnO_2_ films by oxygen plasma activated e-beam evaporation. Nanotechnology **32**(2), 025606 (2020). 10.1088/1361-6528/abbce9 10.1088/1361-6528/abbce9 32998117

[90] Q. Chen, H. Li, M.L. Meyerson, R. Rodriguez, K. Kawashima et al., Li−Zn overlayer to facilitate uniform lithium deposition for lithium metal batteries. ACS Appl. Mater. Interfaces **13**(8), 9985–9993 (2021). 10.1021/acsami.0c21195 33591714 10.1021/acsami.0c21195

[91] C.-L. Li, B. Zhang, Z.-W. Fu, Physical and electrochemical characterization of amorphous lithium lanthanum titanate solid electrolyte thin-film fabricated by e-beam evaporation. Thin Solid Films **515**(4), 1886–1892 (2006). 10.1016/j.tsf.2006.07.026

[92] A. Baptista, F.J.G. Silva, J. Porteiro, J.L. Míguez, G. Pinto et al., On the physical vapour deposition (PVD): evolution of magnetron sputtering processes for industrial applications. Proc. Manuf. **17**, 746–757 (2018). 10.1016/j.promfg.2018.10.125

[93] M. Surendran, S. Singh, H. Chen, C. Wu, A. Avishai et al., A hybrid pulsed laser deposition approach to grow thin films of chalcogenides. Adv. Mater. **36**(19), 2312620 (2024). 10.1002/adma.202312620 10.1002/adma.202312620 38288906

[94] S. Karimzadeh, B. Safaei, C. Yuan, T.-C. Jen, Emerging atomic layer deposition for the development of high-performance lithium-ion batteries. Electrochem. Energy Rev. **6**(1), 24 (2023). 10.1007/s41918-023-00192-8

[95] H. Zhang, J.-S. Cherng, Q. Chen, Recent progress on high power impulse magnetron sputtering (HiPIMS): the challenges and applications in fabricating VO_2_ thin film. AIP Adv. **9**(3), 035242 (2019). 10.1063/1.5084031

[96] B. Lacroix, A.J. Santos, S. Hurand, A. Corvisier, F. Paumier, T. Girardeau, F. Maudet, C. Dupeyrat, R. García, F.M. Morales, Nanostructure and physical properties control of indium tin oxide films prepared at room temperature through ion beam sputtering deposition at oblique angles. J. Phys. Chem. C **123**(22), 14036–14046 (2019). 10.1021/acs.jpcc.9b02885

[97] H. He, L. Wang, M. Al-Abbasi, C. Cao, H. Li et al., Interface engineering on constructing physical and chemical stable solid-state electrolyte toward practical lithium batteries. Energy Environ. Mater. **7**(4), e12699 (2024). 10.1002/eem2.12699

[98] R.B. Tokas, S. Jena, C. Prathap, S. Thakur, K. Divakar Rao et al., Study of reactive electron beam deposited tantalum penta oxide thin films with spectroscopic ellipsometry and atomic force microscopy. Appl. Surf. Sci. Adv. **18**, 100480 (2023). 10.1016/j.apsadv.2023.100480

[99] J.F.M. Oudenhoven, T. van Dongen, Ra.H. Niessen, M.H.J.M. de Croon, P.H.L. Notten, Low-pressure chemical vapor deposition of LiCoO_2_ thin films: a systematic investigation of the deposition parameters. J. Electrochem. Soc. **156**(5), D169 (2009). 10.1149/1.3082374

[100] L. Meda, E.E. Maxie, LiPON thin films grown by plasma-enhanced metalorganic chemical vapor deposition in a N_2_-H_2_-Ar gas mixture. Thin Solid Films **520**(6), 1799–1803 (2012). 10.1016/j.tsf.2011.08.091

[101] J. Xie, J.F.M. Oudenhoven, P.-P.R.M.L. Harks, D. Li, P.H.L. Notten, Chemical vapor deposition of lithium phosphate thin-films for 3D all-solid-state Li-ion batteries. J. Electrochem. Soc. **162**(3), A249 (2014). 10.1149/2.0091503jes

[102] K. Tadanaga, A. Yamaguchi, A. Hayashi, M. Tatsumisago, J. Mosa et al., Preparation of Li_4_Ti_5_O_12_ electrode thin films by a mist CVD process with aqueous precursor solution. J. Asian Ceram. Soc. **3**(1), 88–91 (2015). 10.1016/j.jascer.2014.11.003

[103] J. Xie, P.-P.R.M.L. Harks, D. Li, L.H.J. Raijmakers, P.H.L. Notten, Planar and 3D deposition of Li_4_Ti_5_O_12_ thin film electrodes by MOCVD. Solid State Ionics **287**, 83–88 (2016). 10.1016/j.ssi.2016.02.004

[104] R. Deng, B. Ke, Y. Xie, S. Cheng, C. Zhang, H. Zhang, B. Lu, X. Wang, All-solid-state thin-film lithium-sulfur batteries. Nano-Micro Lett. **15**(1), 73 (2023). 10.1007/s40820-023-01064-y 10.1007/s40820-023-01064-y PMC10043110 36971905

[105] M. Balaish, K.J. Kim, H. Chu, Y. Zhu, J. Carlos Gonzalez-Rosillo et al., Emerging processing guidelines for solid electrolytes in the era of oxide-based solid-state batteriest. Chem. Soc. Rev. **54**(19), 8925–9007 (2025). 10.1039/d5cs00358j 40905251 10.1039/d5cs00358j

[106] M. Madadi, M. Heikkinen, A. Philip, M. Karppinen, Conformal high-aspect-ratio solid electrolyte thin films for Li-ion batteries by atomic layer deposition. ACS Appl. Electron. Mater. **6**(63), 1574–1580 (2024). 10.1021/acsaelm.3c01565 38558950 10.1021/acsaelm.3c01565 PMC10976887

[107] Y. Wang, Z. Feng, X. Wang, M. Meng, Y. Sun, M. Jing, H. Liu, F. Lu, W. Wang, Y. Cheng, X. Huang, F. Luo, X. Sun, H. Dong, Inhibitory property of lithium phosphorus oxynitride surface grown by atomic layer deposition. Surfaces and Interfaces **33**, 102280 (2022). 10.1016/j.surfin.2022.102280

[108] J. Speulmanns, S. Bonhardt, W. Weinreich, P. Adelhelm, Interface-engineered atomic layer deposition of 3D Li_4_Ti_5_O_12_ for high-capacity lithium-ion 3D thin-film batteries. Small **20**(42), 2403453 (2024). 10.1002/smll.202403453 10.1002/smll.202403453 38850189

[109] X. Han, Y. Gong, K. Fu, X. He, G.T. Hitz, J. Dai, A. Pearse, B. Liu, H. Wang, G. Rubloff, Y. Mo, V. Thangadurai, E.D. Wachsman, L. Hu, Negating interfacial impedance in garnet-based solid-state Li metal batteries. Nat. Mater. **16**(5), 572–579 (2017). 10.1038/nmat4821 27992420 10.1038/nmat4821

[110] J.B. Bates, N.J. Dudney, G.R. Gruzalski, R.A. Zuhr, A. Choudhury et al., Electrical properties of amorphous lithium electrolyte thin films. Solid State Ion. **53**, 647–654 (1992). 10.1016/0167-2738(92)90442-R

[111] H. Nakazawa, K. Sano, T. Abe, M. Baba, N. Kumagai, Charge-discharge characteristics of all-solid-state thin-filmed lithium-ion batteries using amorphous Nb_2_O_5_ negative electrodes. J. Power. Sources **174**(2), 838–842 (2007). 10.1016/j.jpowsour.2007.06.226

[112] M. Haruta, S. Shiraki, T. Ohsawa, T. Suzuki, A. Kumatani et al., Preparation and in-situ characterization of well-defined solid electrolyte/electrode interfaces in thin-film lithium batteries. Solid State Ion. **285**, 118–121 (2016). 10.1016/j.ssi.2015.06.007

[113] J. Trask, A. Anapolsky, B. Cardozo, E. Januar, K. Kumar et al., Optimization of 10-μm, sputtered, LiCoO_2_ cathodes to enable higher energy density solid state batteries. J. Power. Sources **350**, 56–64 (2017). 10.1016/j.jpowsour.2017.03.017

[114] H.-S. Kim, Y. Oh, K.H. Kang, J.H. Kim, J. Kim et al., Characterization of sputter-deposited LiCoO_2_ thin film grown on NASICON-type electrolyte for application in all-solid-state rechargeable lithium battery. ACS Appl. Mater. Interfaces **9**(19), 16063–16070 (2017). 10.1021/acsami.6b15305 28443657 10.1021/acsami.6b15305

[115] S. Sun, Z. Han, W. Liu, Q. Xia, L. Xue et al., Lattice pinning in MoO_3_ via coherent interface with stabilized Li^+^ intercalation. Nat. Commun. **14**(1), 6662 (2023). 10.1038/s41467-023-42335-x 37863930 10.1038/s41467-023-42335-x PMC10589268

[116] M. Fehse, R. Trócoli, E. Ventosa, E. Hernández, A. Sepúlveda et al., Ultrafast dischargeable LiMn_2_O_4_ thin-film electrodes with pseudocapacitive properties for microbatteries. ACS Appl. Mater. Interfaces **9**(6), 5295–5301 (2017). 10.1021/acsami.6b15258 28102072 10.1021/acsami.6b15258

[117] Z. Qi, J. Jian, J. Huang, J. Tang, H. Wang et al., LiNi_0.5_Mn_0.3_Co_0.2_O_2_/Au nanocomposite thin film cathode with enhanced electrochemical properties. Nano Energy **46**, 290–296 (2018). 10.1016/j.nanoen.2018.02.011

[118] J. Deng, L. Xib, L. Wang, Z. Wang, C.Y. Chung et al., Electrochemical performance of LiNi_1/3_Co_1/3_Mn_1/3_O_2_ thin film electrodes prepared by pulsed laser deposition. J. Power. Sources **217**, 491–497 (2012). 10.1016/j.jpowsour.2012.06.006

[119] C. Jacob, T. Lynch, A. Chen, J. Jian, H. Wang, Highly textured Li(Ni_0.5_Mn_0.3_Co_0.2_)O_2_ thin films on stainless steel as cathode for lithium-ion battery. J. Power. Sources **241**, 410–414 (2013). 10.1016/j.jpowsour.2013.04.140

[120] A. Bünting, S. Uhlenbruck, D. Sebold, H.P. Buchkremer, R. Vaßen, Three-dimensional, fibrous lithium iron phosphate structures deposited by magnetron sputtering. ACS Appl. Mater. Interfaces **7**(40), 22594–22600 (2015). 10.1021/acsami.5b07090 26381359 10.1021/acsami.5b07090

[121] V.A. Sugiawati, F. Vacandio, C. Perrin-Pellegrino, A. Galeyeva, A.P. Kurbatov et al., &lt;article-title update="added"&gt;Sputtered porous Li-Fe-P-O film cathodes prepared by radio frequency sputtering for Li-ion microbatteries. Sci. Rep. **9**(1), 11172 (2019). 10.1038/s41598-019-47464-2 31371758 10.1038/s41598-019-47464-2 PMC6671976

[122] R.M. Ugalde-Vázquez, F. Ambriz-Vargas, F. Morales-Morales, N. Hernández-Sebastián, A. Benítez-Lara et al., Effect of argon sputtering pressure on the electrochemical performance of LiFePO_4_ cathode. J. Eur. Ceram. Soc. **43**(2), 407–418 (2023). 10.1016/j.jeurceramsoc.2022.10.030

[123] L. Wang, J. Qiu, X. Wang, L. Chen, G. Cao et al., Insights for understanding multiscale degradation of LiFePO_4_ cathodes. eScience **2**(2), 125–137 (2022). 10.1016/j.esci.2022.03.006

[124] D. Kang, K. Ito, K. Shimizu, K. Watanabe, N. Matsui et al., Fabrication and high-temperature electrochemical stability of LiFePO_4_ cathode/Li_3_PO_4_ electrolyte interface. Electrochemistry **92**(3), 037008 (2024). 10.5796/electrochemistry.24-00017

[125] H. Lee, S. Kim, N.S. Parmar, J.-H. Song, K. Chung et al., Carbon-free Mn-doped LiFePO_4_ cathode for highly transparent thin-film batteries. J. Power. Sources **434**, 226713 (2019). 10.1016/j.jpowsour.2019.226713

[126] J.B. Bates, N.J. Dudney, B.J. Neudecker, F.X. Hart, H.P. Jun et al., Preferred orientation of polycrystalline LiCoO_2_ films. J. Electrochem. Soc. **147**(1), 59–70 (2000). 10.1149/1.1393157

[127] Y. Yoon, C. Park, J. Kim, D. Shin, Lattice orientation control of lithium cobalt oxide. J. Power. Sources **226**, 186–190 (2013). 10.1016/j.jpowsour.2012.10.094

[128] H. Xia, L. Lu, Texture effect on the electrochemical properties of LiCoO_2_ thin films prepared by PLD. Electrochim. Acta **52**(24), 7014–7021 (2007). 10.1016/j.electacta.2007.05.019

[129] J.F. Whitacre, W.C. West, E. Brandon, B.V. Ratnakumar, Crystallographically oriented thin-film nanocrystalline cathode layers prepared without exceeding 300 °C. J. Electrochem. Soc. **148**(10), A1078 (2001). 10.1149/1.1400119

[130] Q. Xia, S. Sun, J. Xu, F. Zan, J. Yue, Q. Zhang, L. Gu, H. Xia, Self‐standing 3D cathodes for all‐solid‐state thin film lithium batteries with improved interface kinetics. Small **14**(52), 1804149 (2018). 10.1002/smll.201804149 10.1002/smll.201804149 30467972

[131] W. Wu, J. Duan, J. Wen, Y. Chen, X. Liu et al., A writable lithium metal ink. Sci. China Chem. **63**(10), 1483–1489 (2020). 10.1007/s11426-020-9810-1

[132] P.R. Abel, A.M. Chockla, Y.-M. Lin, V.C. Holmberg, J.T. Harris, B.A. Korgel, A. Heller, C.B. Mullins, Nanostructured Si_(1−x)_Ge_x_ for tunable thin film lithium-ion battery anodes. ACS Nano **7**(3), 2249–2257 (2013). 10.1021/nn3053632 23432354 10.1021/nn3053632

[133] B.D. Polat, O.L. Eryilmaz, O. Keleş, A. Erdemir, K. Amine, Compositionally graded SiCu thin film anode by magnetron sputtering for lithium ion battery. Thin Solid Films **596**, 190–197 (2015). 10.1016/j.tsf.2015.09.085

[134] M. Kotobuki, K. Hoshina, K. Kanamura, Electrochemical properties of thin TiO_2_ electrode on Li_1+x_Al_x_Ge_2−x_(PO_4_)_3_ solid electrolyte. Solid State Ionics **198**(1), 22–25 (2011). 10.1016/j.ssi.2011.07.003

[135] J. Haetge, P. Hartmann, K. Brezesinski, J. Janek, T. Brezesinski, Ordered large-pore mesoporous Li_4_Ti_5_O_12_ spinel thin film electrodes with nanocrystalline framework for high rate rechargeable lithium batteries: relationships among charge storage, electrical conductivity, and nanoscale structure. Chem. Mater. **23**(19), 4384–4393 (2011). 10.1021/cm202185y

[136] J. Deng, Z. Lu, C.Y. Chung, X. Han, Z. Wang et al., Electrochemical performance and kinetic behavior of lithium ion in Li_4_Ti_5_O_12_ thin film electrodes. Appl. Surf. Sci. **314**, 936–941 (2014). 10.1016/j.apsusc.2014.06.162

[137] F. Wunde, F. Berkemeier, G. Schmitz, Lithium diffusion in sputter-deposited Li_4_Ti_5_O_12_ thin films. J. Power. Sources **215**, 109–115 (2012). 10.1016/j.jpowsour.2012.04.102

[138] H. Wu, Y. Cui, Designing nanostructured si anodes for high energy lithium ion batteries. Nano Today **7**(5), 414–429 (2012). 10.1016/j.nantod.2012.08.004

[139] J.P. Maranchi, A.F. Hepp, P.N. Kumta, High capacity, reversible silicon thin-film anodes for lithium-ion batteries. Electrochem. Solid-State Lett. **6**(9), A198–A201 (2003). 10.1149/1.1596918

[140] D. Cheng, T.A. Wynn, X. Wang, S. Wang, M. Zhang et al., Unveiling the stable nature of the solid electrolyte interphase between lithium metal and LiPON via cryogenic electron microscopy. Joule **4**(11), 2484–2500 (2020). 10.1016/j.joule.2020.08.013

[141] J. Lin, J. Guo, C. Liu, H. Guo, Ultrahigh-performance Cu_2_ZnSnS_4_ thin film and its application in microscale thin-film lithium-ion battery: comparison with SnO_2_. ACS Appl. Mater. Interfaces **8**(50), 34372–34378 (2016). 10.1021/acsami.6b10730 27936547 10.1021/acsami.6b10730

[142] D.M. Stewart, A.J. Pearse, N.S. Kim, E.J. Fuller, A.A. Talin et al., Tin oxynitride anodes by atomic layer deposition for solid-state batteries. Chem. Mater. **30**(8), 2526–2534 (2018). 10.1021/acs.chemmater.7b04666

[143] L. Tong, P. Wang, A. Chen, F. Qiu, W. Fang et al., Improved electrochemical performance of binder-free multi-layered silicon/carbon thin film electrode for lithium-ion batteries. Carbon **153**, 592–601 (2019). 10.1016/j.carbon.2019.07.067

[144] R. Pfenninger, S. Afyon, I. Garbayo, M. Struzik, J.L.M. Rupp, Lithium titanate anode thin films for Li-ion solid state battery based on garnets. Adv. Funct. Mater. **28**(21), 1800879 (2018). 10.1002/adfm.201800879

[145] X. Song, W. Yu, S. Zhou, L. Zhao, A. Li et al., Enhancement of Mn-doped LiPON electrolyte for higher performance of all-solid-state thin film lithium battery. Mater. Today Phys. **33**, 101037 (2023). 10.1016/j.mtphys.2023.101037

[146] X. He, Y. Ma, J. Liu, J. Wang, X. Hu, H. Dong, X. Huang, Improved electrochemical performance and chemical stability of thin-film lithium phosphorus oxynitride electrolyte by appropriate fluorine plasma treatment. Electrochim. Acta **454**, 142411 (2023). 10.1016/j.electacta.2023.142411

[147] X. Lü, J.W. Howard, A. Chen, J. Zhu, S. Li et al., Antiperovskite Li_3_OCl superionic conductor films for solid-state Li-ion batteries. Adv. Sci. **3**(3), 1500359 (2016). 10.1002/advs.201500359 10.1002/advs.201500359 PMC5067573 27812460

[148] Q. Ling, Z. Yu, H. Xu, G. Zhu, X. Zhang et al., Preparation and electrical properties of amorphous Li-Al-Ti-P-O thin film electrolyte. Mater. Lett. **169**, 42–45 (2016). 10.1016/j.matlet.2016.01.089

[149] S.-P. Song, C. Yang, C.-Z. Jiang, Y.-M. Wu, R. Guo et al., Increasing ionic conductivity in Li_0.33_La_0.56_TiO_3_ thin-films via optimization of processing atmosphere and temperature. Rare Met. **41**(1), 179–188 (2022). 10.1007/s12598-021-01782-5

[150] R. Pfenninger, M. Struzik, I. Garbayo, E. Stilp, J.L.M. Rupp, A low ride on processing temperature for fast lithium conduction in garnet solid-state battery films. Nat. Energy **4**(6), 475–483 (2019). 10.1038/s41560-019-0384-4

[151] J. Li, C. Ma, M. Chi, C. Liang, N.J. Dudney, Solid electrolyte: the key for high‐voltage lithium batteries. Adv. Energy Mater. **5**(4), 1401408 (2015). 10.1002/aenm.201401408

[152] J. Chen, Q. Xia, W. Liu, H. Xia, Enhancing the lithium storage capability of TiO_2_ thin film for all-solid-state microbatteries via amorphous-crystalline heterostructure design. Appl. Phys. Lett. **121**(13), 131901 (2022). 10.1063/5.0117083

[153] Q. Li, W. Liu, J. Wang, Q. Xia, H. Xia, Long-cycle-life Li_2_MnO_3_ thin-film cathode enabled by all-solid-state battery configuration. J. Power. Sources **602**, 234371 (2024). 10.1016/j.jpowsour.2024.234371

[154] J. Casella, J. Morzy, E. Gilshtein, M. Yarema, M.H. Futscher et al., Electrochemical activation of Fe–LiF conversion cathodes in thin-film solid-state batteries. ACS Nano **18**(5), 4352–4359 (2024). 10.1021/acsnano.3c10146 38284312 10.1021/acsnano.3c10146 PMC10851659

[155] J. Qiu, H. Li, Y. Zhao, R. Xu, K. Wei et al., In-situ reconstructed surface/inner-structure synergistic design enabling 4.6 V LiCoO_2_ cathode for all-solid-state thin-film battery. Mater. Today **80**, 342–352 (2024). 10.1016/j.mattod.2024.09.011

[156] X. Liu, L. Zhang, Y. Zheng, Z. Guo, Y. Zhu et al., Uncovering the effect of lattice strain and oxygen deficiency on electrocatalytic activity of perovskite cobaltite thin films. Adv. Sci. **6**(6), 1801898 (2019). 10.1002/advs.201801898 10.1002/advs.201801898 PMC6425498 30937267

[157] J. Qiu, H. Li, T. Wu, Y. He, R. Xu et al., Construction of longitudinal (003) textured low-strain diffusion channel in 4.6 V LiCoO_2_-based all-solid-state thin film battery for microelectronic systems. ACS Energy Lett. **10**, 3249–3258 (2025). 10.1021/acsenergylett.5c01012

[158] M.H. Futscher, T. Amelal, J. Sastre, A. Müller, J. Patidar, A. Aribia, K. Thorwarth, S. Siol, Y.E. Romanyuk, Influence of amorphous carbon interlayers on nucleation and early growth of lithium metal at the current collector-solid electrolyte interface. J. Mater. Chem. A **10**(29), 15535–15542 (2022). 10.1039/d2ta02843c 10.1039/d2ta02843c PMC9337797 35978581

[159] X. Pu, W. Hu, Z.L. Wang, Toward wearable self‐charging power systems: the integration of energy‐harvesting and storage devices. Small **14**(1), 1702817 (2018). 10.1002/smll.201702817 10.1002/smll.201702817 29194960

[160] J. Liu, J. Xu, Y. Lin, J. Li, Y. Lai et al., All-solid-state lithium ion battery: research and industrial prospects. Acta Chim. Sinica **71**(06), 869–878 (2013). 10.6023/a13020170

[161] F. Sandbaumhüter, S.N. Agbo, C.-L. Tsai, O. Astakhov, S. Uhlenbruck, U. Rau, T. Merdzhanova, Compatibility study towards monolithic self-charging power unit based on all-solid thin-film solar module and battery. J. Power. Sources **365**, 303–307 (2017). 10.1016/j.jpowsour.2017.08.103

[162] D. Lau, N. Song, C. Hall, Y. Jiang, S. Lim et al., Hybrid solar energy harvesting and storage devices: the promises and challenges. Mater. Today Energy **13**, 22–44 (2019). 10.1016/j.mtener.2019.04.003

[163] B. Hu, X.-L. Shi, T. Cao, M. Li, W. Chen, W.-D. Liu, W. Lyu, T. Tesfamichael, Z.-G. Chen, Advances in flexible thermoelectric materials and devices fabricated by magnetron sputtering. Small Science **5**(3), 2300061 (2025). 10.1002/smsc.202300061 40655126 10.1002/smsc.202300061 PMC12244512

[164] M.H. Futscher, L. Brinkman, A. Müller, J. Casella, A. Aribia, Y.E. Romanyuk, Monolithically-stacked thin-film solid-state batteries. Commun. Chem. **6**(1), 110 (2023). 10.1038/s42004-023-00901-w 37277459 10.1038/s42004-023-00901-w PMC10241883

[165] F.L. Cras, B. Pecquenard, V. Dubois, V. Phan, D. Guy‐Bouyssou, All‐solid‐state lithium‐ion microbatteries using silicon nanofilm anodes: high performance and memory effect. Adv. Energy Mater. **5**(19), 1501061 (2015). 10.1002/aenm.201501061

[166] X. Wu, L. Wang, W. Gu, J. Wang, Y. Zhuang et al., High-performance 3D stacked micro all-solid-state thin-film lithium-ion batteries based on the stress-compensation effect. Small **20**(25), 2307250 (2024). 10.1002/smll.202307250 10.1002/smll.202307250 38196305

[167] B. Ke, X. Wang, Integratable all-solid-state thin-film microbatteries. Proc. Natl. Acad. Sci. **122**(16), e2415693122 (2025). 10.1073/pnas.2415693122 40249778 10.1073/pnas.2415693122 PMC12037063

[168] M. Koo, K.-I. Park, S.H. Lee, M. Suh, D.Y. Jeon, J.W. Choi, K. Kang, K.J. Lee, Bendable inorganic thin-film battery for fully flexible electronic systems. Nano Lett. **12**(9), 4810–4816 (2012). 10.1021/nl302254v 22845667 10.1021/nl302254v

[169] A.T. Kutbee, M.T. Ghoneim, S.M. Ahmad, M.M. Hussain, Free-form flexible lithium-ion microbattery. IEEE Trans. Nanotechnol. **15**(3), 402–408 (2016). 10.1109/tnano.2016.2537338

[170] R. Shimizu, D. Cheng, J.L. Weaver, M. Zhang, B. Lu, T.A. Wynn, R. Burger, M.-C. Kim, G. Zhu, Y.S. Meng, Unraveling the stable cathode electrolyte interface in all solid-state thin-film battery operating at 5 V. Adv. Energy Mater. **12**(31), 2201119 (2022). 10.1002/aenm.202201119

[171] Z. Wang, D. Santhanagopalan, W. Zhang, F. Wang, H.L. Xin et al., In situ STEM-EELS observation of nanoscale interfacial phenomena in all-solid-state batteries. Nano Lett. **16**, 3760–3767 (2016). 10.1021/acs.nanolett.6b01119 27140196 10.1021/acs.nanolett.6b01119

[172] S. Lou, F. Zhang, C. Fu, M. Chen, Y. Ma, G. Yin, J. Wang, Interface issues and challenges in all-solid-state batteries: lithium, sodium, and beyond. Adv. Mater. **33**(6), 2000721 (2021). 10.1002/adma.202000721 10.1002/adma.202000721 32705725

[173] A. Uhart, J.B. Ledeuil, B. Pecquenard, F. Le Cras, M. Proust et al., Nanoscale chemical characterization of solid-state microbattery stacks by means of auger spectroscopy and ion-milling cross section preparation. ACS Appl. Mater. Interfaces **9**(38), 33238–33249 (2017). 10.1021/acsami.7b07270 28853552 10.1021/acsami.7b07270

[174] K.L. Browning, A.S. Westover, J.F. Browning, M. Doucet, R.L. Sacci et al., In situ measurement of buried electrolyte-electrode interfaces for solid state batteries with nanometer level precision. ACS Energy Lett. **8**(4), 1985–1991 (2023). 10.1021/acsenergylett.3c00488

[175] C. Chen, J.F.M. Oudenhoven, D.L. Danilov, E. Vezhlev, L. Gao, Na. Li, F.M. Mulder, R.-A. Eichel, P.H.L. Notten, Origin of degradation in Si‐based all‐solid‐state Li‐ion microbatteries. Adv. Energy Mater. **8**(30), 1801430 (2018). 10.1002/aenm.201801430

[176] J. Miao, C.V. Thompson, Kinetic study of the initial lithiation of amorphous silicon thin film anodes. J. Electrochem. Soc. **165**(3), A650–A656 (2018). 10.1149/2.1011803jes

[177] Z. Zou, Z. Xiao, Z. Lin, B. Zhang, C. Zhang, F. Wei, Lithium phosphorous oxynitride as an advanced solid‐state electrolyte to boost high‐energy lithium metal battery. Adv. Funct. Mater. **34**(49), 2409330 (2024). 10.1002/adfm.202409330

[178] Z. Wang, J.Z. Lee, H.L. Xin, L. Han, N. Grillon et al., Effects of cathode electrolyte interfacial (CEI) layer on long term cycling of all-solid-state thin-film batteries. J. Power. Sources **324**, 342–348 (2016). 10.1016/j.jpowsour.2016.05.098

[179] L. Yang, X. Li, K. Pei, W. You, X. Liu, H. Xia, Y. Wang, R. Che, Direct view on the origin of high Li^+^ transfer impedance in all-solid-state battery. Adv. Funct. Mater. **31**(35), 2103971 (2021). 10.1002/adfm.202103971

[180] D. Cheng, T. Wynn, B. Lu, M. Marple, B. Han et al., A free-standing lithium phosphorus oxynitride thin film electrolyte promotes uniformly dense lithium metal deposition with no external pressure. Nat. Nanotechnol. **18**(12), 1448–1455 (2023). 10.1038/s41565-023-01478-0 37537275 10.1038/s41565-023-01478-0

[181] V.C. Ferrari, S.B. Lee, G.W. Rubloff, D.M. Stewart, Interface diagnostics platform for thin-film solid-state batteries. Energy Environ. Sci. **18**, 1783–1800 (2025). 10.1039/d4ee03915g

[182] J. Sastre, X. Chen, A. Aribia, A.N. Tiwari, Y.E. Romanyuk, Fast charge transfer across the Li_7_La_3_Zr_2_O_12_ solid electrolyte/LiCoO_2_ cathode interface enabled by an interphase-engineered all-thin-film architecture. ACS Appl. Mater. Interfaces **12**(32), 36196–36207 (2020). 10.1021/acsami.0c09777 32672438 10.1021/acsami.0c09777

[183] H.Y. Park, S.R. Lee, Y.J. Lee, B.W. Cho, W.I. Cho, Bias sputtering and characterization of LiCoO_2_ thin film cathodes for thin film microbattery. Mater. Chem. Phys. **93**(1), 70–78 (2005). 10.1016/j.matchemphys.2005.02.024

[184] Y. He, Q. Xia, W. Liu, C. Wu, J. Wang, Yu. Cai, Y. Guo, F. Zan, J. Xu, H. Xia, Toward high‐performance all‐solid‐state thin film FeO_x_S_y_/LiPON/Li microbatteries via dual‐interface modification. Adv. Funct. Mater. **34**(19), 2310876 (2024). 10.1002/adfm.202310876

[185] C.-F. Xiao, J.H. Kim, S.-H. Cho, Y.C. Park, M.J. Kim et al., Ensemble design of electrode-electrolyte interfaces: toward high-performance thin-film all-solid-state Li-metal batteries. ACS Nano **15**(3), 4561–4575 (2021). 10.1021/acsnano.0c08691 33629830 10.1021/acsnano.0c08691

[186] C. Yada, A. Ohmori, K. Ide, H. Yamasaki, T. Kato, T. Saito, F. Sagane, Y. Iriyama, Dielectric modification of 5 V-class cathodes for high-voltage all-solid-state lithium batteries. Adv. Energy Mater. **4**(9), 1301416 (2014). 10.1002/aenm.201301416

[187] F.S. Gittleson, F. El Gabaly, Non-faradaic Li^+^ migration and chemical coordination across solid-state battery interfaces. Nano Lett. **17**(11), 6974–6982 (2017). 10.1021/acs.nanolett.7b03498 29058442 10.1021/acs.nanolett.7b03498

[188] Y. Iriyama, K. Nishimoto, C. Yada, T. Abe, Z. Ogumi et al., Charge-transfer reaction at the lithium phosphorus oxynitride glass electrolyte/lithium manganese oxide thin-film interface and its stability on cycling. J. Electrochem. Soc. **153**(5), A821 (2006). 10.1149/1.2178647

[189] Y. Iriyama, T. Kako, C. Yada, T. Abe, Z. Ogumi, Reduction of charge transfer resistance at the lithium phosphorus oxynitride/lithium cobalt oxide interface by thermal y treatment. J. Power. Sources **146**(1−2), 745–748 (2005). 10.1016/j.jpowsour.2005.03.073

[190] Y. Yang, S. Jeong, L. Hu, H. Wu, S.W. Lee et al., Transparent lithium-ion batteries. Proc. Natl. Acad. Sci. **108**(32), 13013–13018 (2011). 10.1073/pnas.1102873108 21788483 10.1073/pnas.1102873108 PMC3156205

[191] J.F.M. Oudenhoven, Loïc.. Baggetto, P.H.L. Notten, All-solid-state lithium-ion microbatteries: a review of various three-dimensional concepts. Adv. Energy Mater. **1**(1), 10–33 (2011). 10.1002/aenm.201000002

[192] T.S. Arthur, D.J. Bates, N. Cirigliano, D.C. Johnson, P. Malati, J.M. Mosby, E. Perre, M.T. Rawls, A.L. Prieto, B. Dunn, Three-dimensional electrodes and battery architectures. MRS Bull. **36**(7), 523–531 (2011). 10.1557/mrs.2011.156

[193] A. Pearse, T. Schmitt, E. Sahadeo, D.M. Stewart, A. Kozen, K. Gerasopoulos, A.A. Talin, S.B. Lee, G.W. Rubloff, K.E. Gregorczyk, Three-dimensional solid-state lithium-ion batteries fabricated by conformal vapor-phase chemistry. ACS Nano **12**(5), 4286–4294 (2018). 10.1021/acsnano.7b08751 29688704 10.1021/acsnano.7b08751

[194] M. Létiche, E. Eustache, J. Freixas, A. Demortière, V. De Andrade, L. Morgenroth, P. Tilmant, F. Vaurette, D. Troadec, P. Roussel, T. Brousse, C. Lethien, Atomic layer deposition of functional layers for on chip 3D Li-ion all solid state microbattery. Adv. Energy Mater. **7**(2), 1601402 (2017). 10.1002/aenm.201601402

[195] M. Hallot, V. Nikitin, O.I. Lebedev, R. Retoux, D. Troadec, V. De Andrade, P. Roussel, C. Lethien, 3D LiMn_2_O_4_ thin film deposited by ALD: a road toward high‐capacity electrode for 3D Li‐ion microbatteries. Small **18**(14), 2107054 (2022). 10.1002/smll.202107054 10.1002/smll.202107054 35174974

[196] A.A. Talin, D. Ruzmetov, A. Kolmakov, K. McKelvey, N. Ware et al., Fabrication, testing, and simulation of all-solid-state three-dimensional Li-ion batteries. ACS Appl. Mater. Interfaces **8**(47), 32385–32391 (2016). 10.1021/acsami.6b12244 27933836 10.1021/acsami.6b12244 PMC5526591

[197] D. Ruzmetov, V.P. Oleshko, P.M. Haney, H.J. Lezec, K. Karki et al., Electrolyte stability determines scaling limits for solid-state 3D Li ion batteries. Nano Lett. **12**(1), 505–511 (2012). 10.1021/nl204047z 22185512 10.1021/nl204047z

[198] J. Zhang, W. Li, Z. Liu, Z. Huang, H. Wang, B. Ke, Lu. Xue, H. Jia, X. Wang, All‐solid‐state thin‐film lithium‐selenium batteries. Adv. Funct. Mater. **35**(38), 2503732 (2025). 10.1002/adfm.202503732

[199] Q. Xia, N. Jabeen, S.V. Savilov, S.M. Aldoshin, H. Xia, Black mesoporous Li_4_Ti_5_O_12−δ_ nanowall arrays with improved rate performance as advanced 3D anodes for microbatteries. J. Mater. Chem. A **4**(44), 17543–17551 (2016). 10.1039/c6ta06699b

[200] J. Xie, J.F.M. Oudenhoven, D. Li, C. Chen, R.-A. Eichel et al., High power and high capacity 3D-structured TiO_2_ electrodes for lithium-ion microbatteries. J. Electrochem. Soc. **163**(10), A2385 (2016). 10.1149/2.1141610jes

[201] Y. Wang, T. Wang, P. Da, M. Xu, H. Wu et al., Silicon nanowires for biosensing, energy storage, and conversion. Adv. Mater. **25**(37), 5177–5195 (2013). 10.1002/adma.201301943 23828226 10.1002/adma.201301943

[202] S. Sun, Q. Xia, J. Liu, J. Xu, F. Zan et al., Self-standing oxygen-deficient α-MoO_3−x_ nanoflake arrays as 3D cathode for advanced all-solid-state thin film lithium batteries. J. Materiomics **5**(2), 229–236 (2019). 10.1016/j.jmat.2019.01.001

[203] Z. Qi, J. Tang, J. Huang, D. Zemlyanov, V.G. Pol et al., Li_2_MnO_3_ thin films with tilted domain structure as cathode for Li-ion batteries. ACS Appl. Energy Mater. **2**(5), 3461–3468 (2019). 10.1021/acsaem.9b00259

[204] Z. Qi, J. Tang, S. Misra, C. Fan, P. Lu et al., Enhancing electrochemical performance of thin film lithium ion battery via introducing tilted metal nanopillars as effective current collectors. Nano Energy **69**, 104381 (2020). 10.1016/j.nanoen.2019.104381

[205] B. Xiao, Q. Tang, X. Dai, F. Wu, H. Chen et al., Enhanced interfacial kinetics and high rate performance of LiCoO_2_ thin-film electrodes by Al doping and in situ Al_2_O_3_ coating. ACS Omega **7**(35), 31597–31606 (2022). 10.1021/acsomega.2c04665 36092563 10.1021/acsomega.2c04665 PMC9453800

[206] M. Rawlence, A.N. Filippin, A. Wäckerlin, T.-Y. Lin, E. Cuervo-Reyes et al., Effect of gallium substitution on lithium-ion conductivity and phase evolution in sputtered Li_7−3__*x*_Ga_*x*_La_3_Zr_2_O_12_ thin films. ACS Appl. Mater. Interfaces **10**(16), 13720–13728 (2018). 10.1021/acsami.8b03163 29608054 10.1021/acsami.8b03163

[207] J. Sastre, T.-Y. Lin, A.N. Filippin, A. Priebe, E. Avancini et al., Aluminum-assisted densification of cosputtered lithium garnet electrolyte films for solid-state batteries. ACS Appl. Energy Mater. **2**(12), 8511–8524 (2019). 10.1021/acsaem.9b01387

[208] J. Sastre, A. Priebe, M. Döbeli, J. Michler, A.N. Tiwari, Y.E. Romanyuk, Lithium garnet Li_7_La_3_Zr_2_O_12_ electrolyte for all‐solid‐state batteries: closing the gap between bulk and thin film Li‐ion conductivities. Adv. Mater. Interfaces **7**(17), 2000425 (2020). 10.1002/admi.202000425

[209] J. Sastre, M.H. Futscher, L. Pompizi, A. Aribia, A. Priebe et al., Blocking lithium dendrite growth in solid-state batteries with an ultrathin amorphous Li–La–Zr–O solid electrolyte. Commun. Mater. **2**(1), 76 (2021). 10.1038/s43246-021-00177-4

[210] H. Liu, H. Sun, W. Hua, X. Liu, W. Liu et al., Roll-to-roll coating to processing large-area Zinc anodes toward fast and dendrite-free deposition. Adv. Funct. Mater. (2025). 10.1002/adfm.202509206

[211] M. Zhu, O.G. Schmidt, Tiny robots and sensors need tiny batteries—here’s how to do it. Nature **589**(7841), 195–197 (2021). 10.1038/d41586-021-00021-2 33442035 10.1038/d41586-021-00021-2

[212] D. Cheng, K. Tran, S. Rao, Z. Wang, R. Van Der Linde et al., Manufacturing scale-up of anodeless solid-state lithium thin-film batteries for high volumetric energy density applications. ACS Energy Lett. **8**(11), 4768–4774 (2023). 10.1021/acsenergylett.3c01839

[213] M.-S. Jung, M.-W. Moon, J.-H. Seo, Y.-C. Joo, I.-S. Choi, Robust Si anode design for Li-ion battery on polyimide substrate: villus-like polymer/Si core/shell hybrid nano-structure. ECS Meet. Abstr. **14**, 935 (2013). 10.1149/MA2013-02/14/953

[214] J. Glenneberg, F. Andre, I. Bardenhagen, F. Langer, J. Schwenzel, R. Kun, A concept for direct deposition of thin film batteries on flexible polymer substrate. J. Power. Sources **324**, 722–728 (2016). 10.1016/j.jpowsour.2016.06.007

[215] J. Glenneberg, G. Kasiri, I. Bardenhagen, F. La Mantia, M. Busse et al., Investigations on morphological and electrochemical changes of all-solid-state thin film battery cells under dynamic mechanical stress conditions. Nano Energy **57**, 549–557 (2019). 10.1016/j.nanoen.2018.12.070

[216] X. Dai, Z. Wang, J. Li, F. Wu, Y. Mai, X. Dong, Controllable preparation and electrochemical properties of in-situ annealed LiCoO_2_ films with a specific crystalline orientation on stainless steel substrates. Solid State Ionics **365**, 115658 (2021). 10.1016/j.ssi.2021.115658

[217] K.A. Striebel, C.Z. Deng, S.J. Wen, E.J. Cairns, Electrochemical behavior of LiMn_2_O_4_ and LiCoO_2_ thin films produced with pulsed laser deposition. J. Electrochem. Soc. **143**(6), 1821–1827 (1996). 10.1149/1.1836910

[218] S.B. Tang, M.O. Lai, L. Lu, Properties of nano-crystalline LiMn_2_O_4_ thin films deposited by pulsed laser deposition. Electrochim. Acta **52**(3), 1161–1168 (2006). 10.1016/j.electacta.2006.07.014

[219] R. Chen, W. Liang, H. Zhang, F. Wu, L. Li, Preparation and performance of novel LLTO thin film electrolytes for thin film lithium batteries. Chin. Sci. Bull. **57**(32), 4199–4204 (2012). 10.1007/s11434-012-5292-y

[220] A. Sepúlveda, J. Speulmanns, P.M. Vereecken, Bending impact on the performance of a flexible Li_4_Ti_5_O_12_-based all-solid-state thin-film battery. Sci. Technol. Adv. Mater. **19**(1), 454–464 (2018). 10.1080/14686996.2018.1468199 29868149 10.1080/14686996.2018.1468199 PMC5974753

[221] S.-W. Song, S.-J. Hong, H.Y. Park, Y.C. Lim, K.C. Lee, Cycling-driven structural changes in a thin-film lithium battery on flexible substrate. Electrochem. Solid-State Lett. **12**(8), A159 (2009). 10.1149/1.3139530

[222] S.-W. Song, K.-C. Lee, H.-Y. Park, High-performance flexible all-solid-state microbatteries based on solid electrolyte of lithium boron oxynitride. J. Power. Sources **328**, 311–317 (2016). 10.1016/j.jpowsour.2016.07.114

[223] H. Ge, H. Tian, H. Song, D. Liu, S. Wu, X. Shi, X. Gao, Li. Lv, X.-M. Song, Study on the energy band structure and photoelectrochemical performances of spinel Li_4_Ti_5_O_12_. Mater. Res. Bull. **61**, 459–462 (2015). 10.1016/j.materresbull.2014.10.064

[224] H. Lee, K.-B. Kim, J.-W. Choi, Transparent SiN thin-film anode for thin-film batteries by reactive sputtering at room temperature. Chem. Eng. J. **401**, 126086 (2020). 10.1016/j.cej.2020.126086

[225] M. Roeder, A.B. Beleke, U. Guntow, J. Buensow, A. Guerfi, U. Posset, H. Lorrmann, K. Zaghib, G. Sextl, Li_4_Ti_5_O_12_ and LiMn_2_O_4_ thin-film electrodes on transparent conducting oxides for all-solid-state and electrochromic applications. J. Power. Sources **301**, 35–40 (2016). 10.1016/j.jpowsour.2015.09.063

[226] S. Pat, H. HakanYudar, Ş Korkmaz, S. Özen, R. Mohammadigharehbagh, Z. Pat, The microstructural, surface, optical and electrochemical impedance spectroscopic study of the semitransparent all-solid-state thin film battery. Mater. Res. Express **6**(1), 015503 (2018). 10.1088/2053-1591/aae4aa

[227] S. Pat, S. Özen, H.H. Yudar, Ş Korkmaz, Z. Pat, The transparent all-solid-state rechargeable micro-battery manufacturing by rf magnetron sputtering. J. Alloys Compd. **713**, 64–68 (2017). 10.1016/j.jallcom.2017.04.169

[228] S. Oukassi, L. Baggetto, C. Dubarry, L.L. Van-Jodin, S. Poncet, L. Le Van-Jodin, R. Salot, Transparent thin film solid-state lithium ion batteries. ACS Appl. Mater. Interfaces **11**(1), 683–690 (2019). 10.1021/acsami.8b16364 30525408 10.1021/acsami.8b16364

[229] H. Lee, S. Kim, K.-B. Kim, J.-W. Choi, Scalable fabrication of flexible thin-film batteries for smart lens applications. Nano Energy **53**, 225–231 (2018). 10.1016/j.nanoen.2018.08.054

[230] G.A. Landis, Conf. Record of the Twenty-Ninth IEEE Photovoltaic Specialists Conf. (Cat. No.02CH37361), IEEE. 1539−1540 (2002). 10.1109/pvsc.2002.1190441

[231] G. Chen, M. Fojtik, D. Kim, D. Fick, J. Park et al., Millimeter-scale nearly perpetual sensor system with stacked battery and solar cells. In: *2010 IEEE International Solid-State Circuits Conference. (ISSCC)*, IEEE. 288−289 (2010). 10.1109/isscc.2010.5433921

[232] M. Fojtik, D. Kim, G. Chen, Y.-S. Lin, D. Fick, J. Park, M. Seok, M.-T. Chen, Z. Foo, D. Blaauw, D. Sylvester, A millimeter-scale energy-autonomous sensor system with stacked battery and solar cells. IEEE J. Solid-State Circuits **48**(3), 801–813 (2013). 10.1109/jssc.2012.2233352

[233] T. Kuriyama, A. Suzuki, Y. Okamoto, I. Kimura, Y. Morikawa et al., A micromachined all-solid on-chip thin-film battery towards uninterruptible photovoltaic cells. In *2018 Symposium on Design, Test, Integration & Packaging of MEMS and MOEMS (DTIP)*, IEEE. 1−4 (2018). 10.1109/DTIP.2018.8394215

[234] S.R. Anton, A. Erturk, D.J. Inman, Multifunctional self-charging structures using piezoceramics and thin-film batteries. Smart Mater. Struct. **19**(11), 115021 (2010). 10.1088/0964-1726/19/11/115021

[235] H. Lhermet, C. Condemine, M. Plissonnier, R. Salot, P. Audebert, M. Rosset, Efficient power management circuit: from thermal energy harvesting to above-IC microbattery energy storage. IEEE J. Solid-State Circuits **43**(1), 246–255 (2008). 10.1109/jssc.2007.914725

[236] W.-Y. Liu, Z.-W. Fu, C.-L. Li, Q.-Z. Qin, Lithium phosphorus oxynitride thin film fabricated by a nitrogen plasma-assisted deposition of E-beam reaction evaporation. Electrochem. Solid-State Lett. **7**(9), J36 (2004). 10.1149/1.1778934

[237] B. Wang, J.B. Bates, F.X. Hart, B.C. Sales, R.A. Zuhr et al., Characterization of thin-film rechargeable lithium batteries with lithium cobalt oxide cathodes. J. Electrochem. Soc. **143**(10), 3203 (1996). 10.1149/1.1837188

